# The overestimation of the effect sizes of psychotherapies for depression in waitlist controlled trials: a meta-analytic comparison with usual care controlled trials

**DOI:** 10.1017/S2045796024000611

**Published:** 2024-11-06

**Authors:** Pim Cuijpers, Clara Miguel, Mathias Harrer, Marketa Ciharova, Eirini Karyotaki

**Affiliations:** 1 Department of Clinical, Neuro and Developmental Psychology, Amsterdam Public Health research institute, Vrije Universiteit Amsterdam, Amsterdam, the Netherlands; 2 Babeș-Bolyai University, International Institute for Psychotherapy, Cluj-Napoca, Romania; 3 Psychology & Digital Mental Health Care, Technical University Munich, Munich, Germany

**Keywords:** cognitive therapy, depression, randomized controlled trials, systematic reviews

## Abstract

**Aims:**

There is considerable evidence that waiting list (WL) control groups overestimate the effect sizes of psychotherapies for depression. It is not clear, however, what are the exact causes for this overestimation. We decided to conduct a meta-analytic study to compare trials on psychotherapy for depression with a WL control group against trials with a care-as-usual (CAU) control group.

**Methods:**

We used an existing meta-analytic database of randomized trials comparing psychological treatments of adult depression with control groups and selected trials using a WL or a CAU control group. We used subgroup and meta-regression analyses to examine differences in effect sizes between WL and CAU controlled trials.

**Results:**

We included 333 randomized controlled trials (472 comparisons; total number participants: 41,480), 141 with a WL and 195 with a CAU control group (3 included both). We found several significant differences between WL and CAU controlled trials (in type of therapy examined, treatment format, recency, target group, recruitment strategy, number of treatment arms and number of depression outcome measures). The overall effect size indicating the difference between treatment and control at post-test for all comparisons was *g* = 0.77 (95% confidence interval [CI]: 0.71; 0.84) with high heterogeneity (*I*^2^ = 84; 95% CI: 82; 85). A highly significant difference was observed between studies with a CAU control group (*g* = 0.63; 95% CI: 0.55; 0.71; *I*^2^ = 85; 95% CI: 83; 86) and studies with a WL (*g* = 0.95; 95% CI: 0.85; 1.04; *I*^2^ = 80; 95% CI: 78; 82; *p* for difference < 0.001). This difference remained significant in all sensitivity analyses, including a meta-regression analysis in which we adjusted for all differences in characteristics of studies with a WL versus CAU control group. We also found that pre-post effect sizes in WL control conditions (*g* = 0.37; 95% CI: 0.28; 0.46) were significantly smaller than change within CAU conditions (*g* = 0.64; 95% CI: 0.50; 0.78). We found few indications that pre-post effect sizes within therapy conditions differed between WL and CAU controlled trials.

**Conclusions:**

WL control conditions considerably overestimate the effect sizes of psychological treatments, compared to trials using CAU control conditions. This overestimation is probably caused by a smaller improvement within the WL condition compared to the improvement in the CAU condition. WL control conditions should be avoided in randomized trials examining psychological treatments of adult depression.

## Introduction

It is well-established that psychological interventions are effective in the treatment of depression (Cuijpers *et al.*, 2023a). Several types of therapy have been found to have small to moderate effects on depression, including cognitive behaviour therapy (CBT), interpersonal psychotherapy (IPT), behavioural activation therapy, but also for example psychodynamic therapy and life review therapy (Cuijpers *et al.*, 2021a). The effects of these interventions are typically examined in randomized controlled trials comparing these interventions with waiting lists (WLs), care-as-usual (CAU) or other control conditions such as attention placebo (Cuijpers *et al.*, 2020). Meta-analyses of these trials typically show that the effect sizes of therapies are larger when they are compared to WL control groups, compared to trials in which the therapies are compared to other control groups (Cuijpers *et al.*, 2016; Furukawa *et al.*, 2014; Hesser *et al.*, 2011; Michopoulos *et al.*, 2021). WL control groups also have been found to have an inflated effect size in other conditions (e.g., Laws *et al.*, 2022; Young, 2006; Zhu *et al.*, 2014), although this may vary across conditions (Cunningham *et al.*, 2013). However, in depression this inflating effect size is well-established. In a large network meta-analysis of psychological treatments of depression we found a standardized mean difference (SMD) of 0.29 (0.14–0.45) of being in a WL control group compared to being in a CAU control group (Cuijpers *et al.*, 2021a), which is comparable to the difference between antidepressants and placebo (SMD = 0.30; Cipriani *et al.*, 2018).

It is not clear why WLs inflate treatment effect sizes. It has been suggested that patients on WLs actually ‘wait’ to change until they receive the intervention (Miller and Rollnick, 2002). This may result in lower ‘spontaneous recovery’ rates in WL conditions. Expectancies have also been suggested as a mediating variable (Cunningham and McCambridge, 2013), with higher expectancies in people in WL conditions, compared to other control conditions. Furthermore, there is also evidence that patients are disappointed when assigned to control conditions (Lindström *et al.*, 2010; Skingley *et al.*, 2014), and this disappointment may be lower in WL conditions compared to other control groups, because these participants do get the intervention after the waiting time.

Overall, there is very little research on WLs in clinical settings (Cunningham and McCambridge, 2013), and many questions have not yet been answered. To the best of our knowledge no previous meta-analysis has compared the characteristics of trials using WL control conditions to trials with CAU control conditions, while such trials may have different characteristics that could explain the superior effect sizes for WL controlled trials. Also, it is not clear whether the superior effect sizes found in WL controlled trials are caused by lower response rates in the control conditions or by higher response rates in the treatment conditions.

We decided therefore to conduct a meta-analysis with three goals: (1) to compare the characteristics of trials using WL control groups to trials with CAU; (2) to confirm the findings from previous meta-analyses that WL control groups result in larger effect sizes than CAU control groups and examine if this difference remains significant after adjusting for the characteristics of the participants, treatments and studies; (3) to compare response rates and pre-post effect sizes in control and treatment conditions to examine whether the superior effect sizes of WL controlled trials is related to smaller effects in the control conditions or larger effects in the treatment conditions.

## Methods

### Identification and selection of studies

This study is part of a larger meta-analytic project on psychological treatments of depression (registered at the Open Science Framework; Cuijpers *et al.*, 2022; https://doi.org/10.17605/OSF.IO/825C6). The general methods of the project have been described in a separate paper (Harrer et al., submitted) and supplemental materials are available at the website of the project (http://www.metapsy.org). This database has been used in a series of earlier published meta-analyses (Cuijpers *et al.*, 2023b). The protocol for the current review and meta-analysis has been published at the Open Science Framework (Cuijpers, 2023c; https://doi.org/10.17605/OSF.IO/GWPV2).

The studies included in the current study were identified through the larger, already existing database of randomized trials on the psychological treatment of depression. For this database we searched four major bibliographical databases (PubMed, PsycINFO, Embase and the Cochrane Library) by combining index and free terms indicative of depression and psychotherapies, with filters for randomized controlled trials. The full search strings can be found at the website of the project (www.metapsy.org and docs.metapsy.org/databases/depression-psyctr/). Furthermore, we checked the references of earlier meta-analyses on psychological treatments of depression. The database is updated every 4 months and was developed through a comprehensive literature search (from 1966 to 1 May 2023). All records were screened by two independent researchers and all papers that could possibly meet inclusion criteria according to one of the researchers were retrieved as full-text. The decision to include or exclude a study in the database was also done by the two independent researchers, and disagreements were resolved through discussion.

For the current meta-analysis, we selected randomized controlled trials in which a psychological treatment of depression was compared with a WL or a CAU control group. We only included trials in adults. We allowed trials in any treatment format, as long as there was human support available (including individual, group, digital or non-digital guided self-help, telephone). We excluded trials in which no human support was given (Cuijpers *et al.*, 2019), and studies in inpatients (Cuijpers *et al.*, 2021b). Depression could be defined as meeting criteria for a depressive disorder according to a diagnostic interview or as a score above the cut-off on a validated self-report depression measure.

### Quality assessment and data extraction

We assessed the validity of included studies using four criteria of the ‘Risk of bias’ (RoB) assessment tool, version 1, developed by the Cochrane Collaboration (Higgins *et al.*, 2011). We used version 1 of this tool because this meta-analysis is included in the broader meta-analytic project of psychological treatments of depression (Sterne *et al.*, 2019). The RoB tool assesses possible sources of bias in randomized trials, including the adequate generation of allocation sequence; the concealment of allocation to conditions; the prevention of knowledge of the allocated intervention (masking of assessors); and dealing with incomplete outcome data (this was assessed as positive when intention-to-treat analyses were conducted, meaning that all randomized patients were included in the analyses). We considered trials as having low risk of bias when they scored positive on all four domains. Assessment of the validity of the included studies was conducted by two independent researchers, and disagreements were solved through discussion.

We also coded participant characteristics (diagnostic method for participant inclusion; recruitment method; target group; mean age; the proportion of women); characteristics of the psychological treatments in the experimental conditions (type of therapy (according to the classification developed for this project earlier, 2020; Cuijpers *et al.*, 2008); treatment format; and number of sessions); and general characteristics of the studies (publication year; the country where the study was conducted; number of experimental conditions in the trial, number of outcome measures). The details of these characteristics can be found at the website of the project (docs.metapsy.org/databases/depression-psyctr).

### Outcome measures

For each comparison between a psychological treatment and a control condition, the small-sample bias corrected SMD between the two groups at post-test was calculated (Hedges’ *g*). When means and standard deviations were not reported, we used change scores, binary outcomes (that were converted to Hedges’ *g)* or other statistics (e.g., *p* value, *t* value) to calculate the effect size. We used one depression measure from each study for the calculation of effect sizes, based on the frequency of the use of the measures.

We also calculated pre-post effect sizes within the treatment and, separately, within the control groups, as the difference between the mean pre-test and the mean post-test score, divided by the standard deviation (SD) of the pre-test. We used the SD of the pre-test to avoid a potential impact of the usual care on the post-test SD (Harrer *et al.*, 2021, chap. 3.3.1.3). We assumed a correlation of 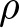 = 0.8 between pre- and post-test.

Treatment response was a secondary outcome. It was defined as the number of patients with 50% symptom reduction between baseline and post-test, divided by the total number of patients. Patients randomized but not included in the analyses of responders in the original reports were assumed to be non-responders and were included in the analyses to abide by the intention-to-treat principle. We calculated response rates with a well-validated method, using the baseline mean, and the mean, standard deviation and number of patients at post-test (Furukawa 2005).

### Meta-analyses

#### Differences between characteristics of WL and CAU controlled trials

To compare baseline differences between trials using WL control groups and those with CAU control groups, we conducted bivariable analyses with *χ*^2^-tests for categorical variables and *t*-tests for continuous variables. For comparing baseline severity across trials in primary and outpatient care, we converted the most common depression measures (Beck Depression Inventory (BDI; Beck *et al.*, 1961), the Beck Depression Inventory II (BDI-II; Beck *et al.*, 1996), Montgomery–Åsberg Depression Rating Scale (MADRS; Williams *et al.*, 2008), Patient Health Questionnaire-9 (PHQ-9; Kroenke *et al.*, 2001), Edinburgh Postnatal Depression Scale (EPDS; Cox *et al.*, 1987) to the Hamilton Depression Rating Scale-17 (HDRS-17; Hamilton, 1960), using established conversion methods (Furukawa *et al.*, 2020; Leucht *et al.*, 2018, Wahl *et al.*, 2014)).

#### The differences between the effect sizes in trials using WL and CAU control conditions

These analyses were conducted using the ‘metapsyTools’ package in R (version 4.1.1; Harrer *et al.*, 2022) and Rstudio (version 1.1.463 for Mac). The ‘metapsyTools’ package was specifically developed for our meta-analytic project and imports functionality of the ‘meta’ (Balduzzi *et al.*, 2019), ‘metafor’ (Viechtbauer, 2010) and ‘dmetar’ (Harrer *et al.*, 2019) packages.

We first pooled the effect sizes of all trials (indicating the difference between treatment and control conditions at post-test) using a random effects model. Between-study heterogeneity variance (components) 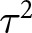 were estimated using restricted maximum likelihood (REML). We applied the Knapp–Hartung method to obtain robust confidence intervals (CIs) and significance tests of the overall effect (IntHout *et al.*, 2014). We calculated the *I*^2^-statistic and its 95% CI, which is an indicator of heterogeneity in percentages (Higgins *et al.*, 2003).

We conducted several sensitivity analyses. First, we pooled effects while excluding outliers, using the ‘non-overlapping confidence intervals’ approach (Harrer *et al.*, 2021). Second, we estimated the pooled effect using only studies with low risk of bias. We also used Duval and Tweedie’s trim and fill procedure (Duval and Tweedie, 2000) to adjust for potential publication bias.

We tested the difference between WL and CAU controlled trials in a subgroup analysis, using a mixed effects model with group-specific 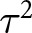 estimates. We also conducted a meta-regression analysis with the effect size as the dependent variable. As predictors we entered a dummy variable for the type of control group (WL vs CAU) and the characteristics of the studies that were found to significantly differ between WL and CAU conditions.

We also compared the pre-post effect sizes within the control conditions, as well as the effect sizes within the treatment conditions. We examined the difference between WL and CAU control conditions with a subgroup analyses, and ran meta-regression analyses with the same predictors as the previous meta-regression analyses described.

#### Differences in response rates between conditions

We first pooled rates for response, separately for the treatment and the control conditions, across all included trials using the ‘meta’ package in R (version 3.6.3). We synthesized the binary outcome data using a normal-normal random-effects pooling models after performing a logit transformation of the response rates. The summary results were then re-converted to the raw proportion scale, and the estimates and their 95% CIs are presented. The between-study heterogeneity variance was approximated using the REML estimator.

We conducted several sensitivity analyses, one in which we excluded outliers, one in which we only examined studies with low risk of bias and a third in which we adjusted for small study effects through the Duval and Tweedie trim-and-fill procedure. Differences between studies with WL and CAU control conditions were examined with subgroup analyses and meta-regression analyses.

## Results

### Selection and inclusion of studies

**Figure 1.**
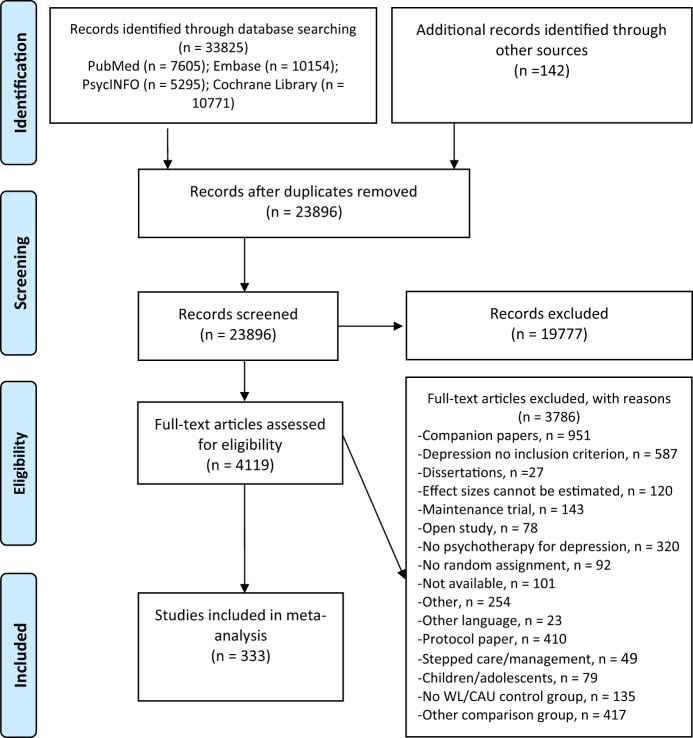
Flowchart of the inclusion of studies.

After examining a total of 33,967 records (23,896 after the removal of duplicates), we retrieved 4,119 full-text papers for further consideration of which 3,786 were excluded. The PRISMA flowchart describing the inclusion process is presented in Figure 1. A total of 333 randomized controlled trials (472 comparisons between therapy and control conditions) were included. The trials included 41,480 participants (18,104 in the control conditions and 23,376 in the treatment conditions). The references of the 333 trials are given in Appendix A and a summary of key characteristics in Appendix B.

### Characteristics of included studies

The aggregated characteristics of the included studies are presented in Table 1. The majority of studies included an arm with CBT (55% of the studies) and none of the other therapies were examined in more than 9% of the trials. Overall, 38% of the studies examined individual therapies, 32% group therapies, 5% telephone therapy, 22% guided self-help and 9% used a mixed or other format. The mean number of sessions across all interventions was 8.68 (SD = 4.59).

**Table 1.** Selected characteristics of randomized trials comparing psychotherapies with waiting list (WL) and care-as-usual (CAU) control groups^a^

		Difference between CAU and WL controlled trials				
		CAU (*N* = 192)	WL (*N* = 138)	All (*N* = 330)^a^	*p*	All trials (*N* = 333)^b^
Therapy type^c^	CBT	97 (50.5)	85 (61.6)	182 (55.2)	0.06	184 (55.3)
	IPT	18 (9.4)	3 (2.2)	21 (6.4)	0.02	21 (6.3)
	Third wave therapies	10 (5.2)	18 (13.0)	28 (8.5)	0.02	28 (8.4)
	BAT	20 (10.4)	10 (7.2)	30 (9.1)	0.43	30 (9.0)
	Psychodynamic^b^	7 (3.6)	2 (1.4)	9 (2.7)	0.39	9 (2.7)
	Life review therapy	7 (3.6)	6 (4.3)	13 (3.9)	0.97	13 (3.9)
	Problem-solving	11 (5.7)	11 (8.0)	22 (6.7)	0.56	23 (6.9)
	Supportive therapy^b^	11 (5.7)	3 (2.2)	14 (4.2)	0.19	14 (4.2)
	Other therapy	25 (13.0)	19 (13.8)	44 (13.3)	0.97	44 (13.2)
Format^c,d^	Individual	90 (46.9)	35 (25.4)	125 (37.9)	≤0.001	127 (38.1)
	Group	54 (28.1)	53 (38.4)	107 (32.4)	0.06	107 (32.1)
	Telephone^b^	15 (7.8)	2 (1.4)	17 (5.2)	0.02	17 (5.1)
	Guided self-help	20 (10.4)	51 (37.0)	71 (21.5)	≤0.001	72 (21.6)
	Mixed/other	19 (9.9)	11 (8.0)	30 (9.1)	0.68	30 (9.0)
*N sessions (M, SD)*		*8.82 (5.24)*	*8.46 (3.41)*	*8.67 (4.42)*	*0.47*	*8.68 (4.59)*
Number of conditions	Two	173 (90.1)	91 (65.9)	264 (80.0)	0.001	264 (79.3)
	More than two	19 (9.9)	47 (34.1)	66 (20.0)		69 (20.7)
Risk of bias	Low	74 (38.5)	46 (33.3)	120 (36.4)	0.39	121 (36.3)
	Other	118 (61.5)	92	(66.7)		212 (63.7)
*N* depression measures	One	151 (78.6)	89 (64.5)	240 (72.7)	0.006	242 (72.7)
	More than one	41 (21.4)	49 (35.5)	90 (27.3)		91 (27.3)
Country	North America	53 (27.6)	46 (33.3)	99 (30.0)	0.17	100 (30.0)
	Europe	68 (35.4)	57 (41.3)	125 (37.9)		127 (38.1)
	East Asia	25 (13.0)	13 (9.4)	38 (11.5)		38 (11.4)
	Other	46 (24.0)	22 (15.9)	68 (20.6)		68 (20.4)
*Publication year (M, SD)*		*2014 (7.14)*	*2010 (11.73)*	*2012 (9.75)*	*≤0.001*	*2012 (9.54)*
Diagnosis	Mood disorder	85 (44.3)	61 (44.2)	146 (44.2)	1.00	146 (43.8)
	Cut-off	107 (55.7)	77 (55.8)	184 (55.8)		187 (56.2)
Target group	Adults in general	55 (28.6)	68 (49.3)	123 (37.3)	≤0.001	123 (36.9)
	General medical	46 (24.0)	28 (20.3)	74 (22.4)		75 (22.5)
	Older adults	14 (7.3)	11 (8.0)	25 (7.6)		25 (7.5)
	Perinatal depression	39 (20.3)	8 (5.8)	47 (14.2)		47 (14.1)
	Students	8 (4.2)	10 (7.2)	18 (5.5)		19 (5.7)
	Other	30 (15.6)	13 (9.4)	43 (13.0)		44 (13.2)
Recruitment	Community	34 (17.7)	88 (63.8)	122 (37.0)	≤0.001	124 (37.2)
	Clinical	56 (29.2)	25 (18.1)	81 (24.5)		81 (24.3)
	Other	102 (53.1)	25 (18.1)	127 (38.5)		128 (38.4)
*Mean age (M, SD)*		*43.82 (15.48)*	*42.38 (13.20)*	*43.20 (16.23)*	*0.39*	*43.21 (14.59)*
*Proportion women (M, SD)*		*0.75 (0.23)*	*0.71 (0.20)*	*0.74 (0.24)*	*0.09*	*0.74 (0.22)*
*Baseline severity (HAMD-17; M, SD)*		*17.04 (4.07)*	*17.57 (3.14)*	*17.27 (4.07)*	*0.31*	*17.22 (3.72)*

BAT: behavioural activation therapy; CAU: care-as-usual; CBT: cognitive behaviour therapy; IPT: interpersonal psychotherapy; M: means; SD: standard deviation; WL: waiting list.

a There were 3 studies in which both a WL and CAU control group was included; these studies were excluded from these analyses.

b This row includes the 3 studies with both a WL and CAU control group.

c At least one intervention arm includes this characteristic, which means that one trial can include more than one type of therapy or format.

d One of the cells has less than 5 studies, indicating that the p value may not be correct.

Most studies (79%) had two arms and the other 21% had three or more arms. Most studies included one depression outcome measure (73%), and 36% were rated as low risk of bias across all four domains. Most studies were conducted in Europe (38%), North America (30.0%) and East Asia (11%). The mean publication year was 2012 (SD = 9.75).

Most studies used a cut-off on a depression rating scale as inclusion criterion (56%), while the rest required a diagnosis according to a clinical interview for participation. Most studies were aimed at adults in general (37%), general medical patients (23%), women with perinatal depression (14%), older adults (8%) or college students (6%).

Participants were recruited through the community (37%), clinical referrals only (24%) or other sources (38%). The mean age across all samples was 43.21 (SD = 16.23), the mean proportion of women 0.74 (SD = 0.22) and the mean baseline severity score on the HDRS-17 was 17.22 (SD = 3.72).

### Differences between WL and CAU controlled trials

We found several significant differences between studies using a WL and CAU control group. Studies with WL examined less often IPT (*p* = 0.02), and more often a third wave therapy (*p* = 0.02). The WL studies examined less often an individual (*p* < 0.001) or telephone-delivered format (*p* = 0.02) and more often a guided self-help format (*p* < 0.001). Studies with WL were older (mean publication year: 2012) compared to studies with CAU (mean: 2014; *p* < 0.001), the target group differed significantly (more often aimed at adults in general and less often on perinatal depression; *p* < 0.001) and used different recruitment strategies (more often recruitment from the community; *p* < 0.001). WL controlled trials also had more often three or more treatment arms than trials with CAU (*p* < 0.001) and used only one depression outcome measure less often (*p* = 0.006).

### Effect sizes of treatment versus control in studies with CAU and WL

The overall effect size of all 472 comparisons between therapy and control (either CAU or WL) was *g* = 0.77 (95% CI: 0.71; 0.84) with high heterogeneity (*I*^2^ = 84; 95% CI: 82; 85; Table 2). This was somewhat smaller when outliers were excluded (*g* = 0.70), smaller when the analyses were limited to studies with low risk of bias (*g* = 0.62) and considerably smaller after adjustment for publication bias (*g* = 0.43).

**Table 2.** Effect sizes of treatment versus control, pre- to post within control conditions and pre- to post within therapy conditions across WL and CAU controlled trials

	All studies					CAU controlled studies					WL controlled studies					
	*k*	*g*	95% CI	*I*^2^	95% CI	*k*	*g*	95% CI	*I*^2^	95% CI	*k*	*g*	95% CI	*I*^2^	95% CI	*p*
Effect sizes for the difference between treatment and control at post-test*																
All studies	472	0.77	0.71; 0.84	84	82; 85	248	0.63	0.55; 0.71	85	83; 86	224	0.95	0.85; 1.04	80	78; 82	≤0.001*
Outliers excluded^a^	333	0.70	0.67; 0.73	21	9; 32	174	0.54	0.50; 0.58	25	9; 38	177	0.80	0.76; 0.85	19	2; 35	≤0.001*
Only low risk of bias	162	0.62	0.52; 0.72	84	81; 86	99	0.51	0.40; 0.61	78	73; 82	63	0.79	0.60; 0.97	88	85; 90	0.009*
Adj. for publ. bias^a^	619	0.43	0.35; 0.51	89	89; 90	316	0.34	0.23; 0.45	91	90; 91	296	0.58	0.46; 0.71	87	86; 88	0.004*
Pre-post effect sizes within control conditions*																
All studies	280	0.51	0.43; 0.60	95	94; 95	152	0.64	0.50; 0.78	96	96; 96	128	0.37	0.28; 0.46	91	89; 92	0.001*
Outliers excluded^a^	130	0.49	0.46; 0.53	52	42; 61	66	0.63	0.57; 0.69	68	58; 75	82	0.33	0.29; 0.38	48	33; 60	≤0.001*
Only low risk of bias	103	0.61	0.47; 0.74	95	94; 95	57	0.75	0.53; 0.98	96	95; 96	46	0.44	0.32; 0.56	93	91; 94	0.01*
Adj. for publ. bias^a^	322	0.32	0.21; 0.43	96	96; 96	181	0.36	0.18; 0.54	97	97; 97	149	0.24	0.13; 0.34	93	92; 93	0.25
Pre-post effect sizes within therapy conditions*																
All studies	407	1.48	1.40; 1.57	93	93; 94	200	1.44	1.30; 1.58	95	95; 96	207	1.52	1.41; 1.62	88	86; 89	0.38
Outliers excluded^a^	226	1.44	1.40; 1.48	34	23; 44	104	1.44	1.38; 1.50	50	38; 60	137	1.51	1.45; 1.57	40	26; 51	0.10
Only low risk of bias	139	1.36	1.22; 1.51	94	94; 95	77	1.34	1.15; 1.54	95	95; 96	62	1.38	1.18; 1.58	92	90; 93	0.81
Adj. for publ. bias^a^	583	0.89	0.77; 1.01	95	95; 95	285	0.79	0.60; 0.98	97	96; 97	295	1.02	0.87; 1.17	90	89; 91	0.06

95% CI: 95% confidence interval; adj: adjusted; CAU: care-as-usual; *g*: standardized mean difference (Hedges’ *g*); *k*: number of studies; publ. bias: publication bias; RoB: risk of bias; WL: waiting list.

a The effect sizes for the two subgroups were calculated separately for each subgroup, and the significance of the difference between the two subgroups were calculated afterward.

* *p*-value is significant.

Subgroup analyses indicated that WL controlled studies (*g* = 0.95; 95% CI: 0.85; 1.04; *I*^2^ = 80; 95% CI: 78; 82), had significantly larger effects than studies with CAU (*g* = 0.63; 95% CI: 0.55; 0.71; *I*^2^ = 85; 95% CI: 83; 86; *p* for difference < 0.001). This difference between WL and CAU controlled trials remained highly significant in all sensitivity analyses, including when outliers were excluded (*p* < 0.001), when limited to studies with low risk of bias (*p* = 0.009) and after adjustment for publication bias (*p* = 0.004).

We conducted a multivariable meta-regression with a dummy variable indicating whether a study used a CAU or WL control group as predictor, as well as all characteristics of the interventions, the patients and the studies that differed significantly between studies with a CAU and a WL control condition (Table 3). The dummy variable indicating type of control group remained significantly associated with the effect size (*p* < 0.001).

**Table 3.** Meta-regression analyses of effect sizes of treatment versus control, pre- to post within control conditions and pre- to post within therapy conditions

	Effect sizes (treatment vs control at post-test)			Pre-post effect size within control groups			Pre-post effect size within treatment groups		
Predictors	Coeff	SE	*p*	Coeff	SE	*p*	Coeff	SE	*p*
WL vs CAU	0.39	0.08	≤0.001*	−0.26	0.10	0.01*	0.20	0.11	0.06
IPT included	−0.26	0.13	0.05*	0.06	0.19	0.75	−0.18	0.19	0.33
3rd wave included	0.12	0.13	0.35	−0.12	0.14	0.41	0.00	0.16	0.99
Individual format included	−0.04	0.08	0.61	0.34	0.10	0.00*	0.28	0.10	0.01*
Telephone format included	0.06	0.16	0.73	0.28	0.22	0.21	0.30	0.23	0.18
Guided self-help included	−0.27	0.09	0.00*	0.28	0.11	0.02*	−0.06	0.12	0.59
Two arms vs more than two	−0.04	0.08	0.58	0.04	0.12	0.71	−0.05	0.11	0.64
One measure vs more	−0.12	0.07	0.08	−0.05	0.10	0.61	−0.16	0.09	0.09
Publication year	0.00	0.00	0.84	0.00	0.01	0.91	0.00	0.01	0.64
Target group: Adults in general	Ref.			Ref.			Ref.		
Target group: General medical	−0.14	0.11	0.19	−0.19	0.14	0.16	−0.42	0.15	0.01*
Target group: Older adults	−0.02	0.13	0.85	−0.31	0.17	0.07	−0.26	0.17	0.14
Target group: Perinatal depression	0.07	0.12	0.54	−0.06	0.14	0.66	−0.09	0.15	0.55
Target group: Students	0.01	0.13	0.94	0.25	0.16	0.13	0.28	0.18	0.12
Target group: Other	0.34	0.16	0.03*	−0.11	0.13	0.58	0.40	0.21	0.07
Recruitment: Clinical	Ref.			Ref.			Ref.		
Recruitment: Community	−0.04	0.10	0.68	−0.05	0.13	0.72	−0.13	0.14	0.35
Recruitment: Other	0.15	0.11	0.17	−0.23	0.14	0.09	0.03	0.15	0.83

CAU: care-as-usual; Coeff: coefficient; IPT: interpersonal psychotherapy; SE: standard error; WL: waiting list.

*p-value is significant.

### Pre-post effect sizes within control conditions

We could calculate pre-post effect sizes for the CAU and WL control conditions in 280 trials (152 CAU and 128 WL). The overall pre-post effect size across all studies was *g* = 0.51 (95% CI: 0.43; 0.60; *I*^2^ = 95; 95% CI: 94; 95). The pre-post effect size within the CAU conditions (*g* = 0.64; 95% CI: 0.50; 0.78; *I*^2^ = 96; 95% CI: 96; 96) was significantly larger than the one within the WL conditions (*g* = 0.37; 95% CI: 0.28; 0.46; *I*^2^ = 91; 95% CI: 89; 92; *p* for difference: 0.001). This difference remained significant after excluding outliers (*p* < 0.001) and after limiting the studies to those with low risk of bias (*p* = 0.01), but not after adjustment for publication bias (*p* = 0.25).

We conducted another multivariable meta-regression with the dummy variable for type of control group as well as all characteristics of the interventions, the patients and the studies that differed significantly between studies with a CAU and a WL control condition as predictors (Table 3). We found that the dummy variable indicating the type of control group remained significantly associated with the effect size (*p* = 0.01).

### Pre-post effect sizes within the therapy conditions

The overall pre-post effect size within the 407 therapy conditions was *g* = 1.48 (95% CI: 1.40; 1.57; *I*^2^ = 93; 95% CI: 93; 94; Table 2). We found no significant difference between the effect sizes of the trials with a CAU control group (*g* = 1.44; 95% CI: 1.30; 1.58; *I*^2^ = 95; 95% CI: 95; 96) and those with a WL control group (*g* = 1.52; 95% CI: 1.41; 1.62; *I*^2^ = 88; 95% CI: 86; 89; *p* for difference: 0.38). None of the sensitivity analyses indicated a significant difference between the two groups of studies (*p*’s from 0.06 to 0.81). The multivariable meta-regression with the pre-post effect size within the therapy conditions as the dependent variable, with the same predictors as the previous meta-regression analyses, also did not find that the dummy variable for type of control group was significant (*p* = 0.06; Table 3).

### Response rates

The overall response rate within control conditions was 0.17 (95% CI: 0.16; 0.19; *I*^2^ = 73; 95% CI: 69; 76; Appendix C). The response rate in the trials with a CAU control group (0.19; 95% CI: 0.17; 0.21; *I*^2^ = 78; 95% CI: 74; 81) was significantly larger than in the trials with a WL control group (0.16; 95% CI: 0.14; 0.18; *I*^2^ = 55; 95% CI: 45; 63; *p* for difference: <0.001). In the sensitivity analyses this higher response rate for CAU was confirmed (Appendix C).

The overall response rate within therapy conditions was 0.39 (95% CI: 0.37; 0.42; *I*^2^ = 83; 95% CI: 81; 84). There was no significant difference for the response rate in trials with a CAU control group (0.36; 95% CI: 033; 0.40; *I*^2^ = 88; 95% CI: 86; 89) and the trials with a WL control group (0.42; 95% CI: 0.39; 0.45; *I*^2^ = 71; 95% CI: 67; 75; *p* for difference: 0.27). The sensitivity analyses also supported these findings, except that in the studies with low risk of bias, the response rate was higher for trials with a CAU control group (*p* = 0.03).

We conducted a multivariable meta-regression analysis of the response rates within the control groups, in which we included a dummy variable indicating whether a study had a WL or CAU condition, as well as the other characteristics of the studies. This analysis supported the finding that trials with a WL control group resulted in lower response rates (Appendix D; *p* = 0.02). We also conducted a multivariable meta-regression analysis of the response rates within the treatment groups, with the same predictors (Appendix D). These analyses indicated that the rates were lower in the trials with a CAU control group compared with the WL controlled trials (*p* = 0.01). Because of the large number of tests and the relatively high *p*-value these results should be interpreted with caution.

## Discussion

We explored the difference between WL and CAU control conditions in randomized trials of psychological treatments of depression. We could confirm that WL controlled trials have larger effect sizes compared to trials with a CAU control group. This difference remained highly significant in all sensitivity analyses, as well as in a meta-regression analysis in which we adjusted for the differences in characteristics of the trials with WL and CAU control groups. This finding is in line with previous research showing that WL control groups substantially overestimate the effects of treatments, when compared to other control groups, such as CAU or placebo (Cuijpers *et al.*, 2016, 2021c; Furukawa *et al.*, 2014; Hesser *et al.*, 2011).

We also examined whether this difference between WL and CAU controlled trials was caused by a smaller change within the control group or a larger change within the treatment groups. We found that the change within the WL conditions was significantly smaller than the change within the CAU conditions. This remained significant in almost all sensitivity analyses, and also in the meta-regression analysis in which we adjusted for the differences in characteristics of the trials with WL and CAU conditions.

This finding suggests that the hypothesis is true that patients on WL actually ‘wait’ to change until they receive the intervention (Miller and Rollnick, 2002), while patients in the CAU conditions try to change their problems more actively. It is also possible that patients on WLs are less disappointed compared to patients assigned to CAU (Lindström *et al.*, 2010; Skingley *et al.*, 2014), because these patients will get the intervention, but only have to wait for some time.

We found no evidence that the patients in the treatment group improve more or less in the WL versus CAU controlled trials when they receive the treatment. Some sensitivity analyses did indicate differential effects, but these were not consistent and were probably the result of chance and the very high level of heterogeneity. This finding suggests that the difference between WL and CAU controlled trials is mostly caused by the difference in change within the control conditions.

We also found several significant differences in characteristics between the two groups of trials, including differences in type of therapy examined, treatment format, recency, target group, recruitment strategy, number of treatment arms and number of outcome measures. However, these differences could not explain the larger effect sizes of WL controlled trials.

It is difficult to understand the exact reasons for these differences, but it is important to conclude that such significant differences do exist, because this may point at different research questions that are examined in WL controlled trials. Recently, there have been attempts to develop models of between-comparator variability in psychological treatments that allow to re-estimate effects as if all interventions have been evaluated against the same standardized comparator (Glasziou and Zwar, 2023; Kraiss *et al.*, 2023). Such fine-grained approaches, albeit time-consuming to implement, could also be investigated in psychotherapy research to further illuminate the causes of effect differences between and within comparators.

Control conditions are an essential element in evidence-based mental health research. Unfortunately, there is no optimal control condition when examining the effects of psychological interventions (Cuijpers, 2024). All types of control groups, including CAU, psychological placebo, psychoeducation and WL have their own weaknesses and problems (Cuijpers, 2024; Harrer *et al.*, 2023). However, WL control groups should be avoided in randomized trials, because they clearly inflate the effect size by artificially reducing the change within the WL control group.

This study has several strengths. First, it is a meta-analysis of a large sample of trials. It is also the first to examine differences in characteristics of WL controlled trials with trials using another control group (CAU). To the best of our knowledge, this is also the first meta-analysis to examine change scores within control and treatment conditions in the context of this research question.

However, this study has also several important limitations that have to be taken into account when interpreting the outcomes. First, only 36% of the included studies was assessed as having low risk of bias. The main outcomes were confirmed when the analyses were limited to the studies with low risk of bias. However, the low quality of the full set of studies remains an important limitation of this meta-analysis. Second, we could only examine differences between the characteristics of trials using WL and CAU conditions that were available in our database. For example, we did not examine differences between the two groups of studies in terms of medication use. On the other hand, we found in a recent meta-analysis that the use of antidepressants is not related to the effects of psychotherapy (Cuijpers *et al.*, 2023d).

A third important limitation of our study is that heterogeneity was high to very high in most analyses, both for the WL and CAU control conditions. Although this is typically found when examining pre-post effect sizes and response rates, it still means that the effect sizes found in the included studies varied considerably and that we could not explain these differences through subgroup and meta-regression analyses. Future research should focus more on potential causes of this heterogeneity, as was recently done in an elegant meta-analysis in which different levels of intensity of CAU (Munder *et al.*, 2022). Such future research should focus on how the intensity of CAU can best be estimated and defined, but it should also take into consideration the setting in which CAU is delivered (primary, secondary, general medical, perinatal care; Cuijpers *et al.*, 2021c), and for example whether and how CAU was ‘enhanced’. Such more fine-grained research may also result in a better understanding of why effect sizes are overestimated in WL controlled trials compared to other control groups.

Despite these limitations, we can conclude that trials with WL control conditions considerably overestimate the effect sizes of psychological treatments, compared to trials using CAU control conditions, and that the overestimation of effect sizes is probably caused by a smaller improvement within the WL condition compared to the improvement in the CAU condition.

## Supporting information

Cuijpers et al. supplementary material Cuijpers et al. supplementary material

## Data Availability

Most data are available in the supplemental materials, all other information about the datasets and individual studies can be found at the website of the project www.metapsy.org. For questions and additional information, the first author can be contacted.

## Supplementary material

The supplementary material for this article can be found at https://doi.org/10.1017/S2045796024000611.
