# Supplementary material for: The overestimation of the effect sizes of psychotherapies for depression in waitlist controlled trials: a meta-analytic comparison with usual care controlled trials
Source: Epidemiol Psychiatr Sci. 2024 Nov 6;33:e56. doi: 10.1017/S2045796024000611 (PMC11561681; doi:10.1017/S2045796024000611)
Supplement: Cuijpers et al. supplementary material [file S2045796024000611sup001.docx]

# Supplemental materials

[Appendix A. References of included studies 2](#_Toc156469587)

[Appendix B. Selected characteristics of included studies 19](#_Toc156469588)

[Appendix C. Response rates 31](#_Toc156469589)

[Appendix D. Metaregression analyses of response rates 32](#_Toc156469590)

## Appendix A. References of included studies

| *Study* | *Reference* |
| --- | --- |
| Aagaard, 2017 | Aagaard, J., Foldager, L., Makki, A., Hansen, V., & M¸ller-Nielsen, K. (2017). The efficacy of psychoeducation on recurrent depression: a randomized trial with a 2-year follow-up. Nord J Psychiatry, 71(3), 223-229. |
| Abas, 2018 | Abas M, Nyamayaro P, Bere T, et al. Feasibility and acceptability of a task-shifted intervention to enhance adherence to HIV medication and improve depression in people living with HIV in Zimbabwe, a low income country in sub-Saharan Africa. AIDS and Behavior 2018; 22(1): 86-101. |
| Abbas, 2023 | Abbas, Q., Latif, S., Ayaz Habib, H., Shahzad, S., Sarwar, U., Shahzadi, M., . . . Washdev, W. (2023). Cognitive behavior therapy for diabetes distress, depression, health anxiety, quality of life and treatment adherence among patients with Type-II diabetes mellitus: A randomized control trial. BMC Psychiatry, 23. doi:10.1186/s12888-023-04546-w |
| Afonso, 2009 | Afonso, R., & Bueno, B. (2009). Efectos de un programa de reminiscencia sobre la sintomatología depresiva en una muestra de población mayor portuguesa. Revista Española de Geriatría y Gerontología, 44(6), 317-322. |
| Ahmadpanah, 2016 | Ahmadpanah, M., Paghale, S. J., Bakhtyari, A., Kaikhavani, S., Aghaei, E., Nazaribadie, M., . . . Brand, S. (2016). Effects of psychotherapy in combination with pharmacotherapy, when compared to pharmacotherapy only on blood pressure, depression, and anxiety in female patients with hypertension. |
| Alhusen, 2021 | Alhusen, J. L., Hayat, M. J., & Borg, L. (2021, Feb). A pilot study of a group-based perinatal depression intervention on reducing depressive symptoms and improving maternal-fetal attachment and maternal sensitivity. Arch Womens Ment Health, 24(1), 145-154. https://doi.org/10.1007/s00737-020-01032-0 |
| Allart van Dam, 2003 | Allart-Van Dam E, Hosman CMH, Hoogduin CAL, Schaap CPDR. The coping with depression course: Short-term outcomes and mediating effects of a randomized controlled trial in the treatment of subclinical depression. Behavior Therapy. 2003;34(3):381-96. |
| Amani, 2021 | Amani, B., Merza, D., Savoy, C., Streiner, D., Bieling, P., Ferro, M. A., & Van Lieshout, R. J. (2021, Nov 9). Peer-Delivered Cognitive-Behavioral Therapy for Postpartum Depression: A Randomized Controlled Trial. J Clin Psychiatry, 83(1). https://doi.org/10.4088/JCP.21m13928 |
| Araya, 2021 | Araya, R., Menezes, P. R., Claro, H. G., Brandt, L. R., Daley, K. L., Quayle, J., Diez-Canseco, F., Peters, T. J., Vera Cruz, D., Toyama, M., Aschar, S., Hidalgo-Padilla, L., Martins, H., Cavero, V., Rocha, T., Scotton, G., de Almeida Lopes, I. F., Begale, M., Mohr, D. C., & Miranda, J. J. (2021, May 11). Effect of a Digital Intervention on Depressive Symptoms in Patients With Comorbid Hypertension or Diabetes in Brazil and Peru: Two Randomized Clinical Trials. Jama, 325(18), 1852-1862. https://doi.org/10.1001/jama.2021.4348 |
| Arean, 1993 | Arean PA, Perri MG, Nezu AM, Schein RL, Christopher F, Joseph TX. Comparative effectiveness of social problem-solving therapy and reminiscence therapy as treatments for depression in older adults. Journal of Consulting and Clinical Psychology. 1993;61(6):1003-10. |
| Au, 2022 | Au, A., Nan, H., Sum, R., Ng, F., Kwong, A., & Wong, S. (2022). Cognitive behavioural therapy for adherence and sub-clinical depression in type 2 diabetes: a randomised controlled trial (abridged secondary publication). Hong Kong Med J, 28 Suppl 3(3), 21-23. |
| Ayen, 2004 | Ayen I, Hautzinger M. Cognitive behavior therapy for depression in menopausal women. A controlled, randomized treatment study. Zeitschrift fur Klinische Psychologie und Psychotherapie. 2004;33(4):290-9. |
| Barnhofer, 2009 | Barnhofer T, Crane C, Hargus E, Amarasinghe M, Winder R, Williams JM. Mindfulness-based cognitive therapy as a treatment for chronic depression: A preliminary study. Behaviour Research and Therapy. 2009;47(5):366-73. |
| Basirat, 2022 | Basirat, Z., Kheirkhah, F., Faramarzi, M., Esmaelzadeh, S., Khafri, S., & Tajali, Z. (2022). Pharmacotherapy or Psychotherapy? Selective Treatment Depression in The Infertile Women with Recurrent Pregnancy Loss: A Triple-Arm Randomized Controlled Trial. International Journal of Fertility and Sterility, 16(3), 211-219. doi:10.22074/ijfs.2021.529258.1124 |
| Baumgartner, 2021 | Baumeister, H., Paganini, S., Sander, L. B., Lin, J., Schlicker, S., Terhorst, Y., Moshagen, M., Bengel, J., Lehr, D., & Ebert, D. D. (2021). Effectiveness of a Guided Internet- and Mobile-Based Intervention for Patients with Chronic Back Pain and Depression (WARD-BP): A Multicenter, Pragmatic Randomized Controlled Trial. Psychother Psychosom, 90(4), 255-268. https://doi.org/10.1159/000511881 |
| Beach, 1992 | Beach SR, O'Leary KD. Treating depression in the context of marital discord: Outcome and predictors of response of marital therapy versus cognitive therapy. Behavior Therapy. 1992;23(4):507-28. |
| Bedard, 2014 | Bedard M, Felteau M, Marshall S, Cullen N, Gibbons C, Dubois S, et al. Mindfulness-based cognitive therapy reduces symptoms of depression in people with a traumatic brain injury: Results from a randomized controlled trial. Journal of Head Trauma Rehabilitation. 2014;29(4):E13-E22. |
| Beeber, 2010 | Beeber LS, Holditch-Davis D, Perreira K, Schwartz TA, Lewis V, Blanchard H, et al. Short-term in-home intervention reduces depressive symptoms in Early Head Start Latina mothers of infants and toddlers. Research in Nursing and Health. 2010;33(1):60-76. |
| Bendig, 2021 | Bendig, E., Bauereiß, N., Buntrock, C., Habibović, M., Ebert, D. D., & Baumeister, H. (2021). Lessons learned from an attempted randomized-controlled feasibility trial on “WIDeCAD” - An internet-based depression treatment for people living with coronary artery disease (CAD) [Article]. Internet Interventions, 24. https://doi.org/10.1016/j.invent.2021.100375 |
| Berger, 2011 | Berger T, Hämmerli K, Gubser N, Andersson G, Caspar F. Internet-based treatment of depression: A randomized controlled trial comparing guided with unguided self-help. Cognitive Behaviour Therapy. 2011;40(4):251-66. |
| Beutel, 2014 | Beutel ME, Weissflog G, Leuteritz K, Wiltink J, Haselbacher A, Ruckes C, et al. Efficacy of short-term psychodynamic psychotherapy (STPP) with depressed breast cancer patients: Results of a randomized controlled multicenter trial. Annals of Oncology. 2014;25(2):378-84. |
| Boele, 2018 | Boele FW, Klein M, Verdonck-de Leeuw IM, et al. Internet-based guided self-help for glioma patients with depressive symptoms: a randomized controlled trial. Journal of neuro-oncology 2018; 137(1): 191‐203. |
| Boeschoten, 2017 | Boeschoten, R. E., Dekker, J., Uitdehaag, B. M. J., Beekman, A. T. F., Hoogendoorn, A. W., Collette, E. H., . . . Van Oppen, P. (2017). Internet-based treatment for depression in multiple sclerosis: A randomized controlled trial. Multiple Sclerosis, 23(8), 1112-1122. |
| Bohlmeijer, 2011 | Bohlmeijer ET, Fledderus M, Rokx TA, Pieterse ME. Efficacy of an early intervention based on acceptance and commitment therapy for adults with depressive symptomatology: Evaluation in a randomized controlled trial. Behaviour research and therapy. 2011;49(1):62-7. |
| Bolton, 2003 | Bolton P, Bass J, Neugebauer R, Verdeli H, Clougherty KF, Wickramaratne P, et al. Group interpersonal psychotherapy for depression in rural Uganda: A randomized controlled trial. JAMA. 2003;289(23):3117-24. |
| Bower, 2021 | Bower, J. E., Partridge, A. H., Wolff, A. C., Thorner, E. D., Irwin, M. R., Joffe, H., Petersen, L., Crespi, C. M., & Ganz, P. A. (2021, Nov 1). Targeting Depressive Symptoms in Younger Breast Cancer Survivors: The Pathways to Wellness Randomized Controlled Trial of Mindfulness Meditation and Survivorship Education. J Clin Oncol, 39(31), 3473-3484. https://doi.org/10.1200/jco.21.00279 |
| Bowman, 1995 | Bowman D, Scogin F, Lyrene B. The efficacy of self-examination therapy and cognitive bibliotherapy in the treatment of mild to moderate depression. Psychotherapy Research. 1995;5(2):131-40. |
| Brown, 1984 | Brown RA, Lewinsohn PM. A psychoeducational approach to the treatment of depression: Comparison of group, individual, and minimal contact procedures. Journal of Consulting and Clinical Psychology. 1984;52(5):774. |
| Buhrman, 2015 | Buhrman M, Syk M, Burvall O, Hartig T, Gordh T, Andersson G. Individualized Guided Internet-delivered Cognitive Behaviour Therapy for Chronic Pain Patients with Comorbid Depression and Anxiety: A Randomized Controlled Trial. Clinical Journal of Pain 2014. |
| Buntrock, 2015 | Buntrock C, Ebert D, Lehr D, Riper H, Smit F, Cuijpers P, et al. Effectiveness of a web-based cognitive behavioural intervention for subthreshold depression: Pragmatic randomised controlled trial. Psychotherapy and Psychosomatics. 2015;84(6):348-58. |
| Burns, 2007 | Burns A, Banerjee S, Morris J, Woodward Y, Baldwin R, Proctor R, et al. Treatment and prevention of depression after surgery for hip fracture in older people: Randomized, controlled trials. Journal of the American Geriatrics Society. 2007;55(1):75-80. |
| Carlbring, 2013 | Carlbring P, Hagglund M, Luthstrom A, Dahlin M, Kadowaki A, Vernmark K, et al. Internet-based behavioral activation and acceptance-based treatment for depression: A randomized controlled trial. Journal of Affective Disorders. 2013;148(2-3):331-7. |
| Carta, 2012 | Carta MG, Petretto D, Adamo S, Bhat KM, Lecca ME, Mura G, et al. Counseling in primary care improves depression and quality of life. Clinical Practice and Epidemiology in Mental Health. 2012;8. |
| Casanas, 2012 | Casanas R, Catalan R, del Val JL, Real J, Valero S, Casas M. Effectiveness of a psycho-educational group program for major depression in primary care: A randomized controlled trial. BMC psychiatry. 2012;12:230. |
| Castonguay, 2004 | Castonguay LG, Schut AJ, Aikens DE, Constantino MJ, Laurenceau J-P, Bologh L, et al. Integrative cognitive therapy for depression: A preliminary investigation. Journal of Psychotherapy Integration. 2004;14(1):4-20. |
| Chan, 2012 | Chan AS, Wong QY, Sze SL, Kwong PP, Han YM, Cheung MC. A Chinese Chan-based mind-body intervention for patients with depression. Journal of Affective Disorders. 2012;142(1-3):283-9. |
| Chan, 2013 | Chan MF, Ng SE, Tien A, Man Ho RC, Thayala J. A randomised controlled study to explore the effect of life story review on depression in older Chinese in Singapore. Health and Social Care in the Community. 2013;21(5):545-53. |
| Chen, 2000 | Chen CH, Tseng YF, Chou FH, Wang SY. Effects of support group intervention in postnatally distressed women. A controlled study in Taiwan. Journal of psychosomatic research. 2000;49(6):395-9. |
| Chesney, 2003 | Chesney MA, Chambers DB, Taylor JM, Johnson LM, Folkman S. Coping effectiveness training for men living with HIV: Results from a randomized clinical trial testing a group-based intervention. Psychosomatic Medicine. 2003;65(6):1038-46. |
| Chiang, 2015 | Chiang KJ, Chen TH, Hsieh HT, Tsai JC, Ou KL, Chou KR. One-year follow-up of the effectiveness of cognitive behavioral group therapy for patients' depression: A randomized, single-blinded, controlled study. Scientific World Journal. 2015;2015:Article ID 373149. |
| Cho, 2008 | Cho HJ, Kwon JH, Lee JJ. Antenatal cognitive-behavioral therapy for prevention of postpartum depression: A pilot study. Yonsei medical journal. 2008;49(4):553-62. |
| Choi, 2012 | Choi I, Zou J, Titov N, Dear BF, Li S, Johnston L, et al. Culturally attuned Internet treatment for depression amongst Chinese Australians: A randomised controlled trial. Journal of Affective Disorders. 2012;136(3):459-68. |
| Chowdhary, 2016 | Chowdhary N, Anand A, Dimidjian S, Shinde S, Weobong B, Balaji M, et al. The Healthy Activity Program lay counsellor delivered treatment for severe depression in India: Systematic development and randomised evaluation. British Journal of Psychiatry. 2016;208(4):381-8. |
| Choy, 2016 | Choy JC, Lou VW. Effectiveness of the modified instrumental reminiscence intervention on psychological well-being among community-dwelling chinese older adults: A randomized controlled trial. American Journal of Geriatric Psychiatry. 2016;24(1):60-9. |
| Clark, 2003 | Clark R, Tluczek A, Wenzel A. Psychotherapy for postpartum depression: A preliminary report. American Journal of Orthopsychiatry. 2003;73(4):441-54. |
| Clark, 2008 | Clark R, Tluczek A, Brown R. A mother–infant therapy group model for postpartum depression. Infant Mental Health Journal. 2008;29(5):514-36. |
| Cohen, 2010 | Cohen S, O'Leary KD, Foran H. A randomized clinical trial of a brief, problem-focused couple therapy for depression. Behavior Therapy. 2010;41(4):433-46. |
| Cooper, 2003 | Cooper PJ, Murray L, Wilson A, Romaniuk H. Controlled trial of the short- and long-term effect of psychological treatment of post-partum depression. I. Impact on maternal mood. The British Journal of Psychiatry. 2003;182(5):412-9. |
| Cramer, 2011 | Cramer H, Salisbury C, Conrad J, Eldred J, Araya R. Group cognitive behavioural therapy for women with depression: Pilot and feasibility study for a randomised controlled trial using mixed methods. BMC psychiatry. 2011;11:82. |
| De Groot, 2019 | De Groot, M., Shubrook, J. H., Hornsby, W. G., Pillay, Y., Mather, K. J., Fitzpatrick, K., . . . Saha, C. (2019). Program ACTIVE II: Outcomes from a randomized, multistate community-based depression treatment for rural and urban adults with type 2 diabetes. Diabetes care, 42(7), 1185-1193. doi:10.2337/dc18-2400 |
| De Jong, 2018 | De Jong M, Peeters F, Gard T, et al. A randomized controlled pilot study on mindfulness-based cognitive therapy for unipolar depression in patients with chronic pain. Journal of clinical psychiatry 2018; 79(1): 26‐34. |
| Dekker, 2012 | Dekker RL, Moser DK, Peden AR, Lennie TA. Cognitive therapy improves three-month outcomes in hospitalized patients with heart failure. Journal of cardiac failure. 2012;18(1):10-20. |
| Demir, 2022 | Demir, S., & Ercan, F. (2022). The effectiveness of cognitive behavioral therapy-based group counseling on depressive symptomatology, anxiety levels, automatic thoughts, and coping ways Turkish nursing students: A randomized controlled trial. Perspectives in psychiatric care. doi:10.1111/ppc.13073 |
| Dennis, 2020 | Dennis CL, Grigoriadis S, Zupancic J, Kiss A, Ravitz P. Telephone-based nurse-delivered interpersonal psychotherapy for postpartum depression: nationwide randomised controlled trial. Br J Psychiatry. 2020;216(4):189-196. |
| Dimidjian, 2017 | Dimidjian, S., Goodman, S., Sherwood, N., Simon, G., Ludman, E., Gallop, R., . . . Beck, A. (2017). A Pragmatic Randomized Clinical Trial of Behavioral Activation for Depressed Pregnant Women. Journal of Consulting and Clinical Psychology, 85(1), 26-36 |
| Doering, 2013 | Doering LV, Chen B, Cross Bodan R, Magsarili MC, Nyamathi A, Irwin MR. Early cognitive behavioral therapy for depression after cardiac surgery. Journal of Cardiovascular Nursing. 2013;28(4):370-9. |
| Dong, 2019 | Dong, X., Sun, G., Zhan, J., Liu, F., Ma, S., Li, P., . . . Liu, Y. (2019). Telephone-based reminiscence therapy for colorectal cancer patients undergoing postoperative chemotherapy complicated with depression: a three-arm randomised controlled trial. Supportive care in cancer : official journal of the Multinational Association of Supportive Care in Cancer, 27(8), 2761-2769. doi:10.1007/s00520-018-4566-6 |
| Dowrick, 2000 | Dowrick, C., Dunn, G., Ayuso-Mateos, J. L., Dalgard, O. S., Page, H., Lehtinen, V., ... Wilkinson, G. (2000). Problem solving treatment and group psychoeducation for depression: multicentre randomised controlled trial Outcomes of Depression International Network [ODIN] Group. Bmj, 321(7274), 1450-1454. Retrieved from http://onlinelibrary.wiley.com/o/cochrane/clcentral/articles/836/CN-00623836/frame.html |
| Dwight-Johnson, 2011 | Dwight-Johnson M, Aisenberg E, Golinelli D, Hong S, O'Brien M, Ludman E. Telephone-based cognitive-behavioral therapy for Latino patients living in rural areas: A randomized pilot study. Psychiatric Services. 2011;62(8):936-42. |
| Ebert, 2014 | Ebert, D. D., Lehr, D., Boß, L., Riper, H., Cuijpers, P., Andersson, G., . . . Berking, M. (2014). Efficacy of an internet-based problem-solving training for teachers: results of a randomized controlled trial. Scandinavian journal of work, environment & health, 582-596. |
| Ede, 2020 | Ede MO, Igbo JN, Eseadi C, et al. Effect of group cognitive behavioural therapy on depressive symptoms in a sample of college adolescents in Nigeria. Journal of Rational-Emotive & Cognitive-Behavior Therapy. 2020;38(3):306-318. |
| Ekers, 2011 | Ekers D, Richards D, McMillan D, Bland JM, Gilbody S. Behavioural activation delivered by the non-specialist: Phase II randomised controlled trial. The British Journal of Psychiatry. 2011;198(1):66-72. |
| Embling, 2002 | Embling S. The effectiveness of cognitive behavioural therapy in depression. Nursing Standard. 2002;17(14-15):33-41. |
| Eseadi, 2018 | Eseadi C, Obidoa MA, Ogbuabor SE, Ikechukwu-Ilomuanya AB. Effects of Group-Focused Cognitive-Behavioral Coaching Program on Depressive Symptoms in a Sample of Inmates in a Nigerian Prison. International journal of offender therapy and comparative criminology 2018; 62(6): 1589-602. |
| Eseadi, 2022 | Eseadi, C., Ilechukwu, L. C., Victor-Aigbodion, V., Sewagegn, A. A., & Amedu, A. N. (2022). Intervention for depression among undergraduate religious education students: A randomized controlled trial. Medicine (Baltimore), 101(41), e31034. doi:10.1097/md.0000000000031034 |
| Euteneuer, 2017 | Euteneuer, F., Dannehl, K., Del Rey, A., Engler, H., Schedlowski, M., & Rief, W. (2017). Immunological effects of behavioral activation with exercise in major depression: An exploratory randomized controlled trial. Translational Psychiatry, 7(5). |
| Euteneuer, 2022 | Euteneuer, F., Neuert, M., Salzmann, S., Fischer, S., Ehlert, U., & Rief, W. (2022). Does psychological treatment of major depression reduce cardiac risk biomarkers? An exploratory randomized controlled trial. Psychological medicine, 1-15. doi:10.1017/S0033291722000447 |
| Ewais, 2021 | Ewais, T., et al. (2021). "Mindfulness based cognitive therapy for youth with inflammatory bowel disease and depression - Findings from a pilot randomised controlled trial." J Psychosom Res 149: 110594. |
| Fann, 2015 | Fann JR, Bombardier CH, Vannoy S, Dyer J, Ludman E, Dikmen S, et al. Telephone and in-person cognitive behavioral therapy for major depression after traumatic brain injury: A randomized controlled trial. Journal of neurotrauma. 2015;32(1):45-57. |
| Faramarzi, 2008 | Faramarzi M, Alipor A, Esmaelzadeh S, Kheirkhah F, Poladi K, Pash H. Treatment of depression and anxiety in infertile women: Cognitive behavioral therapy versus fluoxetine. Journal of Affective Disorders. 2008;108(1-2):159-64. |
| Fledderus, 2012 | Fledderus M, Bohlmeijer ET, Pieterse ME, Schreurs KM. Acceptance and commitment therapy as guided self-help for psychological distress and positive mental health: A randomized controlled trial. Psychological Medicine. 2012;42(3):485-95. |
| Floyd, 2004 | Floyd M, Scogin F, McKendree-Smith NL, Floyd DL, Rokke PD. Cognitive therapy for depression: A comparison of individual psychotherapy and bibliotherapy for depressed older adults. Behavior modification. 2004;28(2):297-318. |
| Folke, 2012 | Folke F, Parling T, Melin L. Acceptance and commitment therapy for depression: A preliminary randomized clinical trial for unemployed on long-term sick leave. Cognitive and Behavioral Practice. 2012;19(4):583-94. |
| Frangou, 2021 | Frangou, E., Bertelli, G., Love, S., Mackean, M. J., Glasspool, R. M., Fotopoulou, C., Cook, A., Nicum, S., Lord, R., Ferguson, M., Roux, R. L., Martinez, M., Butcher, C., Hulbert-Williams, N., Howells, L., & Blagden, S. P. (2021). OVPSYCH2: A randomized controlled trial of psychological support versus standard of care following chemotherapy for ovarian cancer. Gynecol Oncol, 162(2), 431-439. https://doi.org/10.1016/j.ygyno.2021.05.024 |
| Freedland, 2009 | Freedland KE, Skala JA, Carney RM, Rubin EH, Lustman PJ, D·vila-Rom·n VG, et al. Treatment of depression after coronary artery bypass surgery: A randomized controlled trial. Archives of General Psychiatry. 2009;66(4):387-96. |
| Fuhr, 2019 | Fuhr, D. C., Weobong, B., Lazarus, A., Vanobberghen, F., Weiss, H. A., Singla, D. R., . . . Patel, V. (2019). Delivering the Thinking Healthy Programme for perinatal depression through peers: An individually randomised controlled trial in India. The Lancet Psychiatry, 6(2), 115-127. doi:10.1016/S2215-0366(18)30466-8 |
| Funderburk, 2021 | Funderburk, J. S., Pigeon, W. R., Shepardson, R. L., Wade, M., Acker, J., Fivecoat, H., Wray, L. O., & Maisto, S. A. (2021). Treating depressive symptoms among veterans in primary care: A multi-site RCT of brief behavioral activation. J Affect Disord, 283, 11-19. https://doi.org/10.1016/j.jad.2021.01.033 |
| Furukawa, 2012 | Furukawa TA, Horikoshi M, Kawakami N, Kadota M, Sasaki M, Sekiya Y, et al. Telephone cognitive-behavioral therapy for subthreshold depression and presenteeism in workplace: A randomized controlled trial. PLoS One. 2012;7(4):e35330. |
| Garcia-Pena, 2015 | García-Peña C, Vázquez-Estupiñan F, Avalos-Pérez F, Jiménez LVR, Sánchez-Garcia S, Juárez-Cedillo T. Clinical effectiveness of group cognitive-behavioural therapy for depressed older people in primary care: A randomised controlled trial. Salud Mental. 2015;38(1):33-9. |
| Gawrysiak, 2009 | Gawrysiak M, Nicholas C, Hopko DR. Behavioral activation for moderately depressed university students: Randomized controlled trial. Journal of Counseling Psychology. 2009;56(3):468-75. |
| Gellis, 2008 | Gellis ZD, McGinty J, Tierney L, Jordan C, Burton J, Misener E. Randomized controlled trial of problem-solving therapy for minor depression in home care. Research on Social Work Practice. 2008;18(6):596-606. |
| Gellis, 2010 | Gellis ZD, Bruce ML. Problem solving therapy for subthreshold depression in home healthcare patients with cardiovascular disease. The American Journal of Geriatric Psychiatry. 2010;18(6):464-74. |
| Geraedts, 2014 | Geraedts AS, Kleiboer AM, Wiezer NM, van Mechelen W, Cuijpers P. Short-term effects of a web-based guided self-help intervention for employees with depressive symptoms: Randomized controlled trial. Journal of Medical Internet Research. 2014;16(5):e121. |
| Ghorbani, 2021 | Ghorbani, V., Zanjani, Z., Omidi, A., & Sarvizadeh, M. (2021). Efficacy of acceptance and commitment therapy (ACT) on depression, pain acceptance, and psychological flexibility in married women with breast cancer: a pre- and post-test clinical trial. Trends Psychiatry Psychother, 43(2), 126-133. https://doi.org/10.47626/2237-6089-2020-0022 |
| Gibbons, 2012 | Gibbons MB, Thompson SM, Scott K, Schauble LA, Mooney T, Thompson D, et al. Supportive-expressive dynamic psychotherapy in the community mental health system: A pilot effectiveness trial for the treatment of depression. Psychotherapy (Chicago, Ill). 2012;49(3):303-16. |
| Goodman, 2015 | Goodman JH, Prager J, Goldstein R, Freeman M. Perinatal Dyadic Psychotherapy for postpartum depression: A randomized controlled pilot trial. Archives of Women's Mental Health. 2015;18(3):493-506. |
| Gureje, 2022 | Gureje, O., Oladeji, B. D., Kola, L., Bello, T., Ayinde, O., Faregh, N., . . . Zelkowitz, P. (2022). Effect of intervention delivered by frontline maternal care providers to improve outcome and parenting skills among adolescents with perinatal depression in Nigeria (the RAPiD study): A cluster randomized controlled trial. J Affect Disord, 312, 169-176. doi:10.1016/j.jad.2022.06.032 |
| Hagen, 2017 | Hagen, R., Hjemdal, O., Solem, S., Kennair, L. E. O., Nordahl, H. M., Fisher, P., & Wells, A. (2017). Metacognitive therapy for depression in adults: A waiting list randomized controlled trial with six months follow-up. Frontiers in Psychology, 8. |
| Hallford, 2016 | Hallford DJ, Mellor D. Autobiographical memory-based intervention for depressive symptoms in young adults: A randomized controlled trial of cognitive-reminiscence therapy. Psychotherapy and Psychosomatics. 2016;85(4):246-9. |
| Hallgren, 2015 | Hallgren M, Kraepelien M, Öjehagen A, Lindefors N, Zeebari Z, Kaldo V, et al. Physical exercise and internet-based cognitive-behavioural therapy in the treatment of depression: Randomised controlled trial. British Journal of Psychiatry. 2015;207(3):227-34. |
| Hamamci, 2006 | Hamamci Z. Integrating psychodrama and cognitive behavioral therapy to treat moderate depression. Arts in Psychotherapy. 2006;33(3):199-207. |
| Hamdan-Mansour, 2009 | Hamdan-Mansour AM, Puskar K, Bandak AG. Effectiveness of cognitive-behavioral therapy on depressive symptomatology, stress and coping strategies among Jordanian university students. Issues in mental health nursing. 2009;30(3):188-96. |
| Han, 2020 | Han YMY, Sze SL, Wong QY, Chan AS. A mind-body lifestyle intervention enhances emotional control in patients with major depressive disorder: a randomized, controlled study. Cognitive, affective & behavioral neuroscience. 2020;20(5):1056-1069. |
| Haringsma, 2006 | Haringsma R, Engels G, Cuijpers P, Spinhoven P. Effectiveness of the Coping With Depression (CWD) course for older adults provided by the community-based mental health care system in the Netherlands: A randomized controlled field trial. International Psychogeriatrics. 2006;18(02):307-25. |
| Hashemi, 2022 | Hashemi, Z., Eyni, S., & Ebadi, M. (2022). Effectiveness of Acceptance and Commitment Therapy in Depression and Anxiety in People with Substance Use Disorder. Iranian Journal of Psychiatry and Behavioral Sciences, 16(1). doi:10.5812/ijpbs.110135 |
| Hassiotis, 2013 | Hassiotis A, Serfaty M, Azam K, Strydom A, Blizard R, Romeo R, et al. Manualised individual cognitive behavioural therapy for mood disorders in people with mild to moderate intellectual disability: A feasibility randomised controlled trial. Journal of Affective Disorders. 2013;151(1):186-95. |
| Hayman, 1980 | Hayman PM, Cope CS. Effects of assertion training on depression. Journal of clinical psychology. 1980;36(2):534-43. |
| Heckman, 2013 | Heckman, T. G., Heckman, B. D., Anderson, T., Lovejoy, T. I., Mohr, D., Sutton, M., . . . Gau, J.-T. (2013). Supportive-expressive and coping group teletherapies for HIV-infected older adults: a randomized clinical trial. AIDS Behav, 17(9), 3034-3044. |
| Heckman, 2017 | Heckman, T. G., Heckman, B. D., Anderson, T., Lovejoy, T. I., Markowitz, J. C., Shen, Y., & Sutton, M. (2017). Tele-interpersonal psychotherapy acutely reduces depressive symptoms in depressed HIV-infected rural persons: A randomized clinical trial. Behavioral Medicine, 43(4), 285-295. |
| Hemanny, 2019 | Hemanny, C., Carvalho, C., Maia, N., Reis, D., Botelho, A. C., Bonavides, D., . . . De Oliveira, I. R. (2019). Efficacy of trial-based cognitive therapy, behavioral activation and treatment as usual in the treatment of major depressive disorder: Preliminary findings from a randomized clinical trial. CNS Spectrums. doi:10.1017/S1092852919001457 |
| Herrmann-Lingen, 2016 | Herrmann-Lingen C, Beutel ME, Bosbach A, Deter HC, Fritzsche K, Hellmich M, et al. A stepwise psychotherapy intervention for reducing risk in coronary artery disease (SPIRR-CAD): Results of an observer-blinded, multicenter, randomized trial in depressed patients with coronary artery disease. Psychosomatic Medicine. 2016;78(6):704-15. |
| Holden, 1989 | Holden JM, Sagovsky R, Cox JL. Counselling in a general practice setting: Controlled study of heath visitor intervention in treatment of postnatal depression. British Medical Journal. 1989;298(6668):223-6. |
| Honey, 2002 | Honey KL, Bennett P, Morgan M. A brief psycho‐educational group intervention for postnatal depression. British Journal of Clinical Psychology. 2002;41(4):405-9. |
| Horrell, 2014 | Horrell L, Goldsmith KA, Tylee AT, Schmidt UH, Murphy CL, Bonin E-M, et al. One-day cognitive–behavioural therapy self-confidence workshops for people with depression: Randomised controlled trial. The British Journal of Psychiatry. 2014;204(3):222-33. |
| Hou, 2014 | Hou Y, Hu P, Zhang Y, Lu Q, Wang D, Yin L, et al. Cognitive behavioral therapy in combination with systemic family therapy improves mild to moderate postpartum depression. Revista Brasileira de Psiquiatria. 2014;36(1):47-52. |
| Hsiao, 2014 | Hsiao, F.-H., Lai, Y.-M., Chen, Y.-T., Yang, T.-T., Liao, S.-C., Ho, R. T., Ng, S.-M., Chan, C. L., & Jow, G.-M. (2014). Efficacy of psychotherapy on diurnal cortisol patterns and suicidal ideation in adjustment disorder with depressed mood. General hospital psychiatry, 36(2), 214-219. http://ac.els-cdn.com/S0163834313003113/1-s2.0-S0163834313003113-main.pdf?_tid=685fbc62-cf4e-11e5-b7e9-00000aab0f6c&acdnat=1455037317_a8c762fea010c7ac31f2152769dad94c |
| Huang, 2016 | Huang, C.-Y., Lai, H.-L., Chen, C.-I., Lu, Y.-C., Li, S.-C., Wang, L.-W., & Su, Y. (2016). Effects of motivational enhancement therapy plus cognitive behaviour therapy on depressive symptoms and health-related quality of life in adults with type II diabetes mellitus: A randomised controlled trial. Quality of Life Research: An International Journal of Quality of Life Aspects of Treatment, Care & Rehabilitation, 25(5), 1275-1283. |
| Hum, 2019 | Hum, K. M., Chan, C. J., Gane, J., Conway, L., McAndrews, M. P., & Smith, M. L. (2019). Do distance-delivery group interventions improve depression in people with epilepsy? Epilepsy & Behavior, 98, 153‐160. doi:10.1016/j.yebeh.2019.06.037 |
| Hummel, 2017 | Hummel, J., Weisbrod, C., Boesch, L., Himpler, K., Hauer, K., Hautzinger, M., . . . Kopf, D. (2017). AIDE–Acute Illness and Depression in Elderly Patients. Cognitive Behavioral Group Psychotherapy in Geriatric Patients With Comorbid Depression: A Randomized, Controlled Trial. Journal of the american medical directors association, 18(4), 341-349. |
| Hunter, 2012 | Hunter SB, Watkins KE, Hepner KA, Paddock SM, Ewing BA, Osilla KC, et al. Treating depression and substance use: A randomized controlled trial. Journal of substance abuse treatment. 2012;43(2):137-51. |
| Husain, 2017 | Husain, N., Zulqernain, F., Carter, L.-A., Chaudhry, I., Fatima, B., Kiran, T., . . . Rahman, A. (2017). Treatment of maternal depression in urban slums of Karachi, Pakistan: a randomized controlled trial (RCT) of an integrated maternal psychological and early child development intervention. Asian journal of psychiatry, 29, 63-70. |
| Husain, 2021a | Husain, N., Kiran, T., Fatima, B., Chaudhry, I. B., Husain, M., Shah, S., Bassett, P., Cohen, N., Jafri, F., Naeem, S., Zadeh, Z., Roberts, C., Rahman, A., Naeem, F., Husain, M. I., & Chaudhry, N. (2021). An integrated parenting intervention for maternal depression and child development in a low-resource setting: Cluster randomized controlled trial [Article]. Depress Anxiety, 38(9), 925-939. https://doi.org/10.1002/da.23169 |
| Husain, 2021b | Husain, N., Kiran, T., Shah, S., Rahman, A., Raza Ur, R., Saeed, Q., Naeem, S., Bassett, P., Husain, M., Haq, S. U., Jaffery, F., Cohen, N., Naeem, F., & Chaudhry, N. (2021). Efficacy of learning through play plus intervention to reduce maternal depression in women with malnourished children: A randomized controlled trial from Pakistan(✰). J Affect Disord, 278, 78-84. https://doi.org/10.1016/j.jad.2020.09.001 |
| Jalali, 2019 | Jalali, F., Hasani, A., Hashemi, S. F., Kimiaei, S. A., & Babaei, A. (2019). Cognitive Group Therapy Based on Schema-Focused Approach for Reducing Depression in Prisoners Living With HIV. International journal of offender therapy and comparative criminology, 63(2), 276‐288. doi:10.1177/0306624X18784185 |
| Jesse, 2015 | Jesse DE, Gaynes BN, Feldhousen EB, Newton ER, Bunch S, Hollon SD. Performance of a culturally tailored cognitive-behavioral intervention integrated in a public health setting to reduce risk of antepartum depression: A randomized controlled trial. Journal of Midwifery and Women's Health. 2015;60(5):578-92. |
| Jiang, 2014 | Jiang L, Wang ZZ, Qiu LR, Wan GB, Lin Y, Wei Z. Psychological intervention for postpartum depression. Journal of Huazhong University of Science and Technology: Medical sciences. 2014;34(3):437-42. |
| Johansson, 2012a | Johansson R, Ekbladh S, Hebert A, Lindström M, Möller S, Petitt E, et al. Psychodynamic guided self-help for adult depression through the internet: A randomised controlled trial. PloS One. 2012;7(5):e38021. |
| Johansson, 2012b | Johansson R, Sjöberg E, Sjögren M, Johnsson E, Carlbring P, Andersson T, et al. Tailored vs. standardized internet-based cognitive behavior therapy for depression and comorbid symptoms: A randomized controlled trial. PloS One. 2012;7(5):e36905. |
| Joling, 2011 | Joling KJ, Hout HP, van't Veer-Tazelaar PJ, Horst HE, Cuijpers P, Ven PM, et al. How effective is bibliotherapy for very old adults with subthreshold depression? A randomized controlled trial. American Journal of Geriatric Psychiatry. 2011;19(3):256-65. |
| Jordans, 2019 | Jordans, M. J. D., Luitel, N. P., Garman, E., Kohrt, B. A., Rathod, S. D., Shrestha, P., . . . Patel, V. (2019). Effectiveness of psychological treatments for depression and alcohol use disorder delivered by community-based counsellors: Two pragmatic randomised controlled trials within primary healthcare in Nepal. British Journal of Psychiatry, 215(2), 485-493. doi:10.1192/bjp.2018.300 |
| Kamga, 2017 | Kamga, H., McCusker, J., Yaffe, M., Sewitch, M., Sussman, T., Strumpf, E., . . . Freeman, E. (2017). Self-care tools to treat depressive symptoms in patients with age-related eye disease: a randomized controlled clinical trial. Clinical & experimental ophthalmology, 45(4), 371-378. |
| Kanter, 2015 | Kanter JW, Santiago-Rivera AL, Santos MM, Nagy G, López M, Hurtado GD, et al. A randomized hybrid efficacy and effectiveness trial of behavioral activation for latinos with depression. Behavior Therapy. 2015;46(2):177-92. |
| Keeley, 2016 | Keeley RD, Brody DS, Engel M, Burke BL, Nordstrom K, Moralez E, et al. Motivational interviewing improves depression outcome in primary care: A cluster randomized trial. Journal of Consulting and Clinical Psychology. 2016;84(11):993-1007. |
| Kemmeren, 2023 | Kemmeren, L. L., van Schaik, A., Draisma, S., Kleiboer, A., Riper, H., & Smit, J. H. (2023). Effectiveness of Blended Cognitive Behavioral Therapy Versus Treatment as Usual for Depression in Routine Specialized Mental Healthcare: E-COMPARED Trial in the Netherlands. Cognitive Therapy and Research. doi:10.1007/s10608-023-10363-y |
| Kenter, 2016 | Kenter, R. M. F., Cuijpers, P., Beekman, A., & van Straten, A. (2016). Effectiveness of a Web-based guided self-help intervention for outpatients with a depressive disorder: Short-term results from a randomized controlled trial. Journal of medical Internet research, 18(3). |
| Kessler, 2009 | Kessler D, Lewis G, Kaur S, Wiles N, King M, Weich S, et al. Therapist-delivered Internet psychotherapy for depression in primary care: A randomised controlled trial. Lancet. 2009;374(9690):628-34. |
| Khazraee, 2023 | Khazraee, H., Bakhtiari, M., Kianimoghadam, A. S., & Ghorbanikhah, E. (2023). The Effectiveness of Mindful Hypnotherapy on Depression, Self-Compassion, and Psychological Inflexibility in Females with Major Depressive Disorder: A Single-Blind, Randomized Clinical Trial. Int J Clin Exp Hypn, 71(1), 63-78. doi:10.1080/00207144.2022.2160257 |
| Khoshbooii, 2021 | Khoshbooii, R., Hassan, S. A., Deylami, N., Muhamad, R., Engku Kamarudin, E. M., & Alareqe, N. A. (2021). Effects of Group and Individual Culturally Adapted Cognitive Behavioral Therapy on Depression and Sexual Satisfaction among Perimenopausal Women. Int J Environ Res Public Health, 18(14). https://doi.org/10.3390/ijerph18147711 |
| Kim, 2018 | Kim YH, Choi KS, Han K, Kim HW. A psychological intervention programme for patients with breast cancer under chemotherapy and at a high risk of depression: a randomised clinical trial. Journal of clinical nursing 2018; 27(3‐4): 572‐81. |
| King, 2000 | King M, Sibbald B, Ward E, Bower P, Lloyd M, Gabbay M, et al. Randomised controlled trial of non-directive counselling, cognitive-behaviour therapy and usual general practitioner care in the management of depression as well as mixed anxiety and depression in primary care. Health Technology Assessment. 2000;4(19):1-83. |
| Kivi, 2014 | Kivi M, Eriksson MCM, Hange D, Petersson E-L, Vernmark K, Johansson B, et al. Internet-based therapy for mild to moderate depression in Swedish primary care: Short term results from the PRIM-NET randomized controlled trial. Cognitive Behaviour Therapy. 2014;43(4):289-98. |
| Korrelboom, 2012 | Korrelboom K, Maarsingh M, Huijbrechts I. Competitive memory training (COMET) for treating low self-esteem in patients with depressive disorders: A randomized clinical trial. Depression and anxiety. 2012;29(2):102-10. |
| Korte, 2012 | Korte J, Bohlmeijer ET, Cappeliez P, Smit F, Westerhof GJ. Life review therapy for older adults with moderate depressive symptomatology: A pragmatic randomized controlled trial. Psychological Medicine. 2012;42(6):1163-73. |
| Kramer, 2014 | Kramer, J., Conijn, B., Oijevaar, P., & Riper, H. (2014). Effectiveness of a web-based solution-focused brief chat treatment for depressed adolescents and young adults: randomized controlled trial. J Med Internet Res, 16(5), e141. doi:10.2196/jmir.3261 |
| Kramer, 2021 | Krämer, L. V., Grünzig, S. D., Baumeister, H., Ebert, D. D., & Bengel, J. (2021). Effectiveness of a Guided Web-Based Intervention to Reduce Depressive Symptoms before Outpatient Psychotherapy: A Pragmatic Randomized Controlled Trial. Psychother Psychosom, 90(4), 233-242. https://doi.org/10.1159/000515625 |
| Laidlaw, 2008 | Laidlaw K, Davidson K, Toner H, Jackson G, Clark S, Law J, et al. A randomised controlled trial of cognitive behaviour therapy vs treatment as usual in the treatment of mild to moderate late life depression. International Journal of Geriatric Psychiatry. 2008;23(8):843-50. |
| Lamers, 2010 | Lamers F, Jonkers CC, Bosma H, Kempen GI, Meijer JA, Penninx BW, et al. A minimal psychological intervention in chronically ill elderly patients with depression: A randomized trial. Psychotherapy and Psychosomatics. 2010;79(4):217-26. |
| Lamers, 2015 | Lamers SMA, Bohlmeijer ET, Korte J, Westerhof GJ. The efficacy of life-review as online-guided self-help for adults: A randomized trial. The Journals of Gerontology: Series B: Psychological Sciences and Social Sciences 2015; 70B(1): 24-34. |
| Landreville, 1997 | Landreville P, Bissonnette L. Effects of cognitive bibliotherapy for depressed older adults with a disability. Clinical Gerontologist. 1997;17(4):35-55. |
| Lappalainen, 2015 | Lappalainen P, Langrial S, Oinas-Kukkonen H, Tolvanen A, Lappalainen R. Web-based acceptance and commitment therapy for depressive symptoms with minimal support: A randomized controlled trial. Behavior modification. 2015;39(6):805-34. |
| Larcombe, 1984 | Larcombe NA, Wilson PH. An evaluation of cognitive-behaviour therapy for depression in patients with multiple sclerosis. The British Journal of Psychiatry. 1984;145:366-71. |
| Lenze, 2017 | Lenze, S., & Potts, M. (2017). Brief Interpersonal Psychotherapy for depression during pregnancy in a low-income population: a randomized controlled trial. Journal of Affective Disorders, 210, 151-157. |
| Lenze, 2020 | Lenze SN, Potts MA, Rodgers J, Luby J. Lessons learned from a pilot randomized controlled trial of dyadic interpersonal psychotherapy for perinatal depression in a low-income population. Journal of Affective Disorders. 2020;271:286-292. |
| Lerner, 2015 | Lerner D, Adler DA, Rogers WH, Chang H, Greenhill A, Cymerman E, et al. A randomized clinical trial of a telephone depression intervention to reduce employee presenteeism and absenteeism. Psychiatric Services. 2015;66(6):570-7. |
| Leung, 2013 | Leung SS, Lee AM, Chiang VC, Lam SK, Kuen YW, Wong DF. Culturally sensitive, preventive antenatal group cognitive-behavioural therapy for Chinese women with depression. Internationan Journal of Nursing Practice. 2013;19(Supp 1):28-37. |
| Lexis, 2011 | Lexis MA, Jansen NW, Huibers MJ, Amelsvoort LG, Berkouwer A, Tjin ATG, et al. Prevention of long-term sickness absence and major depression in high-risk employees: A randomised controlled trial. Occupational and Environmental Medicine. 2011;68(6):400-7. |
| Liu, 2009 | Liu ET-H, Chen W-L, Li Y-H, Wang CH, Mok TJ, Huang HS. Exploring the efficacy of cognitive bibliotherapy and a potential mechanism of change in the treatment of depressive symptoms among the Chinese: A randomized controlled trial. Cognitive Therapy and Research. 2009;33(5):449-61. |
| Liu, 2021 | Liu, H., & Yang, Y. (2021). Effects of a psychological nursing intervention on prevention of anxiety and depression in the postpartum period: a randomized controlled trial [Article]. Annals of General Psychiatry, 20(1). https://doi.org/10.1186/s12991-020-00320-4 |
| Lloyd-Williams, 2018 | Lloyd-Williams M, Shiels C, Ellis J, et al. Pilot randomised controlled trial of focused narrative intervention for moderate to severe depression in palliative care patients: DISCERN trial. Palliative medicine 2018; 32(1): 206‐15. |
| Lök, 2019 | Lök, N., Bademli, K., & Selçuk-Tosun, A. (2019). The effect of reminiscence therapy on cognitive functions, depression, and quality of life in Alzheimer patients: randomized controlled trial. International Journal of Geriatric Psychiatry, 34(1), 47‐53. doi:10.1002/gps.4980 |
| Lovell, 2008 | Lovell K, Bower P, Richards D, Barkham Mi, Sibbald B, Roberts C, et al. Developing guided self-help for depression using the medical research council complex interventions framework: A description of the modelling phase and results of an exploratory randomised controlled trial. BMC psychiatry. 2008;8. |
| Lund, 2020 | Lund, C., Schneider, M., Garman, E. C., Davies, T., Munodawafa, M., Honikman, S., . . . Susser, E. (2019). Task-sharing of psychological treatment for antenatal depression in Khayelitsha, South Africa: Effects on antenatal and postnatal outcomes in an individual randomised controlled trial. Behaviour Research and. doi:10.1016/j.brat.2019.103466 |
| Lundgren, 2016 | Lundgren JG, Dahlstrom O, Andersson G, Jaarsma T, Karner Kohler A, Johansson P. The effect of guided web-based cognitive behavioral therapy on patients with depressive symptoms and heart failure: A pilot randomized controlled trial. Journal of Medical Internet Research. 2016;18(8):e194. |
| Lustman, 1998 | Lustman PJ, Griffith LS, Freedland KE, Kissel SS, Clouse RE. Cognitive behavior therapy for depression in type 2 diabetes mellitus. A randomized, controlled trial. Annals of Internal Medicine. 1998;129(8):613-21. |
| Lynch, 1997 | Lynch DJ, Tamburrino MB, Nagel R. Telephone counseling for patients with minor depression: Preliminary findings in a family practice setting. The Journal of family practice. 1997;44(3):293-8. |
| Lynch, 2004 | Lynch D, Tamburrino M, Nagel R, Smith MK. Telephone-based treatment for family practice patients with mild depression. Psychological reports. 2004;94(3 Pt 1):785-92. |
| Lynch, 2020 | Lynch, T. R., Hempel, R. J., Whalley, B., Byford, S., Chamba, R., Clarke, P., . . . Russell, I. T. (2019). Refractory depression - mechanisms and efficacy of radically open dialectical behaviour therapy (RefraMED): findings of a randomised trial on benefits and harms. The British journal of psychiatry : the journal of mental science, 1-9. doi:10.1192/bjp.2019.53 |
| Mahmoodi, 2021 | Mahmoodi, M., Bakhtiyari, M., Masjedi Arani, A., Mohammadi, A., & Saberi Isfeedvajani, M. (2021). The comparison between CBT focused on perfectionism and CBT focused on emotion regulation for individuals with depression and anxiety disorders and dysfunctional perfectionism: A randomized controlled trial. Behavioural and Cognitive Psychotherapy, 49(4), 454-471. https://doi.org/10.1017/S1352465820000909 |
| Maina, 2005 | Maina G, Forner F, Bogetto F. Randomized controlled trial comparing brief dynamic and supportive therapy with waiting list condition in minor depressive disorders. Psychotherapy and Psychosomatics. 2005;74(1):43-50. |
| Malouff, 1988 | Malouff JM, Lanyon RI, Schutte NS. Effectiveness of a brief group RET treatment for divorce-related dysphoria. Journal of Rational-Emotive and Cognitive-Behavior Therapy. 1988;6(3):162-71. |
| Martin, 2015 | Martin PR, Aiello R, Gilson K, Meadows G, Milgrom J, Reece J. Cognitive behavior therapy for comorbid migraine and/or tension-type headache and major depressive disorder: An exploratory randomized controlled trial. Behaviour Research and Therapy. 2015;73:8-18. |
| McClay, 2015 | McClay CA, Collins K, Matthews L, Haig C, McConnachie A, Morrison J, et al. A community-based pilot randomised controlled study of life skills classes for individuals with low mood and depression. BMC psychiatry. 2015;15:17. |
| McCusker, 2021 | McCusker, J., Jones, J. M., Li, M., Faria, R., Yaffe, M. J., Lambert, S. D., Ciampi, A., Belzile, E., & de Raad, M. (2021). CanDirect: Effectiveness of a Telephone-Supported Depression Self-Care Intervention for Cancer Survivors. J Clin Oncol, 39(10), 1150-1161. https://doi.org/10.1200/jco.20.01802 |
| McIndoo, 2016 | McIndoo CC, File AA, Preddy T, Clark CG, Hopko DR. Mindfulness-based therapy and behavioral activation: A randomized controlled trial with depressed college students. Behaviour Research and Therapy. 2016;77:118-28. |
| McKee, 2006 | McKee MD, Zayas LH, Fletcher J, Boyd RC, Nam SH. Results of an intervention to reduce perinatal depression among low-income minority women in community primary care. Journal of Social Service Research. 2006;32(4):63-81. |
| Mennen, 2021 | Mennen, F. E., Palmer Molina, A., Monro, W. L., Duan, L., Stuart, S., & Sosna, T. (2021). Effectiveness of an Interpersonal Psychotherapy (IPT) Group Depression Treatment for Head Start Mothers: A Cluster-Randomized Controlled Trial. J Affect Disord, 280(Pt B), 39-48. https://doi.org/10.1016/j.jad.2020.11.074 |
| Michalak, 2015 | Michalak J, Schultze M, Heidenreich T, Schramm E. A randomized controlled trial on the efficacy of mindfulness-based cognitive therapy and a group version of cognitive behavioral analysis system of psychotherapy for chronically depressed patients. Journal of Consulting and Clinical Psychology. 2015;83(5):951-63. |
| Milgrom, 2005 | Milgrom J, Negri LM, Gemmill AW, McNeil M, Martin PR. A randomized controlled trial of psychological interventions for postnatal depression. British Journal of Clinical Psychology. 2005;44(4):529-42. |
| Milgrom, 2011 | Milgrom J, Holt CJ, Gemmill AW, Ericksen J, Leigh B, Buist A, et al. Treating postnatal depressive symptoms in primary care: A randomised controlled trial of GP management, with and without adjunctive counselling. BMC psychiatry. 2011;11:95. |
| Milgrom, 2015b | Milgrom J, Holt C, Holt CJ, Ross J, Ericksen J, Gemmill AW. Feasibility study and pilot randomised trial of an antenatal depression treatment with infant follow-up. Archives of Women's Mental Health. 2015;18(5):717-30. |
| Milgrom, 2016 | Milgrom J, Danaher BG, Gemmill AW, Holt C, Holt CJ, Seeley JR, et al. Internet cognitive behavioral therapy for women with postnatal depression: A randomized controlled trial of MumMoodBooster. Journal of Medical Internet Research. 2016;18(3):e54. |
| Milgrom, 2021 | Milgrom, J., Danaher, B. G., Seeley, J. R., Holt, C. J., Holt, C., Ericksen, J., Tyler, M. S., Gau, J. M., & Gemmill, A. W. (2021). Internet and Face-to-face Cognitive Behavioral Therapy for Postnatal Depression Compared With Treatment as Usual: Randomized Controlled Trial of MumMoodBooster. J Med Internet Res, 23(12), e17185. https://doi.org/10.2196/17185 |
| Miller, 2002 | Miller L, Weissman M. Interpersonal psychotherapy delivered over the telephone to recurrent depressives. A pilot study. Depression and anxiety. 2002;16(3):114-7. |
| Miranda, 2003 | Miranda J, Chung JY, Green BL, Krupnick J, Siddique J, Revicki DA, et al. Treating depression in predominantly low-income young minority women: A randomized controlled trial. Jama. 2003;290(1):57-65. |
| Mohr, 2000 | Mohr DC, Likosky W, Bertagnolli A, Goodkin DE, Van Der Wende J, Dwyer P, et al. Telephone-administered cognitive-behavioral therapy for the treatment of depressive symptoms in multiple sclerosis. Journal of Consulting and Clinical Psychology. 2000;68(2):356-61. |
| Mohr, 2013 | Mohr DC, Duffecy J, Ho J, Kwasny M, Cai X, Burns MN, et al. A randomized controlled trial evaluating a manualized TeleCoaching protocol for improving adherence to a web-based intervention for the treatment of depression. PLoS One. 2013;8(8):e70086. |
| Moldovan, 2013 | Moldovan R, Cobeanu O, David D. Cognitive bibliotherapy for mild depressive symptomatology: Randomized clinical trial of efficacy and mechanisms of change. Clinical Psychology and Psychotherapy. 2013;20(6):482-93. |
| Montero-Marin, 2016 | Montero-Marín, J., Araya, R., Pérez-Yus, M. C., Mayoral, F., Gili, M., Botella, C., . . . López-Del-Hoyo, Y. (2016). An internet-based intervention for depression in primary Care in Spain: a randomized controlled trial. Journal of medical Internet research, 18(8), e231. |
| Moon, 2021 | Moon, J. R., Huh, J., Song, J., Kang, I. S., Park, S. W., Chang, S. A., . . . Han, J. S. (2021). The effects of rational emotive behavior therapy for depressive symptoms in adults with congenital heart disease. Heart Lung, 50(6), 906-913. doi:10.1016/j.hrtlng.2021.07.011 |
| Mossey, 1996 | Mossey JM, Knott KA, Higgins M, Talerico K. Effectiveness of a psychosocial intervention, interpersonal counseling, for subdysthymic depression in medically ill elderly. The journals of gerontology Series A, Biological sciences and medical sciences. 1996;51(4):M172-8. |
| Mukhtar, 2011 | Mukhtar, F., Oei, T. P., & Yaacob, M. (2011). Effectiveness of group cognitive behaviour therapy augmentation in reducing negative cognitions in the treatment of depression in Malaysia. ASEAN Journal of Psychiatry, 12(1), 50-65. |
| Musa, 2021 | Musa, Z. A., Soh, K. L., Mukhtar, F., Soh, K. Y., Oladele, T. O., & Soh, K. G. (2021). Effectiveness of mindfulness-based cognitive therapy among depressed individuals with disabilities in Nigeria: A randomized controlled trial. Psychiatry Res, 296, 113680. doi:10.1016/j.psychres.2020.113680 |
| Nadort, 2022 | Nadort, E., Schouten, R. W., Boeschoten, R. E., Smets, Y., Chandie Shaw, P., Vleming, L. J., . . . Siegert, C. E. H. (2022). Internet-based treatment for depressive symptoms in hemodialysis patients: A cluster randomized controlled trial. Gen Hosp Psychiatry, 75, 46-53. doi:10.1016/j.genhosppsych.2022.01.008 |
| Naeem, 2013 | Naeem F, Sarhandi I, Gul M, Khalid M, Aslam M, Anbrin A. A multicentre randomised controlled trial of a carer supervised culturally adapted cbt (cacbt) based self-help for depression in pakistan. Journal of Affective Disorders. 2013;156:224-7. |
| Naeem, 2015 | Naeem F, Gul M, Irfan M, Munshi T, Asif A, Rashid S, et al. Brief Culturally adapted CBT (CaCBT) for depression: A randomized controlled trial from Pakistan. Journal of Affective Disorders. 2015;177:101-7. |
| Nakagawa, 2017 | Nakagawa, A., Mitsuda, D., Sado, M., Abe, T., Fujisawa, D., Kikuchi, T., . . . Ono, Y. (2017). Effectiveness of supplementary cognitive-behavioral therapy for pharmacotherapy-resistant depression: A randomized controlled trial. Journal of clinical psychiatry, 78(8), 1126-1135 |
| Nasrin, 2017 | Nasrin, F., Rimes, K., Reinecke, A., Rinck, M., & Barnhofer, T. (2017). Effects of Brief Behavioural Activation on Approach and Avoidance Tendencies in Acute Depression: preliminary Findings. Behavioural and Cognitive Psychotherapy, 45(1), 58-72. |
| Neugebauer, 2006 | Neugebauer R, Kline J, Markowitz JC, Bleiberg KL, Baxi L, Rosing MA, et al. Pilot randomized controlled trial of interpersonal counseling for subsyndromal depression following miscarriage. Journal of Clinical Psychiatry. 2006;67(8):1299-304. |
| Newby, 2013 | Newby, J. M., et al. (2013). "Internet cognitive behavioural therapy for mixed anxiety and depression: a randomized controlled trial and evidence of effectiveness in primary care." Psychol Med 43(12): 2635-2648. |
| Newby, 2014 | Newby JM, Lang T, Werner-Seidler A, Holmes E, Moulds ML. Alleviating distressing intrusive memories in depression: A comparison between computerised cognitive bias modification and cognitive behavioural education. Behaviour Research and Therapy. 2014;56:60-7. |
| Nezu, 1986 - pst | Nezu AM. Efficacy of a social problem-solving therapy approach for unipolar depression. Journal of Consulting and Clinical Psychology. 1986;54(2):196-202. |
| Nezu, 1989 - pst | Nezu AM, Perri MG. Social problem-solving therapy for unipolar depression: An initial dismantling investigation. Journal of Consulting and Clinical Psychology. 1989;57(3):408-13. |
| Ng, 2013 | Ng SE, Tien A, Thayala JN, Ho RC, Chan MF. The effect of life story review on depression of older community-dwelling Chinese adults in Singapore: A preliminary result. International Journal of Geriatric Psychiatry. 2013;28(3):328-30. |
| Ngai, 2015 | Ngai FW, Wong P-C, Leung KY, Chau PH, Chung KF. The effect of telephone-based cognitive-behavioral therapy on postnatal depression: A randomized controlled trial. Psychotherapy and Psychosomatics. 2015;84(5):294-303. |
| Niedermoser, 2020 | Niedermoser DW, Kalak N, Kiyhankhadiv A, et al. Workplace-Related Interpersonal Group Psychotherapy to Improve Life at Work in Individuals With Major Depressive Disorders: a Randomized Interventional Pilot Study. Frontiers in psychiatry. 2020;11. |
| Nollett, 2016 | Nollett CL, Bray N, Bunce C, Casten RJ, Edwards RT, Hegel MT, et al. Depression in Visual Impairment Trial (DEPVIT): A randomized clinical trial of depression treatments in people with low vision. Investigative Ophthalmology and Visual Science. 2016;57(10):4247-54. |
| Noone, 2022 | Noone, D., Payne, J., Stott, J., Aguirre, E., Patel-Palfreman, M. M., Stoner, C., . . . Spector, A. (2022). The Feasibility of a Mindfulness Intervention for Depression in People with Mild Dementia: A Pilot Randomized Controlled Trial. Clinical gerontologist, 1-13. doi:10.1080/07317115.2022.2094741 |
| Northwood, 2020 | Northwood AK, Vukovich MM, Beckman A, et al. Intensive psychotherapy and case management for Karen refugees with major depression in primary care: a pragmatic randomized control trial. BMC Fam Pract. 2020;21(1):17. |
| Nyström, 2017 | Nyström, M., Stenling, A., Sjöström, E., Neely, G., Lindner, P., HassmÈn, P., . . . Carlbring, P. (2017). Behavioral activation versus physical activity via the internet: a randomized controlled trial. Journal of Affective Disorders, 215, 85-93. |
| O'Mahen, 2013a | O'Mahen H, Himle JA, Fedock G, Henshaw E, Flynn H. A pilot randomized controlled trial of cognitive behavioral therapy for perinatal depression adapted for women with low incomes. Depression and anxiety. 2013;30(7):679-87. |
| O'Moore, 2018 | O'Moore K, A., Newby, J. M., Andrews, G., Hunter, D. J., Bennell, K., Smith, J., & Williams, A. D. (2018). Internet Cognitive-Behavioral Therapy for Depression in Older Adults With Knee Osteoarthritis: A Randomized Controlled Trial. Arthritis Care Res (Hoboken), 70(1), 61-70. doi:10.1002/acr.23257 |
| O'Neil, 2014 | O'Neil A, Taylor B, Sanderson K, Cyril S, Chan B, Hawkes AL, et al. Efficacy and feasibility of a tele-health intervention for acute coronary syndrome patients with depression: Results of the "MoodCare" randomized controlled trial. Annals of Behavioral Medicine. 2014;48(2):163-74. |
| Olukolade, 2017 | Olukolade, O., & Osinowo, H. (2017). Efficacy of Cognitive Rehabilitation Therapy on Poststroke Depression among Survivors of First Stroke Attack in Ibadan, Nigeria. Behavioural Neurology, 2017 |
| Omidi, 2013 | Omidi A, Mohammadkhani P, Mohammadi A, Zargar F. Comparing mindfulness based cognitive therapy and traditional cognitive behavior therapy with treatments as usual on reduction of major depressive disorder symptoms. Iranian Red Crescent Medical Journal. 2013;15(2):142-6. |
| Onuigbo, 2019 | Onuigbo, L. N., Eseadi, C., Ebifa, S., Ugwu, U. C., Onyishi, C. N., & Oyeoku, E. K. (2019). Effect of rational emotive behavior therapy program on depressive symptoms among university students with blindness in Nigeria. Journal of Rational-Emotive & Cognitive-Behavior Therapy, 37(1), 17-38. doi:10.1007/s10942-018-0297-3 |
| Pace, 1993 | Pace TM, Dixon DN. Changes in depressive self-schemata and depressive symptoms following cognitive therapy. Journal of Counseling Psychology. 1993;40(3):288. |
| Patel, 2017 | Patel, V., Weobong, B., Weiss, H., Anand, A., Bhat, B., Katti, B., . . . Fairburn, C. (2017). The Healthy Activity Program (HAP), a lay counsellor-delivered brief psychological treatment for severe depression, in primary care in India: a randomised controlled trial. Lancet (london, england), 389(10065), 176-185. |
| Pecheur, 1984 | Pecheur DR, Edwards KJ. A comparison of secular and religious versions of cognitive therapy with depressed Christian college students. Journal of Psychology and Theology. 1984. |
| Peden, 2000 | Peden AR, Hall LA, Rayens MK, Beebe LL. Reducing negative thinking and depressive symptoms in college women. Journal of Nursing Scholarship. 2000;32(2):145-51. |
| Penckofer, 2012 | Penckofer SM, Ferrans C, Mumby P, Byrn M, Emanuele MA, Harrison PR, et al. A psychoeducational intervention (SWEEP) for depressed women with diabetes. Annals of Behavioral Medicine. 2012;44(2):192-206. |
| Petersen, 2014 | Petersen I, Hanass Hancock J, Bhana A, Govender K. A group-based counselling intervention for depression comorbid with HIV/AIDS using a task shifting approach in South Africa: A randomized controlled pilot study. Journal of Affective Disorders. 2014;158:78-84. |
| Pibernik-Okanovic, 2009 | Pibernik-Okanovic M, Begic D, Ajdukovic D, Andrijasevic N, Metelko Z. Psychoeducation versus treatment as usual in diabetic patients with subthreshold depression: Preliminary results of a randomized controlled trial. Trials. 2009;10:78. |
| Piers, 2023 | Piers, R. J., Farchione, T. J., Wong, B., Rosellini, A. J., & Cronin-Golomb, A. (2023). Telehealth Transdiagnostic Cognitive Behavioral Therapy for Depression in Parkinson's Disease: A Pilot Randomized Controlled Trial. Movement Disorders Clinical Practice, 10(1), 79-85. doi:10.1002/mdc3.13587 |
| Pihlaja, 2020 | Pihlaja S, Lahti J, Lipsanen JO, et al. Scheduled Telephone Support for Internet Cognitive Behavioral Therapy for Depression in Patients at Risk for Dropout: Pragmatic Randomized Controlled Trial. J Med Internet Res. 2020;22(7):e15732. |
| Pinniger, 2012 | Pinniger, R., Brown, R. F., Thorsteinsson, E. B., & McKinley, P. (2012). Argentine tango dance compared to mindfulness meditation and a waiting-list control: A randomised trial for treating depression. Complement Ther Med, 20(6), 377-384. doi:10.1016/j.ctim.2012.07.003 |
| Pots, 2014 | Pots WTM, Meulenbeek PAM, Veehof MM, Klungers J, Bohlmeijer ET. The efficacy of mindfulness-based cognitive therapy as a public mental health intervention for adults with mild to moderate depressive symptomatology: A randomized controlled trial. PLoS One. 2014;9(10). |
| Pots, 2016 | Pots WT, Fledderus M, Meulenbeek PA, ten Klooster PM, Schreurs KM, Bohlmeijer ET. Acceptance and commitment therapy as a web-based intervention for depressive symptoms: Randomised controlled trial. The British Journal of Psychiatry. 2016;208(1):69-77. |
| Pott, 2022 | Pott, S. L., Kellett, S., Green, S., Daughters, S., & Delgadillo, J. (2022). Behavioral activation for depression delivered by drug and alcohol treatment workers: A pilot randomized controlled trial. J Subst Abuse Treat, 139, 108769. doi:10.1016/j.jsat.2022.108769 |
| Power, 2012 | Power MJ, Freeman C. A randomized controlled trial of IPT versus CBT in primary care: With some cautionary notes about handling missing values in clinical trials. Clinical Psychology and Psychotherapy. 2012;19(2):159-69. |
| Preschl, 2012 | Preschl B, Maercker A, Wagner B, Forstmeier S, Banos RM, Alcaniz M, et al. Life-review therapy with computer supplements for depression in the elderly: A randomized controlled trial. Aging and Mental Health. 2012;16(8):964-74. |
| Propst, 1992 | Propst LR, Ostrom R, Watkins P, Dean T, Mashburn D. Comparative efficacy of religious and nonreligious cognitive-behavioral therapy for the treatment of clinical depression in religious individuals. Journal of Consulting and Clinical Psychology. 1992;60(1):94-103. |
| Psarraki, 2021 | Psarraki, E. E., Bacopoulou, F., Panagoulias, E., Michou, M., Pelekasis, P., Artemiadis, A., . . . Darviri, C. (2021). The effects of Pythagorean Self-Awareness Intervention on patients with major depressive disorder: A pilot randomized controlled trial. J Psychiatr Res, 138, 326-334. doi:10.1016/j.jpsychires.2021.03.067 |
| Puckering, 2010 | Puckering C, McIntosh E, Hickey A, Longford J. Mellow Babies: A group intervention for infants and mothers experiencing postnatal depression. Counselling Psychology Review. 2010;25(1):28-38. |
| Pugh, 2016 | Pugh, N. E., Hadjistavropoulos, H. D., & Dirkse, D. (2016). A Randomised Controlled Trial of Therapist-Assisted, Internet-Delivered Cognitive Behavior Therapy for Women with Maternal Depression. PLoS ONE, 11(3), e0149186. |
| Qiu, 2013 | Qiu J, Chen W, Gao X, Xu Y, Tong H, Yang M, et al. A randomized controlled trial of group cognitive behavioral therapy for Chinese breast cancer patients with major depression. Journal of psychosomatic obstetrics and gynaecology. 2013;34(2):60-7. |
| Raevuori, 2021 | Raevuori, A., Vahlberg, T., Korhonen, T., Hilgert, O., Aittakumpu-Hyden, R., & Forman-Hoffman, V. (2021). A therapist-guided smartphone app for major depression in young adults: A randomized clinical trial. J Affect Disord, 286, 228-238. doi:10.1016/j.jad.2021.02.007 |
| Ransom, 2008 | Ransom D, Heckman TG, Anderson T, Garske J, Holroyd K, Basta T. Telephone-delivered, interpersonal psychotherapy for HIV-infected rural persons with depression: A pilot trial. Psychiatric Services. 2008;59(8):871-7. |
| Raue, 2019 | Raue, P. J., Sirey, J. A., Dawson, A., Berman, J., & Bruce, M. L. (2019). Lay-delivered behavioral activation for depressed senior center clients: Pilot RCT. International journal of geriatric psychiatry, 34(11), 1715-1723. doi:10.1002/gps.5186 |
| Raya-Tena, 2021 | Raya-Tena, A., Fernández-San-Martin, M. I., Martin-Royo, J., Casañas, R., Sauch-Valmaña, G., Cols-Sagarra, C., . . . Jiménez-Herrera, M. F. (2021). Effectiveness of a Psychoeducational Group Intervention Carried Out by Nurses for Patients with Depression and Physical Comorbidity in Primary Care: Randomized Clinical Trial. Int J Environ Res Public Health, 18(6). doi:10.3390/ijerph18062948 |
| Rehm, 1981 | Rehm LP, Kornblith SJ, O'Hara MW, Lamparski DM, Romano JM, Volkin JI. An evaluation of major components in a self-control therapy program for depression. Behavior modification. 1981;5(4):459-89. |
| Richards, 2015 | Richards D, Timulak L, O'Brien E, Hayes C, Vigano N, Sharry J, et al. A randomized controlled trial of an internet-delivered treatment: Its potential as a low-intensity community intervention for adults with symptoms of depression. Behaviour Research and Therapy. 2015;75:20-31. |
| Richards, 2018 | Richards SH, Dickens C, Anderson R, et al. Assessing the effectiveness of Enhanced Psychological Care for patients with depressive symptoms attending cardiac rehabilitation compared with treatment as usual (CADENCE): a pilot cluster randomised controlled trial. Trials 2018; 19(1). |
| Rief, 2018 | Rief W, Bleichhardt G, Dannehl K, Euteneuer F, Wambach K. Comparing the Efficacy of CBASP with Two Versions of CBT for Depression in a Routine Care Center: a Randomized Clinical Trial. Psychotherapy and psychosomatics 2018; |
| Rizvi, 2015 | Rizvi SJ, Zaretsky A, Schaffer A, Levitt A. Is immediate adjunctive CBT more beneficial than delayed CBT in treating depression?: A Pilot Study. Journal of Psychiatric Practice. 2015;21(2):107-13. |
| Rohricht, 2013 | Rohricht F, Papadopoulos N, Priebe S. An exploratory randomized controlled trial of body psychotherapy for patients with chronic depression. Journal of Affective Disorders. 2013;151(1):85-91. |
| Ross, 1985 | Ross M, Scott M. An evaluation of the effectiveness of individual and group cognitive therapy in the treatment of depressed patients in an inner city health centre. The Journal of the Royal College of General Practitioners. 1985;35(274):239-42. |
| Rude, 1986 | Rude SS. Relative benefits of assertion or cognitive self-control treatment for depression as a function of proficiency in each domain. Journal of Consulting and Clinical Psychology. 1986;54(3):390-4. |
| Ruzickova, 2021 | Ruzickova, T., Carson, J., Murphy, S., & Harmer, C. (2021). P.230 Effects of online behavioural activation on depression during covid-19. European neuropsychopharmacology, 44, S36-S37. doi:10.1016/j.euroneuro.2021.01.059 |
| Safren, 2009 | Safren SA, O'Cleirigh C, Tan JY, Raminani SR, Reilly LC, Otto MW, et al. A randomized controlled trial of cognitive behavioral therapy for adherence and depression (CBT-AD) in HIV-infected individuals. Health Psychology. 2009;28(1):1-10. |
| Safren, 2014 | Safren SA, Gonzalez JS, Wexler DJ, Psaros C, Delahanty LM, Blashill AJ, et al. A randomized controlled trial of cognitive behavioral therapy for adherence and depression (CBT-AD) in patients with uncontrolled type 2 diabetes. Diabetes care. 2014;37(3):625-33. |
| Safren, 2016 | Safren SA, Bedoya CA, O'Cleirigh C, Biello KB, Pinkston MM, Stein MD, et al. Cognitive behavioural therapy for adherence and depression in patients with HIV: A three-arm randomised controlled trial. The Lancet HIV. 2016;3(11):e529-e38. |
| Safren, 2021 | Safren, S. A., O'Cleirigh, C., Andersen, L. S., Magidson, J. F., Lee, J. S., Bainter, S. A., . . . Joska, J. A. (2021). Treating depression and improving adherence in HIV care with task-shared cognitive behavioural therapy in Khayelitsha, South Africa: a randomized controlled trial. J Int AIDS Soc, 24(10), e25823. doi:10.1002/jia2.25823 |
| Saisanan, 2020 | Saisanan Na Ayudhaya, W., Pityaratstian, N., & Jiamjarasrangsi, W. (2020). Effectiveness of Behavioral Activation in Treating Thai Older Adults with Subthreshold Depression Residing in the Community. Clin Interv Aging, 15, 2363-2374. doi:10.2147/cia.S274262 |
| Salamanca-Sanabria, 2020 | Salamanca-Sanabria A, Richards D, Timulak L, et al. A culturally adapted cognitive behavioral internet-delivered intervention for depressive symptoms: Randomized controlled trial. JMIR Mental Health. 2020;7(1). |
| Saloheimo, 2016 | Saloheimo HP, Markowitz J, Saloheimo TH, Laitinen JJ, Sundell J, Huttunen MO, et al. Psychotherapy effectiveness for major depression: A randomized trial in a Finnish community. BMC psychiatry. 2016;16:131. |
| Sander, 2020 | Sander LB, Paganini S, Terhorst Y, et al. Effectiveness of a Guided Web-Based Self-help Intervention to Prevent Depression in Patients with Persistent Back Pain: The PROD-BP Randomized Clinical Trial. JAMA Psychiatry. 2020;77(10):1001-1011. |
| Savari, 2021 | Savari, Y., Mohagheghi, H., & Petrocchi, N. (2021). A preliminary investigation on the effectiveness of compassionate mind training for students with major depressive disorder: A randomized controlled trial. Mindfulness, 12(5), 1159-1172. doi:10.1007/s12671-020-01584-3 |
| Scazufca, 2022 | Scazufca, M., Nakamura, C. A., Seward, N., Moreno-Agostino, D., van de Ven, P., Hollingworth, W., . . . Araya, R. (2022). A task-shared, collaborative care psychosocial intervention for improving depressive symptomatology among older adults in a socioeconomically deprived area of Brazil (PROACTIVE): a pragmatic, two-arm, parallel-group, cluster-randomised controlled trial. Lancet Healthy Longev, 3(10), e690-e702. doi:10.1016/s2666-7568(22)00194-5 |
| Schlicker, 2020 | Schlicker S, Baumeister H, Buntrock C, et al. A web- And mobile-based intervention for comorbid, recurrent depression in patients with chronic back pain on sick leave (get.back): Pilot randomized controlled trial on feasibility, user satisfaction, and effectiveness. JMIR Mental Health. 2020;7(4). |
| Schmidt, 1983 | Schmidt MM, Miller WR. Amount of therapist contact and outcome in a multidimensional depression treatment program. Acta Psychiatrica Scandinavica. 1983;67(5):319-32. |
| Schramm, 2020 | Schramm E, Mack S, Thiel N, Jenkner C, Elsaesser M, Fangmeier T. Interpersonal psychotherapy vs treatment as usual for major depression related to work stress: A pilot randomized controlled study. Frontiers in Psychiatry. 2020;11. |
| Schulberg, 1996 | Schulberg HC, Block MR, Madonia MJ, Scott CP, Rodriguez E, Imber SD, et al. Treating major depression in primary care practice. Eight-month clinical outcomes. Archives of General Psychiatry. 1996;53(10):913-9. |
| Schuster, 2017 | Schuster, R., Leitner, I., Carlbring, P., & Laireiter, A.-R. (2017). Exploring blended group interventions for depression: randomised controlled feasibility study of a blended computer- and multimedia-supported psychoeducational group intervention for adults with depressive symptoms. Internet Interventions, 8, 63-71. |
| Scogin, 1989 | Scogin F, Jamison C, Gochneaur K. Comparative efficacy of cognitive and behavioral bibliotherapy for mildly and moderately depressed older adults. Journal of Consulting and Clinical Psychology. 1989;57(3):403-7. |
| Scott, 1990 | Scott MJ, Stradling SG. Group cognitive therapy for depression produces clinically significant reliable change in community-based settings. Behavioural Psychotherapy. 1990;18(01):1-19. |
| Scott, 1992 | Scott AI, Freeman CP. Edinburgh primary care depression study: Treatment outcome, patient satisfaction, and cost after 16 weeks. BMJ. 1992;304(6831):883-7. |
| Segre, 2015 | Segre LS, Brock RL, O'Hara MW. Depression treatment for impoverished mothers by point-of-care providers: A randomized controlled trial. Journal of Consulting and Clinical Psychology. 2015;83(2):314-24. |
| Selmi, 1990 | Selmi PM, Klein MH, Greist JH, Sorrell SP, Erdman HP. Computer-administered cognitive-behavioral therapy for depression. American Journal of Psychiatry. 1990;147(1):51-6. |
| Serfaty, 2009 | Serfaty MA, Haworth D, Blanchard M, Buszewicz M, Murad S, King M. Clinical effectiveness of individual cognitive behavioral therapy for depressed older people in primary care: A randomized controlled trial. Archives of General Psychiatry. 2009;66(12):1332-40. |
| Serrano, 2004 | Serrano JP, Latorre JM, Gatz M, Montanes J. Life review therapy using autobiographical retrieval practice for older adults with depressive symptomatology. Psychology and Aging. 2004;19(2):270-7. |
| Shaw, 1977 | Shaw BF. Comparison of cognitive therapy and behavior therapy in the treatment of depression. Journal of Consulting and Clinical Psychology. 1977;45(4):543. |
| Sheeber, 2012 | Sheeber LB, Seeley JR, Feil EG, Davis B, Sorensen E, Kosty DB, et al. Development and pilot evaluation of an Internet-facilitated cognitive-behavioral intervention for maternal depression. Journal of Consulting and Clinical Psychology. 2012;80(5):739-49. |
| Sikander, 2019 | Sikander, S., Ahmad, I., Atif, N., Zaidi, A., Vanobberghen, F., Weiss, H. A., . . . Rahman, A. (2019). Delivering the Thinking Healthy Programme for perinatal depression through volunteer peers: a cluster randomised controlled trial in Pakistan. The Lancet Psychiatry, 6(2), 128-139. doi:10.1016/S2215-0366(18)30467-X |
| Simoni, 2013 | Simoni JM, Wiebe JS, Sauceda JA, Huh D, Sanchez G, Longoria V, et al. A preliminary RCT of CBT-AD for adherence and depression among HIV-positive Latinos on the U.S.-Mexico border: The Nuevo Dia study. AIDS and behavior. 2013;17(8):2816-29. |
| Simpson, 2003 | Simpson S, Corney R, Beecham J. A randomized controlled trial to evaluate the effectiveness and cost-effectiveness of psychodynamic counselling for general practice patients with chronic depression. Psychological Medicine. 2003;33(2):229-39. |
| Simson, 2008 | Simson U, Nawarotzky U, Friese G, Porck W, Schottenfeld-Naor Y, Hahn S, et al. Psychotherapy intervention to reduce depressive symptoms in patients with diabetic foot syndrome. Diabetic Medicine. 2008;25(2):206-12. |
| Sinniah, 2017 | Sinniah, A., Oei, T., Maniam, T., & Subramaniam, P. (2017). Positive effects of Individual Cognitive Behavior Therapy for patients with unipolar mood disorders with suicidal ideation in Malaysia: a randomised controlled trial. Psychiatry research, 254, 179-189. |
| Sirey, 2021 | Sirey, J. A., Solomonov, N., Guillod, A., Zanotti, P., Lee, J., Soliman, M., & Alexopoulos, G. S. (2021). PROTECT: a novel psychotherapy for late-life depression in elder abuse victims. Int Psychogeriatr, 33(5), 521-525. doi:10.1017/s1041610221000430 |
| Smit, 2006 | Smit A, Kluiter H, Conradi HJ, Meer K, Tiemens BG, Jenner JA, et al. Short-term effects of enhanced treatment for depression in primary care: Results from a randomized controlled trial. Psychological Medicine. 2006;36(1):15-26. |
| Songprakun, 2012 | Songprakun W, McCann TV. Evaluation of a cognitive behavioural self-help manual for reducing depression: A randomized controlled trial. Journal of psychiatric and mental health nursing. 2012;19(7):647-53. |
| Spek, 2007 | Spek V, Nyklicek I, Smits N, Cuijpers P, Riper H, Keyzer J, et al. Internet-based cognitive behavioural therapy for subthreshold depression in people over 50 years old: A randomized controlled clinical trial. Psychological Medicine. 2007;37(12):1797-806. |
| Spruill, 2021 | Spruill, T. M., Friedman, D., Diaz, L., Butler, M. J., Goldfeld, K. S., O'Kula, S., . . . Devinsky, O. (2021). Telephone-based depression self-management in Hispanic adults with epilepsy: a pilot randomized controlled trial. Transl Behav Med, 11(7), 1451-1460. doi:10.1093/tbm/ibab045 |
| Sreevani, 2013 | Sreevani R, Reddemma K, Chan CL, Leung PP, Wong V, Chan CH. Effectiveness of integrated body-mind-spirit group intervention on the well-being of Indian patients with depression: A pilot study. Journal of Nursing Research. 2013;21(3):179-86. |
| Steel, 2020 | Steel, C., Korrelboom, K., Baksh, M. F., Kingdon, D., Simon, J., Wykes, T., ... & van der Gaag, M. (2020). Positive memory training for the treatment of depression in schizophrenia: A randomised controlled trial. Behaviour Research and Therapy, 135, 103734. |
| Stiles-Shields, 2018 | Stiles-Shields, C., Montague, E., Kwasny, M. J., & Mohr, D. C. (2019). Behavioral and cognitive intervention strategies delivered via coached apps for depression: Pilot trial. Psychological services, 16(2), 233-238. doi:10.1037/ser0000261 |
| Strauss, 2012 | Strauss C, Hayward M, Chadwick P. Group person-based cognitive therapy for chronic depression: A pilot randomized controlled trial. British Journal of Clinical Psychology. 2012;51(3):345-50. |
| Strong, 2008 | Strong V, Waters R, Hibberd C, Murray G, Wall L, Walker J, et al. Management of depression for people with cancer (SMaRT oncology 1): A randomised trial. Lancet. 2008;372(9632):40-8. |
| Stuart, 2022 | Stuart, R., Fischer, H., Leitzke, A. S., Becker, D., Saheba, N., & Coleman, K. J. (2022). The Effectiveness of Internet-Based Cognitive Behavioral Therapy for the Treatment of Depression in a Large Real-World Primary Care Practice: A Randomized Trial. The Permanente journal, 1-8. doi:10.7812/TPP/21.183 |
| Sugg, 2018 | Sugg HVR, Richards DA, Frost J. Morita Therapy for depression (Morita Trial): a pilot randomised controlled trial. BMJ open 2018; 8(8). |
| Swartz, 2008 | Swartz HA, Frank E, Zuckoff A, Cyranowski JM, Houck PR, Cheng Y, et al. Brief interpersonal psychotherapy for depressed mothers whose children are receiving psychiatric treatment. American Journal of Psychiatry. 2008;165(9):1155-62. |
| Szumska, 2020 | Szumska I, Gola M, Rusanowska M, et al. Mindfulness-based cognitive therapy reduces clinical symptoms, but do not change frontal alpha asymmetry in people with major depression disorder. International Journal of Neuroscience. 2020. |
| Takagaki, 2016 | Takagaki K, Okamoto Y, Jinnin R, Mori A, Nishiyama Y, Yamamura T, et al. Behavioral activation for late adolescents with subthreshold depression: A randomized controlled trial. European Child and Adolescent Psychiatry. 2016;25(11):1171-82. |
| Talbot, 2011 | Talbot NL, Chaudron LH, Ward EA, Duberstein PR, Conwell Y, O'Hara MW, et al. A randomized effectiveness trial of interpersonal psychotherapy for depressed women with sexual abuse histories. Psychiatric Services. 2011;62(4):374-80. |
| Taylor, 1977 | Taylor FG, Marshall WL. Experimental analysis of a cognitive-behavioral therapy for depression. Cognitive Therapy and Research. 1977;1(1):59-72. |
| Teasdale, 1984 | Teasdale JD, Fennell MJ, Hibbert GA, Amies PL. Cognitive therapy for major depressive disorder in primary care. The British Journal of Psychiatry. 1984;144:400-6. |
| Teichman, 1995 | Teichman Y, Bar-el Z, Shor H, Sirota P, Elizur A. A comparison of two modalities of cognitive therapy (individual and marital) in treating depression. Psychiatry. 1995;58(2):136-48. |
| Teri, 1997 - pst | Teri L, Logsdon RG, Uomoto J, McCurry SM. Behavioral treatment of depression in dementia patients: A controlled clinical trial. The journals of gerontology Series B, Psychological sciences and social sciences. 1997;52(4):P159-66. |
| Thomas, 2019 | Thomas, S. A., Drummond, A. E., Lincoln, N. B., Palmer, R. L., das Nair, R., Latimer, N. R., . . . Topcu, G. (2019). Behavioural activation therapy for post-stroke depression: the BEADS feasibility RCT. Health Technol Assess, 23(47), 1-176. doi:10.3310/hta23470 |
| Titov, 2010 | Titov N, Andrews G, Davies M, McIntyre K, Robinson E, Solley K. Internet treatment for depression: A randomized controlled trial comparing clinician vs. technician assistance. PLoS One. 2010;5(6):e10939. |
| Titov, 2015a | Titov N, Dear BF, Ali S, Zou JB, Lorian CN, Johnston L, et al. Clinical and cost-effectiveness of therapist-guided internet-delivered cognitive behavior therapy for older adults with symptoms of depression: A randomized controlled trial. Behavior Therapy. 2015;46(2):193-205. |
| Tomasino, 2017 | Tomasino, K., Lattie, E., Ho, J., Palac, H., Kaiser, S., & Mohr, D. (2017). Harnessing Peer Support in an Online Intervention for Older Adults with Depression. American Journal of Geriatric Psychiatry, 25(10), 1109-1119. |
| Tong, 2020 | Tong, P., Bu, P., Yang, Y., Dong, L., Sun, T., & Shi, Y. (2019). Group cognitive behavioural therapy can reduce stigma and improve treatment compliance in major depressive disorder patients. Early intervention in psychiatry. doi:10.1111/eip.12841 |
| Tovote, 2014 | Tovote KA, Fleer J, Snippe E, Peeters A, Emmelkamp PMG, Sanderman R, et al. Individual mindfulness-based cognitive therapy and cognitive behavior therapy for treating depressive symptoms in patients with diabetes: Results of a randomized controlled trial. Diabetes care. 2014;37(9):2427-34. |
| Town, 2017 | Town, J., Abbass, A., Stride, C., & Bernier, D. (2017). A randomised controlled trial of Intensive Short-Term Dynamic Psychotherapy for treatment resistant depression: the Halifax Depression Study. Journal of Affective Disorders, 214, 15-25. |
| Trevillion, 2020 | Trevillion K, Ryan EG, Pickles A, et al. An exploratory parallel-group randomised controlled trial of antenatal Guided Self-Help (plus usual care) versus usual care alone for pregnant women with depression: DAWN trial. Journal of Affective Disorders. 2020;261:187-197. |
| Tulbure, 2018 | Tulbure BT, Andersson G, Salagean N, Pearce M, Koenig HG. Religious versus Conventional Internet-based Cognitive Behavioral Therapy for Depression. J Relig Health 2018; 57(5): 1634-48. |
| Unlu-Ince, 2013 | Unlu Ince B, Cuijpers P, t Hof E, Ballegooijen W, Christensen H, Riper H. Internet-based, culturally sensitive, problem-solving therapy for Turkish migrants with depression: Randomized controlled trial. Journal of Medical Internet Research. 2013;15(10):e227. |
| van Bastelaar, 2011 | van Bastelaar KM, Pouwer F, Cuijpers P, Riper H, Snoek FJ. Web-based depression treatment for type 1 and type 2 diabetic patients: A randomized, controlled trial. Diabetes care. 2011;34(2):320-5. |
| van der Zanden, 2012 | van der Zanden, R., Kramer, J., Gerrits, R., & Cuijpers, P. (2012). Effectiveness of an online group course for depression in adolescents and young adults: a randomized trial. Journal of medical Internet research, 14(3), e86. |
| Van Horne, 2022 | Van Horne, B. S., Nong, Y. H., Cain, C. M., Sampson, M., Greeley, C. S., & Puryear, L. (2022). A promising new model of care for postpartum depression: A randomised controlled trial of a brief home visitation program conducted in Houston, Texas, USA. Health Soc Care Community, 30(5), e2203-e2213. doi:10.1111/hsc.13658 |
| van Lieshout, 2021 | Van Lieshout, R. J., Layton, H., Savoy, C. D., Brown, J. S. L., Ferro, M. A., Streiner, D. L., . . . Hanna, S. (2021). Effect of Online 1-Day Cognitive Behavioral Therapy-Based Workshops Plus Usual Care vs Usual Care Alone for Postpartum Depression: A Randomized Clinical Trial. JAMA Psychiatry, 78(11), 1200-1207. doi:10.1001/jamapsychiatry.2021.2488 |
| Van Lieshout, 2022 | Van Lieshout, R. J., Layton, H., Savoy, C. D., Haber, E., Feller, A., Biscaro, A., . . . Ferro, M. A. (2022). Public Health Nurse-delivered Group Cognitive Behavioural Therapy for Postpartum Depression: A Randomized Controlled Trial. Can J Psychiatry, 67(6), 432-440. doi:10.1177/07067437221074426 |
| Van Luenen, 2018 | van Luenen, S., Garnefski, N., Spinhoven, P., & Kraaij, V. (2018). Guided internet-based intervention for people with HIV and depressive symptoms: a randomised controlled trial in the Netherlands. The lancet HIV, 5(9), e488-e497. |
| Vazquez-Gonzalez, 2013 | Vázquez González FL, Otero Otero P, Torres Iglesias A, Hermida Garcìa E, Blanco Seoane V, Dìaz Fernández O. A brief problem-solving indicated-prevention intervention for prevention of depression in nonprofessional caregivers. Psicothema. 2013;25(1):87-92. |
| Vazquez, 2017 | Vázquez, F. L., Torres, Á., Otero, P., Blanco, V., Díaz, O., & Estévez, L. E. (2017). Analysis of the components of a cognitive-behavioral intervention administered via conference call for preventing depression among non-professional caregivers: A pilot study. Aging Ment Health, 21(9), 938-946. |
| Vazquez, 2020 | Vázquez FL, López L, Torres Á J, et al. Analysis of the Components of a Cognitive-Behavioral Intervention for the prevention of Depression Administered via Conference Call to Nonprofessional Caregivers: A Randomized Controlled Trial. Int J Environ Res Public Health. 2020;17(6). |
| Verduyn, 2003 | Verduyn C, Barrowclough C, Roberts J, Tarrier N, Harrington R. Maternal depression and child behaviour problems: Randomised placebo-controlled trial of a cognitive-behavioural group intervention. British Journal of Psychiatry. 2003;183(OCT.):342-8. |
| Vernmark, 2010 | Vernmark K, Lenndin J, Bjärehed J, Carlsson M, Karlsson J, Oberg J, et al. Internet administered guided self-help versus individualized e-mail therapy: A randomized trial of two versions of CBT for major depression. Behaviour Research and Therapy. 2010;48(5):368-76. |
| Vigod, 2021 | Vigod, S. N., Slyfield Cook, G., Macdonald, K., Hussain-Shamsy, N., Brown, H. K., de Oliveira, C., . . . Dennis, C.-L. (2021). Mother matters: Pilot randomized wait‐list controlled trial of an online therapist‐facilitated discussion board and support group for postpartum depression symptoms. Depress Anxiety, 38(8), 816-825. doi:10.1002/da.23163 |
| Vitriol, 2009 | Vitriol VG, Ballesteros ST, Florenzano RU, Weil KP, Benadof DF. Evaluation of an outpatient intervention for women with severe depression and a history of childhood trauma. Psychiatric Services. 2009;60(7):936-42. |
| Warmerdam, 2008 | Warmerdam L, Straten A, Twisk J, Riper H, Cuijpers P. Internet-based treatment for adults with depressive symptoms: Randomized controlled trial. Journal of Medical Internet Research. 2008;10(4):e44. |
| Westerhof, 2019 | Westerhof, G. J., Lamers, S. M. A., Postel, M. G., & Bohlmeijer, E. T. (2019). Online Therapy for Depressive Symptoms: An Evaluation of Counselor-Led and Peer-Supported Life Review Therapy. The Gerontologist, 59(1), 135-146. doi:10.1093/geront/gnx140 |
| Wickberg, 1996 | Wickberg B, Hwang CP. Counselling of postnatal depression: A controlled study on a population based Swedish sample. Journal of Affective Disorders. 1996;39(3):209-16. |
| Wiersma, 2014 | Wiersma JE, Schaik DJF, Hoogendorn AW, Dekker JJ, Van HL, Schoevers RA, et al. The effectiveness of the cognitive behavioral analysis system of psychotherapy for chronic depression: A randomized controlled trial. Psychotherapy and Psychosomatics. 2014;83(5):263-9. |
| Wiklund, 2010 | Wiklund I, Mohlkert P, Edman G. Evaluation of a brief cognitive intervention in patients with signs of postnatal depression: A randomized controlled trial. Acta obstetricia et gynecologica Scandinavica. 2010;89(8):1100-4. |
| Williams, 2013b | Williams C, Wilson P, Morrison J, McMahon A, Andrew W, Allan L, et al. Guided self-help cognitive behavioural therapy for depression in primary care: A randomised controlled trial. PLoS One. 2013;8(1):e52735. |
| Wilson, 1983 | Wilson PH, Goldin JC, Charbonneau-Powis M. Comparative efficacy of behavioral and cognitive treatments of depression. Cognitive Therapy and Research. 1983;7(2):111-24. |
| Wollersheim, 1991 | Wollersheim JP, Wilson GL. Group treatment of unipolar depression: A comparison of coping, supportive, bibliotherapy, and delayed treatment groups. Professional Psychology: Research and Practice. 1991;22(6):496. |
| Wong, 2008a | Wong DF. Cognitive and health-related outcomes of group cognitive behavioural treatment for people with depressive symptoms in Hong Kong: Randomized wait-list control study. Australian and New Zealand Journal of Psychiatry. 2008;42(8):702-11. |
| Wong, 2008b | Wong DF. Cognitive behavioral treatment groups for people with chronic depression in Hong Kong: A randomized wait-list control design. Depression and anxiety. 2008;25(2):142-8. |
| Wong, 2018 | Wong SYS, Sun YY, Chan ATY, et al. Treating Subthreshold Depression in Primary Care: a Randomized Controlled Trial of Behavioral Activation With Mindfulness. Annals of family medicine 2018; 16(2): 111‐9. |
| Wright, 2005 | Wright JH, Wright AS, Albano AM, Basco MR, Goldsmith LJ, Raffield T, et al. Computer-assisted cognitive therapy for depression: Maintaining efficacy while reducing therapist time. American Journal of Psychiatry. 2005;162(6):1158-64. |
| Wright, 2022 | Wright, J. H., Owen, J., Eells, T. D., Antle, B., Bishop, L. B., Girdler, R., . . . Ali, S. (2022). Effect of Computer-Assisted Cognitive Behavior Therapy vs Usual Care on Depression Among Adults in Primary Care: A Randomized Clinical Trial. JAMA Netw Open, 5(2), e2146716. doi:10.1001/jamanetworkopen.2021.46716 |
| Xie, 2019 | Xie, J., He, G., Ding, S., Pan, C., Zhang, X., Zhou, J., & Iennaco, J. D. (2019). A randomized study on the effect of modified behavioral activation treatment for depressive symptoms in rural left-behind elderly. Psychotherapy research : journal of the Society for Psychotherapy Research, 29(3), 372-382. doi:10.1080/10503307.2017.1364444 |
| Yang, 2018 | Yang X, Zhao J, Chen Y, Zu S, Zhao J. Comprehensive self-control training benefits depressed college students: a six-month randomized controlled intervention trial. Journal of affective disorders 2018; 226: 251‐60. |
| Yeung, 2017 | Yeung, A., Wang, F., Feng, F., Zhang, J., Cooper, A., Hong, L., . . . Fava, M. (2017). Outcomes of an online computerized cognitive behavioral treatment program for treating chinese patients with depression: A pilot study. Asian journal of psychiatry. doi:10.1016/j.ajp.2017 |
| Ying, 2022 | Ying, Y., Ji, Y., Kong, F., Wang, M., Chen, Q., Wang, L., . . . Ruan, L. (2022). Efficacy of an internet-based cognitive behavioral therapy for subthreshold depression among Chinese adults: a randomized controlled trial. Psychological medicine, 1-11. doi:10.1017/S0033291722000599 |
| Yuan, 2020 | Yuan J, Yin Y, Tang X, et al. Culturally adapted and lay-delivered cognitive behaviour therapy for older adults with depressive symptoms in rural China: a pilot trial. Behavioural and cognitive psychotherapy. 2020:1-5. |
| Zemestani, 2016 | Zemestani M, Davoodi I, Honarmand MM, Zargar Y, Ottaviani C. Comparative effects of group metacognitive therapy versus behavioural activation in moderately depressed students. Journal of Mental Health. 2016;25(6):479-85. |
| Zemestani, 2020b | Zemestani, M., & Fazeli Nikoo, Z. (2020). Effectiveness of mindfulness-based cognitive therapy for comorbid depression and anxiety in pregnancy: a randomized controlled trial. Archives of Women's Mental Health. doi:10.1007/s00737-019-00962-8 |
| Zhao, 2021 | Zhao, Y., Lin, Q., & Wang, J. (2021). An evaluation of a prenatal individualised mixed management intervention addressing breastfeeding outcomes and postpartum depression: A ramdomised controlled trial. J Clin Nurs, 30(9-10), 1347-1359. doi:10.1111/jocn.15684 |
| Zu, 2014 | Zu S, Xiang Y-T, Liu J, Zhang L, Wang G, Ma X, et al. A comparison of cognitive-behavioral therapy, antidepressants, their combination and standard treatment for Chinese patients with moderate–severe major depressive disorders. Journal of Affective Disorders. 2014;152-154:262-7. |

## Appendix B. Selected characteristics of included studies

| study | Ther | Ctr | For-mat | N sess | C | M age | Pr wom | Recr | Dx | Targ  grp | N  arms | N outc | sg | ac | ba | itt | rob | year |
| --- | --- | --- | --- | --- | --- | --- | --- | --- | --- | --- | --- | --- | --- | --- | --- | --- | --- | --- |
| Aagaard, 2017 | cbt | cau | grp | 8 | eu | 48 | 0.71 | clin | mood | adul | 2 | 1 | + | + | sr | - | 3 | 2017 |
| Abas, 2018 | pst | cau | ind | 5 | oth | 37,8 | 0.66 | oth | cut | med | 2 | 2 | + | + | sr | + | 4 | 2018 |
| Abbas, 2023 | cbt | wl | ind | 9 | oth | 37,3 | 0.55 | clin | cut | med | 2 | 1 | - | - | sr | - | 1 | 2023 |
| Afonso, 2009 | lrt | cau | ind | 5 | eu | 76 | 0.79 | clin | cut | old | 2 | 1 | - | - | + | - | 1 | 2009 |
| Ahmadpanah, 2016 - mdm | 3rd | cau | ind | 8 | oth | 36,49 | 1.00 | oth | cut | med | 3 | 1 | + | + | sr | - | 3 | 2016 |
| Ahmadpanah, 2016 - smt | other psy | cau | grp | 8 | oth | 36,49 | 1.00 | oth | cut | med | 3 | 1 | + | + | sr | - | 3 | 2016 |
| Alhusen, 2021 | cbt | cau | grp | 5 | us | 24,5 | 1.00 | oth | cut | ppd | 2 | 1 | - | - | + | - | 1 | 2021 |
| Allart van Dam, 2003 | cbt | cau | grp | 12 | eu | 45,5 | 0.62 | com | cut | adul | 2 | 1 | - | - | sr | - | 1 | 2003 |
| Amani, 2021 | cbt | wl | grp | 9 | can | 31,55 | 1.00 | com | cut | ppd | 2 | 1 | - | - | + | + | 2 | 2021 |
| Ammerman, 2013 | cbt | cau | ind | 11 | us | 21,9 | 1.00 | oth | mood | ppd | 2 | 3 | - | + | + | + | 3 | 2013 |
| Araya, 2021 - lima | bat | cau | gsh | 18 | oth | 57 | 0.85 | clin | cut | med | 2 | 1 | + | + | + | + | 4 | 2021 |
| Araya, 2021 - sao paulo | bat | cau | gsh | 18 | oth | 57 | 0.85 | clin | cut | med | 2 | 1 | + | + | + | + | 4 | 2021 |
| Arean, 1993 - lrt | lrt | wl | grp | 12 | us | 66,49 | 0.75 | com | mood | old | 3 | 3 | - | - | sr | - | 1 | 1993 |
| Arean, 1993 - pst | pst | wl | grp | 12 | us | 66,49 | 0.75 | com | mood | old | 3 | 3 | - | - | sr | - | 1 | 1993 |
| Au, 2022 | cbt | cau | oth | 12 | eas |  |  | oth | cut | med | 2 | 1 | - | - | sr | - | 1 | 2022 |
| Ayen, 2004 - cbt | cbt | wl | grp | 12 | eu | 51,3 | 1.00 | com | mood | oth | 3 | 2 | - | - | sr | - | 1 | 2004 |
| Ayen, 2004 - sup | sup | wl | grp | 12 | eu | 51,3 | 1.00 | com | mood | oth | 3 | 2 | - | - | sr | - | 1 | 2004 |
| Barnhofer, 2009 | 3rd | cau | grp | 6 | uk | 41,93 | 0.68 | com | mood | adul | 2 | 1 | + | + | sr | + | 4 | 2009 |
| Basirat, 2022 | cbt | cau | grp | 10 | oth | 31,7 | 1.00 | oth | mood | oth | 2 | 1 | + | + | sr | + | 4 | 2022 |
| Baumeister, 2021 | cbt | cau | gsh | 6 | eu | 49,9 | 0.6 | oth | mood | med | 2 | 3 | + | + | + | + | 4 | 2021 |
| Baumgartner, 2021 - alc | cbt | wl | gsh | 4 | eu | 42,8 | 0.48 | com | cut | adul | 3 | 1 | + | + | + | + | 4 | 2021 |
| Baumgartner, 2021 - alc+dep | cbt | wl | gsh | 4 | eu | 42,8 | 0.48 | com | cut | adul | 3 | 1 | + | + | + | + | 4 | 2021 |
| Beach, 1992 - bmt | cbt | wl | oth | 18 | us | 39,14 | 1.00 | com | mood | oth | 3 | 1 | - | - | sr | - | 1 | 1992 |
| Beach, 1992 - cbt | cbt | wl | ind | 18 | us | 39,14 | 1.00 | com | mood | oth | 3 | 1 | - | - | sr | - | 1 | 1992 |
| Bedard, 2014 | 3rd | wl | grp | 10 | can | 46,46 | 0.45 | com | cut | med | 2 | 3 | + | + | sr | - | 3 | 2014 |
| Beeber, 2010 | ipt | cau | ind | 16 | us | 26 | 1.00 | oth | cut | oth | 2 | 1 | + | - | sr | - | 2 | 2010 |
| Bendig, 2021 | cbt | wl | gsh | 3 | eu | 56,4 | 0.35 | oth | cut | med | 2 | 1 | + | + | + | + | 4 | 2021 |
| Berger, 2011 | cbt | wl | gsh | 9 | eu | 38,8 | 0.7 | com | mood | adul | 2 | 1 | + | + | sr | + | 4 | 2011 |
| Beutel, 2014 | dyn | cau | ind | 18 | eu | 51,8 | 1.00 | oth | mood | med | 2 | 1 | + | + | + | + | 4 | 2014 |
| Bilich, 2008 | cbt | wl | gsh | 8 | au | 41,52 | 0.64 | com | cut | adul | 2 | 2 | + | - | sr | - | 2 | 2008 |
| Boele, 2018 | pst | wl | gsh | 5 | eu | 44,9 | 0.55 | com | cut | med | 2 | 1 | + | + | sr | + | 4 | 2018 |
| Boeschoten, 2017 | pst | wl | gsh | 5 | eu | 48,9 | 0.8 | com | cut | med | 2 | 1 | + | + | sr | + | 4 | 2017 |
| Bohlmeijer, 2011 | 3rd | wl | grp | 7 | eu | 49,02 | 0.82 | com | cut | adul | 2 | 1 | + | + | sr | + | 4 | 2011 |
| Bolton, 2003 | ipt | cau | grp | 16 | oth | 45,23 | 0.51 | oth | mood | adul | 2 | 1 | + | - | + | + | 3 | 2003 |
| Bower, 2021 | other psy | wl | grp | 5 | us | 45,4 | 1.00 | oth | cut | med | 2 | 1 | - | - | + | + | 2 | 2021 |
| Bowman, 1995 - cbt | cbt | wl | gsh | 4 | us | 36,2 | 0.63 | com | cut | adul | 3 | 2 | - | - | sr | - | 1 | 1995 |
| Bowman, 1995 - set | pst | wl | gsh | 4 | us | 36,2 | 0.63 | com | cut | adul | 3 | 2 | - | - | sr | - | 1 | 1995 |
| Brown, 1984 - grp | cbt | wl | grp | 12 | us | 36,5 | 0.7 | com | mood | adul | 4 | 2 | - | - | sr | - | 1 | 1984 |
| Brown, 1984 - ind | cbt | wl | ind | 12 | us | 36,5 | 0.7 | com | mood | adul | 4 | 2 | - | - | sr | - | 1 | 1984 |
| Brown, 1984 - tel | cbt | wl | gsh | 12 | us | 36,5 | 0.7 | com | mood | adul | 4 | 2 | - | - | sr | - | 1 | 1984 |
| Buhrman, 2015 | cbt | wl | gsh | 6 | eu | 50,69 | 0.85 | com | cut | med | 2 | 1 | + | + | sr | + | 4 | 2015 |
| Buntrock, 2015 | other psy | cau | gsh | 5 | eu | 45 | 0.74 | com | cut | adul | 2 | 1 | + | + | sr | + | 4 | 2015 |
| Burns, 2007 | other psy | cau | ind | 6 | uk | 81,1 | 0.77 | oth | cut | med | 2 | 1 | + | + | sr | + | 4 | 2007 |
| Burns, 2013 | cbt | cau | ind | 12 | uk | 29,15 | 1.00 | oth | cut | ppd | 2 | 3 | + | + | sr | + | 4 | 2013 |
| Carlbring, 2013 | 3rd | wl | gsh | 5 | eu | 44,4 | 0.83 | com | mood | adul | 2 | 2 | + | + | sr | + | 4 | 2013 |
| Carr, 2017 | cbt | cau | grp | 20 | eu | 41 | 0.66 | clin | mood | adul | 2 | 3 | - | - | + | + | 2 | 2017 |
| Carta, 2012 | cbt | cau | ind | 12 | eu | 42,48 | 0.66 | clin | mood | adul | 2 | 1 | - | - | sr | - | 1 | 2012 |
| Casanas, 2012 | cbt | cau | grp | 9 | eu | 53,38 | 0.89 | clin | mood | adul | 2 | 1 | + | + | sr | + | 4 | 2012 |
| Castonguay, 2004 | cbt | wl | ind | 16 | us | 38,8 | 0.75 | com | mood | adul | 2 | 2 | - | - | sr | - | 1 | 2004 |
| Chan, 2012 - cbt | cbt | wl | grp | 10 | eas | 46,43 | 0.82 | clin | mood | adul | 3 | 2 | - | + | + | - | 2 | 2012 |
| Chan, 2012 - dmbi | cbt | wl | grp | 10 | eas | 46,43 | 0.82 | clin | mood | adul | 3 | 2 | - | + | sr | - | 2 | 2012 |
| Chan, 2013 | lrt | cau | ind | 5 | eas | 69,7 | 0.81 | oth | cut | old | 2 | 1 | + | - | sr | + | 3 | 2013 |
| Chen, 2000 | sup | cau | grp | 4 | eas | 29,1 | 1.00 | oth | cut | ppd | 2 | 1 | - | - | sr | - | 1 | 2000 |
| Chesney, 2003 | other psy | wl | grp | 10 | us | 39 | 0.00 | com | cut | med | 2 | 1 | - | - | sr | - | 1 | 2003 |
| Chiang, 2015 | cbt | cau | grp | 12 | eas | 46,14 | 0.63 | clin | mood | adul | 2 | 2 | + | + | + | - | 3 | 2015 |
| Cho, 2008 | cbt | cau | ind | 9 | eas | 29,62 | 1.00 | oth | mood | ppd | 2 | 1 | - | - | sr | - | 1 | 2008 |
| Choi, 2012 | cbt | wl | gsh | 6 | au | 39 | 0.8 | com | mood | oth | 2 | 3 | + | + | sr | + | 4 | 2012 |
| Chowdhary, 2016 | bat | cau | ind | 7 | oth | 40,57 | 0.69 | oth | cut | adul | 2 | 1 | + | - | sr | + | 3 | 2016 |
| Choy, 2016 | lrt | wl | grp | 6 | eas | 78,66 | 0.65 | oth | cut | old | 2 | 1 | - | - | sr | - | 1 | 2016 |
| Clark, 2003 - ipt | ipt | wl | grp | 12 | us | 31,42 | 1.00 | com | mood | ppd | 3 | 2 | - | - | sr | - | 1 | 2003 |
| Clark, 2003 -m-itg | other psy | wl | grp | 12 | us | 31,42 | 1.00 | com | mood | ppd | 3 | 2 | - | - | sr | - | 1 | 2003 |
| Clark, 2008 | other psy | wl | grp | 12 | us | 30,86 | 1.00 | com | mood | ppd | 2 | 1 | - | - | sr | - | 1 | 2008 |
| Cohen, 2010 | other psy | wl | oth | 5 | us | 43,2 | 1.00 | com | mood | oth | 2 | 2 | - | - | + | - | 1 | 2010 |
| Cooper, 2003 - cbt | cbt | cau | ind | 10 | uk | 27,69 | 1.00 | oth | mood | ppd | 4 | 1 | + | + | sr | + | 4 | 2003 |
| Cooper, 2003 - dyn | dyn | cau | ind | 10 | uk | 27,69 | 1.00 | oth | mood | ppd | 4 | 1 | + | + | sr | + | 4 | 2003 |
| Cooper, 2003 - sup | sup | cau | ind | 10 | uk | 27,69 | 1.00 | oth | mood | ppd | 4 | 1 | + | + | sr | + | 4 | 2003 |
| Cramer, 2011 | cbt | cau | grp | 12 | uk | 42,47 | 1.00 | com | cut | oth | 2 | 1 | + | - | sr | + | 3 | 2011 |
| De Groot, 2019 | cbt | cau | ind | 10 | us | 56 | 0.77 | com | mood | med | 2 | 1 | + | + | sr | + | 4 | 2019 |
| De Jong, 2018 | 3rd | wl | grp | 7 | us | 50,7 | 0.75 | com | mood | med | 2 | 2 | - | - | - | + | 1 | 2018 |
| Dekker, 2012 | cbt | cau | ind | 1 | us | 66 | 0.74 | oth | cut | med | 2 | 1 | + | + | sr | + | 4 | 2012 |
| Demir, 2022 | cbt | cau | grp | 6 | oth | 20,1 | 0.937 | oth | cut | stud | 2 | 1 | + | + | sr | - | 3 | 2022 |
| Dennis, 2020 | ipt | cau | tel | 12 | can |  | 1.00 | com | mood | ppd | 2 | 1 | + | - | sr | - | 2 | 2020 |
| Desautels, 2018 | cbt | wl | ind | 8 | can | 57,1 | 1.00 | oth | cut | med | 2 | 3 | + | + | + | + | 4 | 2018 |
| Dimidjian, 2017 | bat | cau | oth | 10 | us | 28,75 | 1.00 | oth | cut | ppd | 2 | 1 | + | + | sr | + | 4 | 2017 |
| Dindo, 2012 | 3rd | wl | grp | 1 | us | 32,8 | 0.93 | oth | mood | med | 2 | 2 | - | - | + | + | 2 | 2012 |
| Dobkin, 2011 | cbt | cau | ind | 10 | us | 64,56 | 0.4 | com | mood | med | 2 | 2 | + | - | sr | + | 3 | 2011 |
| Dobkin, 2020 | cbt | cau | tel | 10 | us | 65,62 | 0.54 | oth | mood | med | 2 | 2 | + | + | sr | + | 4 | 2020 |
| Dobkin, 2021 | cbt | cau | tel | 10 | us | 66,84 | 0.00 | com | mood | med | 2 | 2 | + | + | + | + | 4 | 2021 |
| Doering, 2013 | cbt | cau | ind | 8 | us | 63,62 | 0.31 | oth | mood | med | 2 | 1 | + | - | sr | + | 3 | 2013 |
| Dong, 2019 | lrt | cau | tel | 6 | eas | 59,1 | 0.5 | oth | cut | med | 2 | 2 | + | + | + | + | 4 | 2019 |
| Dowrick, 2000 | pst | cau | ind | 6 | eu | 44,9 | 0.65 | oth | mood | adul | 2 | 1 | + | + | sr | + | 4 | 2000 |
| Duarte, 2009 | cbt | cau | grp | 12 | oth | 53,23 | 0.59 | oth | mood | med | 2 | 2 | + | + | sr | + | 4 | 2009 |
| Dwight-Johnson, 2011 | cbt | cau | tel | 5 | us | 39,81 | 0.78 | oth | cut | oth | 2 | 2 | + | - | sr | + | 3 | 2011 |
| Ebert, 2014 | pst | wl | gsh | 5 | eu | 47,1 | 0.83 | com | cut | oth | 2 | 1 | + | + | sr | + | 4 | 2014 |
| Ebert, 2018 | other psy | wl | gsh | 5 | eu | 44,2 | 0.8 | com | cut | adul | 2 | 3 | + | + | + | + | 4 | 2018 |
| Ede, 2020 | cbt | wl | grp | 12 | oth |  | 0.56 | oth | cut | stud | 2 | 1 | + | - | sr | + | 3 | 2020 |
| Ekers, 2011 | bat | wl | ind | 12 | uk | 44,72 | 0.62 | clin | mood | adul | 2 | 1 | + | + | sr | + | 4 | 2011 |
| Ekkers, 2011 | cbt | cau | grp | 7 | eu | 72,7 | 0.77 | clin | mood | old | 2 | 2 | - | + | sr | - | 2 | 2011 |
| Embling, 2002 | cbt | wl | grp | 12 | uk |  |  | clin | mood | adul | 2 | 1 | - | - | sr | + | 2 | 2002 |
| Eseadi, 2018 | cbt | cau | grp | 12 | oth | 35,3 | 0.00 | oth | cut | oth | 2 | 1 | - | - | sr | + | 2 | 2018 |
| Eseadi, 2022 | cbt | wl | ind | 12 | oth | 18,7 | 0.37 | oth | cut | stud | 2 | 1 | + | + | sr | + | 4 | 2022 |
| Euteneuer, 2017 - cbt-c | cbt | wl | ind | 16 | eu | 37,3 | 0.49 | com | mood | adul | 3 | 1 | + | - | sr | + | 3 | 2017 |
| Euteneuer, 2017 - cbt-e | cbt | wl | ind | 16 | eu | 37,3 | 0.49 | com | mood | adul | 3 | 1 | + | - | sr | + | 3 | 2017 |
| Euteneuer, 2022 | cbt | wl | ind | 14 | eu | 30,31 | 0.6 | com | mood | adul | 2 | 2 | + | - | sr | + | 3 | 2022 |
| Ewais, 2021 | 3rd | wl | ind | 8 | au | 22 | 0.63 | clin | mood | med | 2 | 1 | + | + | sr | + | 4 | 2021 |
| Fann, 2015 - ftf | cbt | cau | ind | 9 | us | 45,8 | 0.37 | com | mood | med | 3 | 2 | + | - | + | - | 2 | 2015 |
| Fann, 2015 - tel | cbt | cau | tel | 10 | us | 45,8 | 0.37 | com | mood | med | 3 | 2 | + | - | + | - | 2 | 2015 |
| Faramarzi, 2008 | cbt | cau | grp | 10 | oth | 28,35 | 1.00 | oth | cut | oth | 2 | 1 | - | - | sr | - | 1 | 2008 |
| Fledderus, 2012 - act-e | 3rd | wl | gsh | 9 | eu | 42 | 0.7 | com | cut | adul | 3 | 1 | + | - | sr | + | 3 | 2012 |
| Fledderus, 2012 - act-m | 3rd | wl | gsh | 9 | eu | 42 | 0.7 | com | cut | adul | 3 | 1 | + | - | sr | + | 3 | 2012 |
| Floyd, 2004 - gsh | cbt | wl | gsh | 4 | us | 68 | 0.76 | com | mood | old | 3 | 2 | - | - | + | - | 1 | 2004 |
| Floyd, 2004 - ind | cbt | wl | ind | 16 | us | 68 | 0.76 | com | mood | old | 3 | 2 | - | - | + | - | 1 | 2004 |
| Folke, 2012 | 3rd | cau | oth | 6 | eu | 43,24 | 0.88 | oth | mood | oth | 2 | 1 | - | - | sr | + | 2 | 2012 |
| Fonagy, 2015 | dyn | cau | ind | 60 | uk | 44,33 | 0.66 | clin | mood | adul | 2 | 2 | + | + | + | + | 4 | 2015 |
| Forand, 2018 | cbt | wl | gsh | 6 | us | 33 | 0.75 | com | cut | adul | 2 | 2 | + | - | - | + | 2 | 2018 |
| Forsell, 2017 | cbt | cau | gsh | 5 | eu | 31,01 | 1.00 | com | mood | ppd | 2 | 2 | + | + | sr | + | 4 | 2017 |
| Frangou, 2021 | cbt | cau | ind | 3 | uk | 60 | 1.00 | oth | cut | med | 2 | 1 | - | + | + | + | 3 | 2021 |
| Freedland, 2009 - cbt | cbt | cau | ind | 11 | us | 60,65 | 0.5 | oth | mood | med | 3 | 2 | + | + | sr | + | 4 | 2009 |
| Freedland, 2009 - sup | sup | cau | ind | 8 | us | 60,65 | 0.5 | oth | mood | med | 3 | 2 | + | + | sr | + | 4 | 2009 |
| Freedland, 2015 | cbt | cau | ind | 11 | us | 55,8 | 0.46 | oth | mood | med | 2 | 3 | + | + | + | + | 4 | 2015 |
| Fuhr, 2019 | cbt | cau | ind | 10 | oth | 25,18 | 1.00 | oth | cut | ppd | 2 | 1 | - | + | sr | + | 3 | 2019 |
| Funderburk, 2021 | bat | cau | ind | 4 | us | 53 | 0.09 | clin | cut | oth | 2 | 1 | - | + | + | + | 3 | 2021 |
| Furukawa, 2012 | cbt | wl | tel | 7 | eas | 39,35 | 0.22 | oth | cut | oth | 2 | 2 | + | + | sr | + | 4 | 2012 |
| Garcia-Pena, 2015 | cbt | cau | grp | 12 | oth | 70,8 | 0.83 | clin | cut | old | 2 | 1 | + | - | sr | - | 2 | 2015 |
| Gawrysiak, 2009 | bat | cau | ind | 1 | us | 18,4 | 0.8 | com | cut | stud | 2 | 1 | - | - | sr | + | 2 | 2009 |
| Gellis, 2008 | pst | cau | ind | 6 | us | 77,4 | 0.87 | oth | cut | med | 2 | 2 | + | - | + | - | 2 | 2008 |
| Gellis, 2010 | pst | cau | ind | 6 | us | 75,9 | 0.92 | oth | cut | med | 2 | 2 | - | + | sr | - | 2 | 2010 |
| Geraedts, 2014 | cbt | cau | gsh | 6 | eu | 43,4 | 0.62 | oth | cut | oth | 2 | 1 | + | + | sr | + | 4 | 2014 |
| Ghorbani, 2021 | 3rd | wl | grp | 8 | oth | 45 | 1.00 | oth | cut | med | 2 | 1 | - | - | + | - | 1 | 2021 |
| Gibbons, 2012 | dyn | cau | ind | 7 | us | 41,23 | 0.87 | clin | cut | adul | 2 | 2 | - | - | - | - | 0 | 2012 |
| Gitlin, 2013 | other psy | wl | ind | 8 | us | 69,6 | 0.78 | com | cut | old | 2 | 2 | + | + | sr | + | 4 | 2013 |
| Goodman, 2015 | other psy | cau | ind | 8 | us | 30,7 | 1.00 | oth | cut | ppd | 2 | 1 | - | + | sr | + | 3 | 2015 |
| Greenberg, 2018 | 3rd | wl | grp | 7 | us | 38,5 | 0.63 | com | cut | adul | 2 | 2 | - | - | + | - | 1 | 2018 |
| Grote, 2009 | ipt | cau | ind | 8 | us | 24,51 | 1.00 | oth | cut | ppd | 2 | 2 | - | - | sr | + | 2 | 2009 |
| Gureje, 2022 | other psy | cau | ind | 6 | oth | 18 | 1.00 | oth | mood | ppd | 2 | 1 | + | + | + | + | 4 | 2022 |
| Hagen, 2017 | 3rd | wl | ind | 10 | eu | 33,7 | 0.49 | com | mood | adul | 2 | 1 | + | - | sr | + | 3 | 2017 |
| Hallford, 2016 | lrt | cau | ind | 6 | au | 20,8 | 0.5 | clin | cut | oth | 2 | 1 | - | + | sr | + | 3 | 2016 |
| Hallgren, 2015 | cbt | cau | gsh | 8 | eu | 43 | 0.73 | clin | cut | adul | 2 | 1 | + | + | + | - | 3 | 2015 |
| Hamamci, 2006 - cbt | cbt | cau | grp | 11 | oth | 19,52 | 0.48 | com | cut | stud | 3 | 1 | - | - | sr | - | 1 | 2006 |
| Hamamci, 2006 - cbt+dr | cbt | cau | grp | 11 | oth | 19,52 | 0.48 | com | cut | stud | 3 | 1 | - | - | sr | - | 1 | 2006 |
| Hamdan-Mansour, 2009 | cbt | cau | grp | 10 | oth |  | 0.45 | com | cut | stud | 2 | 1 | + | + | sr | - | 3 | 2009 |
| Han, 2020 - cbt | cbt | wl | grp | 10 | eas | 46,94 | 0.77 | clin | mood | adul | 3 | 2 | + | + | + | - | 3 | 2020 |
| Han, 2020 - mbt | other psy | wl | grp | 10 | eas | 47,06 | 0.88 | clin | mood | adul | 3 | 2 | + | + | + | - | 3 | 2020 |
| Haringsma, 2006 | cbt | wl | grp | 9 | eu | 64,2 | 0.69 | com | cut | old | 2 | 2 | - | - | sr | + | 2 | 2005 |
| Harley, 2008 | 3rd | wl | grp | 14 | us | 41,8 | 0.75 | clin | mood | adul | 2 | 2 | - | - | sr | - | 1 | 2008 |
| Hashemi, 2022 | 3rd | cau | grp | 8 | oth | 40 |  | clin | cut | adul | 2 | 1 | - | - | sr | - | 1 | 2022 |
| Hassiotis, 2013 | cbt | cau | ind | 12 | uk | 36 | 0.63 | oth | cut | oth | 2 | 1 | - | + | sr | + | 3 | 2013 |
| Hautzinger, 2004 | cbt | wl | grp | 12 | eu | 68,53 | 0.79 | com | mood | old | 2 | 3 | + | - | sr | + | 3 | 2004 |
| Hayman, 1980 | other psy | wl | grp | 8 | us | 21,3 | 1.00 | com | cut | adul | 2 | 1 | + | - | sr | - | 2 | 1980 |
| Heckman, 2013 - cet | other psy | cau | oth | 6 | us | 59 | 0.39 | oth | cut | med | 3 | 1 | + | - | sr | + | 3 | 2013 |
| Heckman, 2013 - segt | other psy | cau | oth | 7 | us | 59 | 0.39 | oth | cut | med | 3 | 1 | + | - | sr | + | 3 | 2013 |
| Heckman, 2017 | ipt | cau | tel | 8 | us | 51,9 | 0.37 | com | mood | med | 2 | 1 | + | - | sr | + | 3 | 2017 |
| Hemanny, 2019 - ba | bat | cau | ind | 12 | oth | 39,7 | 0.86 | com | mood | adul | 3 | 2 | + | - | sr | + | 3 | 2019 |
| Hemanny, 2019 - tbct | cbt | cau | ind | 12 | oth | 39,7 | 0.86 | com | mood | adul | 3 | 2 | + | - | sr | + | 3 | 2019 |
| Hermanns, 2015 | cbt | cau | grp | 5 | eu | 43,3 | 0.57 | oth | cut | med | 2 | 2 | - | + | sr | + | 3 | 2015 |
| Herrmann-Lingen, 2016 | dyn | cau | oth | 16 | eu | 59,2 | 0.21 | oth | cut | med | 2 | 1 | + | - | sr | + | 3 | 2016 |
| Hoifodt, 2013 | cbt | wl | gsh | 4 | eu | 36,1 | 0.73 | clin | cut | adul | 2 | 2 | + | - | sr | + | 3 | 2013 |
| Holden, 1989 | sup | cau | ind | 8 | uk | 26 | 1.00 | oth | mood | ppd | 2 | 1 | + | - | + | - | 2 | 1989 |
| Honey, 2002 | cbt | cau | grp | 8 | uk | 27,92 | 1.00 | oth | cut | ppd | 2 | 1 | - | - | sr | + | 2 | 2002 |
| Horrell, 2014 | cbt | wl | grp | 4 | uk | 44,1 | 0.8 | com | cut | adul | 2 | 1 | + | + | sr | + | 4 | 2014 |
| Hou, 2014 | cbt | cau | oth | 19 | eas | 28 | 1.00 | oth | mood | ppd | 2 | 1 | - | - | sr | - | 1 | 2014 |
| Hsiao, 2014 | other psy | cau | grp | 8 | eas |  | 0.71 | clin | cut | adul | 2 | 1 | + | + | sr | + | 4 | 2014 |
| Huang, 2016 | cbt | cau | grp | 12 | eas | 56,43 | 0.52 | oth | cut | med | 2 | 1 | - | - | sr | - | 1 | 2016 |
| Hum, 2019 | 3rd | wl | oth | 8 | can | 35,51 | 0.71 | com | cut | med | 2 | 1 | + | - | sr | + | 3 | 2019 |
| Hummel, 2017 | cbt | wl | grp | 13 | eu | 81,94 | 0.8 | oth | cut | med | 2 | 2 | + | - | + | + | 3 | 2017 |
| Hunter, 2012 | cbt | cau | grp | 18 | us | 35,16 | 0.48 | clin | cut | oth | 2 | 1 | - | - | sr | + | 2 | 2012 |
| Husain, 2017 | cbt | cau | grp | 6 | oth | 27,73 | 1.00 | oth | mood | ppd | 2 | 2 | - | + | + | + | 3 | 2017 |
| Husain, 2021a | cbt | cau | grp | 8 | oth |  | 1.00 | oth | mood | ppd | 2 | 2 | - | - | + | - | 1 | 2021 |
| Husain, 2021b | cbt | cau | grp | 10 | oth | 27 | 1.00 | oth | mood | ppd | 2 | 2 | + | - | - | - | 1 | 2021 |
| Jalali, 2019 | other psy | wl | grp | 11 | oth | 31,78 | 0.00 | oth | cut | oth | 2 | 1 | - | - | sr | - | 1 | 2019 |
| Jamison, 1995 | cbt | wl | gsh | 4 | us | 38 | 0.84 | com | mood | adul | 2 | 2 | - | - | sr | - | 1 | 1995 |
| Jesse, 2015 | cbt | cau | grp | 6 | us | 25,05 | 1.00 | oth | cut | ppd | 2 | 2 | + | + | sr | - | 3 | 2015 |
| Jiang, 2014 | other psy | cau | oth | 18 | eas | 27,3 | 1.00 | oth | cut | ppd | 2 | 1 | + | - | sr | - | 2 | 2014 |
| Johansson, 2012a | dyn | wl | gsh | 9 | eu | 45,6 | 0.75 | com | mood | adul | 2 | 3 | + | + | sr | + | 4 | 2012 |
| Johansson, 2012b - stand | cbt | wl | gsh | 6,46 | eu | 44,7 | 0.71 | com | mood | adul | 3 | 2 | + | + | sr | - | 3 | 2012 |
| Johansson, 2012b - tail | cbt | wl | gsh | 7,72 | eu | 44,7 | 0.71 | com | mood | adul | 3 | 2 | + | + | sr | - | 3 | 2012 |
| Johansson, 2019 | cbt | wl | gsh | 6 | eu | 39 | 0.57 | clin | mood | adul | 2 | 2 | + | + | sr | + | 4 | 2019 |
| Johnson, 2019 | ipt | cau | oth | 24 | us | 39 | 0.35 | oth | mood | oth | 2 | 2 | + | + | + | + | 4 | 2019 |
| Joling, 2011 | cbt | cau | gsh | 3 | eu | 81,45 | 0.74 | oth | cut | old | 2 | 1 | + | + | sr | + | 4 | 2011 |
| Jordans, 2019 | bat | cau | ind | 4 | oth |  | 0.85 | oth | mood | adul | 2 | 1 | + | + | sr | + | 4 | 2019 |
| Kamga, 2017 | cbt | wl | gsh | 8 | can | 76 | 0.63 | oth | cut | med | 2 | 1 | + | + | sr | + | 4 | 2017 |
| Kanter, 2015 | bat | cau | ind | 8 | us | 38,1 | 0.79 | clin | mood | oth | 2 | 2 | + | - | + | + | 3 | 2015 |
| Keeley, 2016 | other psy | cau | ind | 4 | us | 47,5 | 0.71 | clin | mood | adul | 2 | 1 | + | + | sr | + | 4 | 2016 |
| Kemmeren, 2023 | cbt | cau | oth | 19 | eu | 39,3 | 0.68 | clin | mood | adul | 2 | 2 | + | + | sr | + | 4 | 2023 |
| Kenter, 2016 | pst | wl | gsh | 5 | eu | 38 | 0.54 | clin | mood | adul | 2 | 1 | + | + | sr | + | 4 | 2016 |
| Kessler, 2009 | cbt | wl | oth | 10 | uk | 34,9 | 0.68 | clin | mood | adul | 2 | 1 | + | + | sr | + | 4 | 2009 |
| Khazraee, 2023 | 3rd | wl | ind | 8 | oth | 32,06 | 1.00 | clin | mood | adul | 2 | 1 | + | + | sr | - | 3 | 2023 |
| Khoshbooii, 2021 - grp | cbt | wl | grp | 16 | oth | 49 | 1.00 | oth | cut | oth | 3 | 1 | - | - | + | - | 1 | 2021 |
| Khoshbooii, 2021 - ind | cbt | wl | ind | 8 | oth | 49 | 1.00 | oth | cut | oth | 3 | 1 | - | - | + | - | 1 | 2021 |
| Kim, 2018 | cbt | cau | oth | 7 | eas | 48 | 1.00 | oth | cut | med | 2 | 2 | + | - | sr | - | 2 | 2018 |
| King, 2000 - cbt | cbt | cau | ind | 6 | uk | 37,5 | 0.77 | clin | cut | adul | 3 | 1 | - | + | sr | + | 3 | 2000 |
| King, 2000 - sup | sup | cau | ind | 6 | uk | 37,5 | 0.77 | clin | cut | adul | 3 | 1 | - | + | sr | + | 3 | 2000 |
| Kivi, 2014 | cbt | cau | gsh | 5 | eu | 36,6 | 0.57 | clin | mood | adul | 2 | 1 | - | + | sr | - | 2 | 2014 |
| Korrelboom, 2012 | other psy | wl | grp | 8 | eu | 40,9 | 0.8 | clin | mood | adul | 2 | 1 | + | + | sr | + | 4 | 2012 |
| Korte, 2012 | lrt | wl | grp | 8 | eu | 63,3 | 0.77 | com | cut | old | 2 | 1 | + | + | sr | + | 4 | 2012 |
| Kramer, 2014 | other psy | wl | oth | 5 | eu | 19,5 | 0.79 | com | cut | adol&yadul | 2 | 1 | + | + | sr | + | 4 | 2014 |
| Kramer, 2021 | cbt | wl | gsh | 4 | eu | 36,3 | 0.78 | clin | cut | adul | 2 | 1 | + | - | + | + | 3 | 2021 |
| Laidlaw, 2008 | cbt | cau | ind | 8 | uk | 74,03 | 0.73 | clin | mood | old | 2 | 3 | + | + | + | - | 3 | 2008 |
| Lamers, 2010 | cbt | cau | ind | 4 | eu | 70,7 | 0.47 | oth | mood | med | 2 | 1 | + | + | sr | + | 4 | 2010 |
| Lamers, 2015 | lrt | wl | gsh | 7 | eu | 56,94 | 0.77 | com | cut | adul | 2 | 1 | + | + | sr | + | 4 | 2015 |
| Landreville, 1997 | cbt | wl | gsh | 4 | can | 72 | 0.87 | com | cut | old | 2 | 3 | - | - | sr | - | 1 | 1997 |
| Lappalainen, 2015 | 3rd | wl | gsh | 6 | eu | 51,9 | 0.72 | com | mood | adul | 2 | 1 | - | + | sr | + | 3 | 2015 |
| Larcombe, 1984 | cbt | wl | grp | 6 | au | 42,5 | 0.68 | com | mood | med | 2 | 2 | - | - | sr | - | 1 | 1984 |
| Lee, 2021 | bat | cau | grp | 10 | eas | 37 | 0.58 | clin | mood | adul | 2 | 2 | + | + | + | + | 4 | 2021 |
| Lenze, 2017 | ipt | cau | ind | 8 | us | 26,64 | 1.00 | com | mood | ppd | 2 | 1 | + | + | sr | + | 4 | 2017 |
| Lenze, 2020 | ipt | cau | ind | 9 | us | 26 | 1.00 | com | mood | ppd | 2 | 1 | + | + | sr | + | 4 | 2020 |
| Lerner, 2015 | cbt | cau | tel | 8 | us | 54,7 | 0.72 | oth | mood | oth | 2 | 1 | + | - | sr | + | 3 | 2015 |
| Leung, 2013 | cbt | cau | grp | 6 | eas |  | 1.00 | oth | cut | ppd | 2 | 2 | - | - | sr | + | 2 | 2013 |
| Lexis, 2011 | pst | cau | ind | 8 | eu | 47,74 | 0.39 | oth | cut | oth | 2 | 2 | + | - | sr | + | 3 | 2011 |
| Liu, 2009 | cbt | wl | gsh | 8 | eas | 26,4 | 0.73 | com | cut | adul | 2 | 1 | - | - | sr | + | 2 | 2009 |
| Liu, 2021 | cbt | cau | ind | 6 | eas | 27 | 1.00 | oth | cut | ppd | 2 | 2 | + | + | + | - | 3 | 2021 |
| Lloyd-Williams, 2018 | other psy | cau | ind | 1 | uk | 65,1 | 0.71 | oth | cut | med | 2 | 1 | - | + | sr | + | 3 | 2018 |
| Lök, 2019 | lrt | cau | grp | 8 | oth |  | 0.57 | oth | cut | med | 2 | 1 | + | + | - | + | 3 | 2019 |
| Lovell, 2008 | cbt | cau | ind | 4 | uk | 37,6 | 0.74 | clin | cut | adul | 2 | 2 | - | + | sr | + | 3 | 2008 |
| Lund, 2020 | other psy | cau | ind | 6 | oth | 27 | 1.00 | oth | cut | ppd | 2 | 1 | + | + | + | + | 4 | 2020 |
| Lundgren, 2016 | cbt | wl | gsh | 4 | eu | 62,9 | 0.42 | oth | cut | med | 2 | 1 | + | + | sr | + | 4 | 2016 |
| Lustman, 1998 | cbt | cau | ind | 10 | us | 54,83 | 0.6 | com | mood | med | 2 | 1 | + | + | sr | + | 4 | 1998 |
| Lynch, 1997 | pst | cau | tel | 6 | us | 48,3 | 0.86 | clin | cut | adul | 2 | 1 | - | - | + | - | 1 | 1997 |
| Lynch, 2004 | pst | cau | tel | 6 | us | 38,5 | 0.83 | clin | cut | adul | 2 | 2 | - | - | sr | - | 1 | 2004 |
| Lynch, 2020 | other psy | cau | oth | 43 | uk |  | 0.66 | clin | mood | adul | 2 | 2 | + | + | + | + | 4 | 2020 |
| MacPherson, 2013 | sup | cau | ind | 8 | uk | 43,5 | 0.73 | clin | cut | adul | 2 | 2 | + | + | sr | + | 4 | 2013 |
| Mahmoodi, 2021 - cbt-p | cbt | wl | ind | 12 | oth | 27 | 0.53 | clin | cut | adul | 3 | 1 | + | + | + | - | 3 | 2021 |
| Mahmoodi, 2021 - up | cbt | wl | ind | 12 | oth | 27 | 0.53 | clin | cut | adul | 3 | 1 | + | + | + | - | 3 | 2021 |
| Maina, 2005 - dyn | dyn | wl | ind | 20 | eu | 36,8 | 0.63 | clin | mood | adul | 3 | 1 | - | - | + | + | 2 | 2005 |
| Maina, 2005 - sup | sup | wl | ind | 19 | eu | 36,8 | 0.63 | clin | mood | adul | 3 | 1 | - | - | + | + | 2 | 2005 |
| Malouff, 1988 - pst | pst | wl | grp | 4 | us | 39,08 | 0.66 | com | cut | oth | 3 | 2 | - | - | sr | - | 1 | 1988 |
| Malouff, 1988 - ret | cbt | wl | grp | 4 | us | 39,08 | 0.66 | com | cut | oth | 3 | 2 | - | - | sr | - | 1 | 1988 |
| Martin, 2015 | cbt | wl | ind | 12 | au | 40,64 | 0.74 | com | mood | med | 2 | 2 | + | + | sr | - | 3 | 2015 |
| Matsuzaka, 2017 | ipt | cau | ind | 4 | oth | 43,84 | 0.94 | clin | mood | adul | 2 | 2 | + | + | + | + | 4 | 2017 |
| McClay, 2015 | cbt | wl | grp | 4 | uk | 43,7 | 0.65 | com | cut | adul | 2 | 1 | - | + | sr | - | 2 | 2015 |
| McCusker, 2021 | cbt | wl | gsh | 8 | can | 58 | 0.79 | com | cut | med | 2 | 1 | + | - | + | + | 3 | 2021 |
| McIndoo, 2016 - bat | bat | wl | ind | 4 | us | 19,2 | 0.62 | com | cut | stud | 3 | 2 | + | - | - | + | 2 | 2016 |
| McIndoo, 2016 - mbt | 3rd | wl | ind | 4 | us | 19,2 | 0.62 | com | cut | stud | 3 | 2 | + | - | - | + | 2 | 2016 |
| McKee, 2006 | cbt | cau | ind | 5 | us | 24,42 | 1.00 | oth | cut | ppd | 2 | 1 | - | - | sr | - | 1 | 2006 |
| Mennen, 2021 | ipt | cau | grp | 12 | us | 33 | 1.00 | oth | cut | ppd | 2 | 1 | + | - | + | + | 3 | 2021 |
| Michalak, 2015 - cbasp | other psy | cau | oth | 10 | eu | 50,84 | 0.62 | com | mood | adul | 3 | 2 | + | + | sr | + | 4 | 2015 |
| Michalak, 2015 - mbct | 3rd | cau | grp | 8 | eu | 50,84 | 0.62 | com | mood | adul | 3 | 2 | + | + | sr | + | 4 | 2015 |
| Milgrom, 2005 - cbt | cbt | cau | grp | 9 | au | 29,7 | 1.00 | oth | mood | ppd | 4 | 1 | + | + | sr | + | 4 | 2005 |
| Milgrom, 2005 - grp sup | sup | cau | grp | 9 | au | 29,7 | 1.00 | oth | mood | ppd | 4 | 1 | + | + | sr | + | 4 | 2005 |
| Milgrom, 2005 - ind sup | sup | cau | ind | 9 | au | 29,7 | 1.00 | oth | mood | ppd | 4 | 1 | + | + | sr | + | 4 | 2005 |
| Milgrom, 2011 - nurse | cbt | cau | ind | 5 | au | 31,48 | 1.00 | oth | cut | ppd | 3 | 1 | + | + | sr | + | 4 | 2011 |
| Milgrom, 2011 - psych | cbt | cau | ind | 4 | au | 31,48 | 1.00 | oth | cut | ppd | 3 | 1 | + | + | sr | + | 4 | 2011 |
| Milgrom, 2015b | cbt | cau | ind | 6 | au | 31,79 | 1.00 | com | mood | ppd | 2 | 1 | + | + | sr | + | 4 | 2015 |
| Milgrom, 2016 | cbt | cau | gsh | 6 | au | 31,6 | 1.00 | com | mood | ppd | 2 | 1 | + | + | sr | + | 4 | 2016 |
| Milgrom, 2021 - ftf | cbt | cau | ind | 9 | au | 32,1 | 1.00 | com | mood | ppd | 3 | 1 | + | + | + | + | 4 | 2021 |
| Milgrom, 2021 - icbt | cbt | cau | gsh | 6 | au | 32,1 | 1.00 | com | mood | ppd | 3 | 1 | + | + | + | + | 4 | 2021 |
| Miller, 2002 | ipt | cau | tel | 12 | us | 32,1 | 1.00 | oth | cut | adul | 2 | 1 | - | - | + | + | 2 | 2002 |
| Miranda, 2003 | cbt | cau | oth | 8 | us | 29,3 | 1.00 | oth | mood | oth | 2 | 1 | + | + | + | + | 4 | 2003 |
| Mohr, 2000 | cbt | cau | tel | 8 | us | 42,35 | 0.72 | oth | cut | med | 2 | 1 | - | - | sr | + | 2 | 2000 |
| Mohr, 2011 | cbt | cau | tel | 16 | us | 55,9 | 0.09 | clin | mood | oth | 2 | 2 | - | - | + | + | 2 | 2011 |
| Mohr, 2013 | cbt | wl | gsh | 18 | us | 48,33 | 0.72 | com | mood | adul | 2 | 1 | + | + | sr | + | 4 | 2013 |
| Moldovan, 2013 - cau | cbt | cau | gsh | 4 | eu | 23 | 0.88 | com | cut | stud | 3 | 1 | + | + | sr | + | 4 | 2013 |
| Moldovan, 2013 - wl | cbt | wl | gsh | 4 | eu | 23 | 0.88 | com | cut | stud | 3 | 1 | + | + | sr | + | 4 | 2013 |
| Montero-Marin, 2016 | cbt | cau | gsh | 6 | eu | 42,93 | 0.76 | clin | mood | adul | 2 | 1 | + | + | sr | + | 4 | 2016 |
| Moon, 2021 | cbt | wl | grp | 8 | eas | 32,12 | 0.52 | oth | cut | med | 2 | 2 | + | + | + | + | 4 | 2021 |
| Mossey, 1996 | ipt | cau | ind | 10 | us | 71 | 0.78 | oth | cut | old | 2 | 1 | - | - | sr | - | 1 | 1996 |
| Mukhtar, 2011 | cbt | wl | grp | 8 | eas | 40,5 | 0.55 | clin | mood | adul | 2 | 1 | - | - | sr | - | 1 | 2011 |
| Mulcahy, 2010 | ipt | cau | oth | 11 | au | 32,22 | 1.00 | clin | mood | ppd | 2 | 3 | + | - | + | - | 2 | 2010 |
| Musa, 2021 | 3rd | cau | grp | 6 | oth |  | 0.61 | clin | mood | oth | 2 | 1 | + | + | + | + | 4 | 2021 |
| Nadort, 2022 | pst | cau | gsh | 5 | eu | 64 | 0.38 | oth | cut | med | 2 | 1 | + | + | sr | + | 3 | 2022 |
| Naeem, 2013 | cbt | cau | gsh | 9 | oth | 33,44 | 0.56 | clin | mood | adul | 2 | 1 | + | + | sr | + | 4 | 2013 |
| Naeem, 2015 | cbt | cau | oth | 7 | oth | 31,7 | 0.6 | clin | mood | adul | 2 | 1 | + | + | sr | + | 4 | 2015 |
| Nakagawa, 2017 | cbt | cau | ind | 15 | eas | 40,6 | 0.36 | clin | mood | adul | 2 | 3 | + | + | + | + | 4 | 2017 |
| Nasrin, 2017 | bat | wl | ind | 1 | uk | 36,31 | 0.59 | clin | mood | adul | 2 | 1 | + | + | sr | - | 3 | 2017 |
| Neugebauer, 2006 | ipt | cau | tel | 6 | us | 29,7 | 1.00 | oth | cut | oth | 2 | 1 | - | - | + | + | 2 | 2006 |
| Newby, 2013 | cbt | wl | gsh | 6 | au | 44,3 | 0.78 | com | cut | adul | 2 | 1 | + | + | sr | + | 4 | 2013 |
| Newby, 2014 | cbt | cau | ind | 1 | au | 26,28 | 0.44 | com | cut | adul | 2 | 1 | - | - | sr | + | 2 | 2014 |
| Newby, 2017 | cbt | wl | gsh | 6 | au | 46,7 | 0.71 | com | mood | med | 2 | 2 | + | + | sr | + | 4 | 2017 |
| Nezu, 1986 - pft | pst | wl | grp | 8 | us | 41,73 | 0.77 | com | mood | adul | 3 | 2 | - | - | sr | - | 1 | 1986 |
| Nezu, 1986 - pst | pst | wl | grp | 8 | us | 41,73 | 0.77 | com | mood | adul | 3 | 2 | - | - | sr | - | 1 | 1986 |
| Nezu, 1989 - abbr pst | pst | wl | grp | 10 | us | 41,73 | 0.77 | com | mood | adul | 3 | 2 | - | - | sr | - | 1 | 1989 |
| Nezu, 1989 - pst | pst | wl | grp | 10 | us | 41,73 | 0.77 | com | mood | adul | 3 | 2 | - | - | sr | - | 1 | 1989 |
| Ng, 2013 | lrt | cau | ind | 5 | eas |  |  | com | cut | old | 2 | 1 | - | - | sr | - | 1 | 2013 |
| Ngai, 2015 | cbt | cau | tel | 5 | eas | 30,8 | 1.00 | oth | cut | ppd | 2 | 1 | + | + | sr | + | 4 | 2015 |
| Niedermoser, 2020 | ipt | cau | grp | 8 | eu | 40,86 | 0.5 | com | mood | oth | 2 | 2 | + | + | - | + | 3 | 2020 |
| Nollett, 2016 - cau | pst | cau | ind | 6 | uk | 70,3 | 0.59 | oth | cut | med | 3 | 1 | + | + | sr | - | 3 | 2016 |
| Nollett, 2016 - wl | pst | wl | ind | 6 | uk | 70,3 | 0.59 | oth | cut | med | 3 | 1 | + | + | sr | - | 3 | 2016 |
| Noone, 2022 | 3rd | cau | grp | 7,3 | uk | 77,3 | 0.75 | oth | cut | med | 2 | 2 | + | + | sr | + | 4 | 2022 |
| Northwood, 2020 | other psy | cau | ind | 41 | us | 43,84 | 0.82 | clin | mood | oth | 2 | 1 | + | + | + | + | 4 | 2020 |
| Nyström, 2017 - i-ba-l | bat | wl | gsh | 8 | eu | 42 | 0.76 | com | mood | adul | 3 | 1 | + | + | sr | + | 4 | 2017 |
| Nyström, 2017 - i-ba-m | bat | wl | gsh | 8 | eu | 42 | 0.76 | com | mood | adul | 3 | 1 | + | + | sr | + | 4 | 2017 |
| O'Hara, 2000 | ipt | wl | ind | 12 | us | 29,55 | 1.00 | oth | mood | ppd | 2 | 2 | + | - | sr | + | 3 | 2000 |
| O'Mahen, 2013a | cbt | cau | ind | 8 | uk | 27,05 | 1.00 | oth | mood | ppd | 2 | 1 | + | + | sr | + | 4 | 2013 |
| O'Moore, 2018 | cbt | cau | gsh | 6 | au | 61,6 | 0.79 | oth | mood | med | 2 | 1 | - | + | sr | + | 3 | 2018 |
| O'Neil, 2014 | cbt | cau | tel | 8 | au | 59,96 | 0.25 | oth | cut | med | 2 | 2 | + | + | sr | + | 4 | 2014 |
| Olukolade, 2017 - cbt | cbt | wl | ind | 9 | oth |  | 0.57 | oth | cut | med | 3 | 1 | + | - | sr | - | 2 | 2017 |
| Olukolade, 2017 - edu | other psy | wl | ind | 9 | oth |  | 0.57 | oth | cut | med | 3 | 1 | + | - | sr | - | 2 | 2017 |
| Omidi, 2013 - cbt | cbt | cau | grp | 8 | oth | 32,33 | 0.67 | clin | mood | adul | 3 | 1 | - | - | sr | - | 1 | 2013 |
| Omidi, 2013 - mbct | 3rd | cau | grp | 8 | oth | 32,33 | 0.67 | clin | mood | adul | 3 | 1 | - | - | sr | - | 1 | 2013 |
| Onuigbo, 2019 | cbt | cau | grp | 14 | oth | 25,43 | 0.54 | oth | cut | med | 2 | 1 | + | + | sr | + | 4 | 2019 |
| Pace, 1993 | cbt | wl | ind | 7 | us | 22,13 | 0.78 | oth | cut | stud | 2 | 1 | - | - | sr | - | 1 | 1993 |
| Patel, 2017 | bat | cau | ind | 6 | oth | 42,5 | 0.77 | clin | cut | adul | 2 | 1 | + | + | sr | + | 4 | 2017 |
| Pecheur, 1984 - cbt | cbt | wl | ind | 8 | us | 24 | 0.9 | com | mood | stud | 3 | 2 | - | - | sr | - | 1 | 1984 |
| Pecheur, 1984 - rel cbt | cbt | wl | ind | 8 | us | 24 | 0.9 | com | mood | stud | 3 | 2 | - | - | sr | - | 1 | 1984 |
| Peden, 2000 | cbt | cau | grp | 6 | us | 19,3 | 1.00 | com | cut | stud | 2 | 1 | - | - | sr | - | 1 | 2000 |
| Pellas, 2022 | bat | wl | tel | 4 | eu | 75,6 | 0.83 | com | cut | old | 2 | 3 | + | + | sr | - | 3 | 2022 |
| Penckofer, 2012 | cbt | cau | grp | 8 | us | 54,41 | 1.00 | com | cut | med | 2 | 1 | + | - | sr | + | 3 | 2012 |
| Perini, 2009 | cbt | wl | gsh | 6 | au | 49,29 | 0.78 | com | mood | adul | 2 | 3 | + | - | sr | + | 3 | 2009 |
| Petersen, 2014 | ipt | cau | grp | 8 | oth | 36,5 | 0.74 | oth | mood | med | 2 | 1 | + | - | sr | - | 2 | 2014 |
| Pibernik-Okanovic, 2009 | cbt | cau | grp | 4 | eu |  |  | oth | cut | med | 2 | 1 | - | + | sr | + | 3 | 2009 |
| Piers, 2023 | cbt | wl | tel | 12 | us | 61,3 | 0.67 | com | mood | med | 2 | 1 | + | - | sr | - | 2 | 2023 |
| Pihlaja, 2020 | cbt | cau | gsh | 3,54 | eu | 37,4 | 0.68 | oth | mood | adul | 2 | 1 | - | - | sr | + | 2 | 2020 |
| Pinniger, 2012 | other psy | wl | grp | 6 | au | 44,39 | 0.91 | com | cut | adul | 2 | 1 | - | - | sr | - | 1 | 2012 |
| Poleshuck, 2014 | ipt | cau | ind | 4 | us | 36,7 | 1.00 | oth | mood | med | 2 | 2 | + | - | sr | + | 3 | 2014 |
| Pots, 2014 | 3rd | wl | grp | 11 | eu | 47,93 | 0.78 | com | cut | adul | 2 | 1 | + | + | sr | + | 4 | 2014 |
| Pots, 2016 | 3rd | wl | gsh | 9 | eu | 46,85 | 0.76 | com | cut | adul | 2 | 1 | + | + | sr | + | 4 | 2016 |
| Pott, 2022 | bat | cau | ind | 5,6 | uk | 42,3 | 0.27 | oth | cut | adul | 2 | 1 | + | + | sr | + | 4 | 2022 |
| Power, 2012 - cbt | cbt | wl | ind | 16 | uk | 36,1 | 0.62 | clin | mood | adul | 3 | 1 | - | - | sr | + | 2 | 2012 |
| Power, 2012 - ipt | ipt | wl | ind | 16 | uk | 36,1 | 0.62 | clin | mood | adul | 3 | 1 | - | - | sr | + | 2 | 2012 |
| Preschl, 2012 | lrt | wl | ind | 6 | eu | 70 | 0.67 | com | cut | old | 2 | 1 | + | - | sr | + | 3 | 2012 |
| Propst, 1992 - nrct-nt-cau | cbt | cau | ind | 19 | us | 40 | 0.83 | com | cut | oth | 5 | 2 | - | - | sr | - | 1 | 1992 |
| Propst, 1992 - nrct-nt-wl | cbt | wl | ind | 19 | us | 40 | 0.83 | com | cut | oth | 5 | 2 | - | - | sr | - | 1 | 1992 |
| Propst, 1992 - nrct-rt-cau | cbt | cau | ind | 19 | us | 40 | 0.83 | com | cut | oth | 5 | 2 | - | - | sr | - | 1 | 1992 |
| Propst, 1992 - nrct-rt-wl | cbt | wl | ind | 19 | us | 40 | 0.83 | com | cut | oth | 5 | 2 | - | - | sr | - | 1 | 1992 |
| Propst, 1992 - rct-nt-cau | cbt | cau | ind | 19 | us | 40 | 0.83 | com | cut | oth | 5 | 2 | - | - | sr | - | 1 | 1992 |
| Propst, 1992 - rct-nt-wl | cbt | wl | ind | 19 | us | 40 | 0.83 | com | cut | oth | 5 | 2 | - | - | sr | - | 1 | 1992 |
| Propst, 1992 - rct-rt-cau | cbt | cau | ind | 19 | us | 40 | 0.83 | com | cut | oth | 5 | 2 | - | - | sr | - | 1 | 1992 |
| Propst, 1992 - rct-rt-wl | cbt | wl | ind | 19 | us | 40 | 0.83 | com | cut | oth | 5 | 2 | - | - | sr | - | 1 | 1992 |
| Psarraki, 2021 | other psy | cau | oth | 8 | eu | 47,49 | 0.84 | clin | mood | adul | 2 | 2 | + | - | + | - | 2 | 2021 |
| Puckering, 2010 | other psy | wl | grp | 14 | uk |  | 1.00 | oth | cut | ppd | 2 | 1 | + | - | sr | - | 2 | 2010 |
| Pugh, 2016 | cbt | wl | gsh | 6 | can | 30,82 | 1.00 | com | cut | ppd | 2 | 2 | + | + | sr | - | 3 | 2016 |
| Qiu, 2013 | cbt | wl | grp | 10 | eas | 50,63 | 1.00 | oth | mood | med | 2 | 1 | + | + | + | + | 4 | 2013 |
| Raevuori, 2021 | other psy | cau | gsh | 8 | eu | 25,1 | 0.73 | clin | mood | stud | 2 | 1 | + | + | + | + | 4 | 2021 |
| Ransom, 2008 | ipt | cau | tel | 6 | us | 44,4 | 0.10 | com | mood | med | 2 | 1 | - | - | sr | + | 2 | 2008 |
| Raue, 2019 | bat | cau | ind | 12 | us | 76 | 0.83 | oth | cut | old | 2 | 1 | - | - | - | - | 0 | 2019 |
| Raya-Tena, 2021 | other psy | cau | grp | 12 | eu | 68,4 | 0.82 | clin | cut | med | 2 | 1 | + | - | + | + | 3 | 2021 |
| Rehm, 1981 - sm | other psy | wl | grp | 7 | us | 39,2 | 1.00 | com | cut | adul | 5 | 3 | - | - | sr | - | 1 | 1981 |
| Rehm, 1981 - sm+se | other psy | wl | grp | 7 | us | 39,2 | 1.00 | com | cut | adul | 5 | 3 | - | - | sr | - | 1 | 1981 |
| Rehm, 1981 - sm+se+sr | other psy | wl | grp | 7 | us | 39,2 | 1.00 | com | cut | adul | 5 | 3 | - | - | sr | - | 1 | 1981 |
| Rehm, 1981 - sm+sr | other psy | wl | grp | 7 | us | 39,2 | 1.00 | com | cut | adul | 5 | 3 | - | - | sr | - | 1 | 1981 |
| Richards, 2015 | cbt | wl | gsh | 7 | eu | 39,86 | 0.73 | com | cut | adul | 2 | 1 | + | + | sr | + | 4 | 2015 |
| Richards, 2018 | bat | cau | ind | 8 | uk | 65,3 | 0.48 | oth | cut | med | 2 | 1 | + | + | sr | + | 4 | 2018 |
| Rief, 2018 - cbasp | other psy | wl | ind | 16 | eu | 37,5 | 0.51 | clin | mood | adul | 4 | 1 | + | + | sr | + | 4 | 2018 |
| Rief, 2018 - cbt-e | cbt | wl | ind | 16 | eu | 37,5 | 0.51 | clin | mood | adul | 4 | 1 | + | + | sr | + | 4 | 2018 |
| Rief, 2018 - cbt-m | cbt | wl | ind | 16 | eu | 37,5 | 0.51 | clin | mood | adul | 4 | 1 | + | + | sr | + | 4 | 2018 |
| Rizvi, 2015 | cbt | wl | ind | 14 | can | 42,35 | 0.74 | clin | mood | adul | 2 | 1 | - | - | sr | - | 1 | 2015 |
| Rohan, 2007 | cbt | wl | grp | 10 | us | 45 | 0.9 | com | mood | adul | 2 | 3 | + | - | + | + | 3 | 2007 |
| Rohricht, 2013 | other psy | wl | grp | 8 | uk | 47,7 | 0.42 | clin | mood | adul | 2 | 1 | + | + | + | + | 4 | 2013 |
| Ross, 1985 | cbt | wl | oth | 12 | uk | 33 | 0.63 | clin | mood | adul | 2 | 2 | - | - | sr | - | 1 | 1985 |
| Rude, 1986 | other psy | wl | grp | 12 | us | 40 | 1.00 | com | cut | adul | 2 | 1 | - | - | sr | - | 1 | 1986 |
| Russell, 2020 | bat | cau | gsh | 8 | uk | 37,71 | 0.27 | oth | cut | oth | 2 | 3 | + | + | + | - | 3 | 2020 |
| Ruwaard, 2009 | cbt | wl | gsh | 8 | eu | 42 | 0.69 | com | cut | adul | 2 | 3 | + | - | sr | + | 3 | 2009 |
| Ruzickova, 2021 | bat | cau | tel | 4 | uk | 31,59 | 0.84 | com | cut | adul | 2 | 1 | - | - | + | - | 1 | 2021 |
| Safren, 2009 | cbt | wl | ind | 10 | us |  | 0.10 | com | mood | med | 2 | 2 | - | - | sr | + | 2 | 2009 |
| Safren, 2014 | cbt | cau | ind | 10 | us | 56,83 | 0.49 | oth | mood | med | 2 | 1 | + | + | + | - | 3 | 2014 |
| Safren, 2016 - cbt | cbt | cau | ind | 11 | us | 47,45 | 0.31 | com | mood | med | 3 | 2 | + | - | sr | + | 3 | 2016 |
| Safren, 2016 - sup | sup | cau | ind | 11 | us | 47,45 | 0.31 | com | mood | med | 3 | 2 | + | - | sr | + | 3 | 2016 |
| Safren, 2021 | cbt | cau | ind | 8 | oth |  | 0.7 | oth | mood | med | 2 | 2 | + | - | + | + | 3 | 2021 |
| Saisanan, 2020 | bat | cau | grp | 10,8 | oth | 69,9 | 0.79 | oth | cut | old | 2 | 2 | - | - | sr | + | 2 | 2022 |
| Salamanca-Sanabria, 2020 | cbt | wl | gsh | 7 | oth | 22,2 | 0.69 | oth | cut | stud | 2 | 1 | + | + | sr | - | 3 | 2020 |
| Saloheimo, 2016 - cbt | cbt | cau | grp | 11 | eu | 41,66 | 0.72 | clin | mood | adul | 3 | 1 | + | + | + | - | 3 | 2016 |
| Saloheimo, 2016 - ipt | ipt | cau | ind | 13 | eu | 41,66 | 0.72 | clin | mood | adul | 3 | 1 | + | + | + | - | 3 | 2016 |
| Sander, 2020 | cbt | cau | gsh | 6 | eu | 51,7 | 0.6 | oth | cut | med | 2 | 1 | + | + | sr | + | 4 | 2020 |
| Savard, 2006 | cbt | wl | ind | 8 | can | 51,55 | 1.00 | com | cut | med | 2 | 3 | + | + | sr | - | 3 | 2006 |
| Savari, 2021 | 3rd | wl | grp | 8 | oth | 24,3 | 1.00 | clin | mood | stud | 2 | 1 | + | - | + | + | 3 | 2021 |
| Scazufca, 2022 | bat | cau | ind | 9,5 | oth |  | 0.74 | oth | cut | old | 2 | 1 | + | + | sr | + | 4 | 2022 |
| Schlicker, 2020 | cbt | wl | gsh | 4,8 | eu | 51,3 | 0.65 | oth | mood | med | 2 | 2 | + | + | sr | + | 4 | 2020 |
| Schmidt, 1983 - gsh | cbt | wl | gsh | 6 | us | 42 | 0.84 | com | cut | adul | 5 | 5 | - | - | sr | - | 1 | 1983 |
| Schmidt, 1983 - ind | cbt | wl | ind | 7 | us | 42 | 0.84 | com | cut | adul | 5 | 5 | - | - | sr | - | 1 | 1983 |
| Schmidt, 1983 - large grp | cbt | wl | grp | 7 | us | 42 | 0.84 | com | cut | adul | 5 | 5 | - | - | sr | - | 1 | 1983 |
| Schmidt, 1983 - small grp | cbt | wl | grp | 7 | us | 42 | 0.84 | com | cut | adul | 5 | 5 | - | - | sr | - | 1 | 1983 |
| Schramm, 2020 | ipt | cau | grp | 8 | eu | 47,4 | 0.79 | clin | mood | oth | 2 | 1 | + | - | sr | + | 3 | 2020 |
| Schulberg, 1996 | ipt | cau | ind | 16 | us | 38,09 | 0.83 | clin | mood | adul | 2 | 1 | - | - | + | + | 2 | 1996 |
| Schuster, 2017 | other psy | wl | grp | 8 | eu | 45,9 | 0.68 | com | cut | adul | 2 | 1 | + | - | sr | + | 3 | 2017 |
| Scogin, 1987 | cbt | wl | gsh | 4 | us | 70,54 | 0.79 | com | cut | old | 2 | 3 | - | - | sr | - | 1 | 1987 |
| Scogin, 1989 - behav | cbt | wl | gsh | 4 | us | 68,34 | 0.85 | com | cut | old | 3 | 2 | - | - | - | - | 0 | 1989 |
| Scogin, 1989 - cogn | cbt | wl | gsh | 4 | us | 68,34 | 0.85 | com | cut | old | 3 | 2 | - | - | - | - | 0 | 1989 |
| Scott, 1990 - grp | cbt | wl | grp | 12 | uk | 33 | 0.7 | clin | mood | adul | 3 | 1 | - | - | sr | + | 2 | 1990 |
| Scott, 1990 - ind | cbt | wl | ind | 12 | uk | 33 | 0.7 | clin | mood | adul | 3 | 1 | - | - | sr | + | 2 | 1990 |
| Scott, 1992 - cbt | cbt | cau | ind | 16 | uk | 31,79 | 0.75 | clin | mood | adul | 3 | 1 | - | + | + | + | 3 | 1992 |
| Scott, 1992 - sup | sup | cau | ind | 16 | uk | 31,79 | 0.75 | clin | mood | adul | 3 | 1 | - | + | + | + | 3 | 1992 |
| Scott, 1997 | cbt | cau | ind | 6 | uk | 41 | 0.67 | clin | mood | adul | 2 | 2 | - | - | sr | - | 1 | 1997 |
| Segre, 2015 | other psy | wl | ind | 5 | us | 26,3 | 1.00 | oth | cut | oth | 2 | 3 | + | - | + | + | 3 | 2015 |
| Selmi, 1990 - ftf | cbt | wl | ind | 6 | us | 28,2 | 0.64 | com | mood | adul | 3 | 3 | - | - | sr | + | 2 | 1990 |
| Selmi, 1990 - gsh | cbt | wl | gsh | 6 | us | 28,2 | 0.64 | com | mood | adul | 3 | 3 | - | - | sr | + | 2 | 1990 |
| Serfaty, 2009 | cbt | cau | ind | 7 | uk | 74,1 | 0.79 | com | mood | old | 2 | 1 | + | + | sr | + | 4 | 2009 |
| Serfaty, 2020 | cbt | cau | ind | 5 | uk | 59,5 | 0.66 | oth | mood | med | 2 | 2 | + | + | sr | + | 4 | 2020 |
| Serrano, 2004 | lrt | cau | ind | 4 | eu | 77,19 | 0.77 | oth | cut | old | 2 | 1 | - | - | sr | - | 1 | 2004 |
| Shan, 2022 | cbt | cau | ind | 10 | eas | 62,81 | 0.64 | oth | mood | med | 2 | 2 | + | + | + | - | 3 | 2022 |
| Shaw, 1977 - bat | bat | wl | grp | 8 | can | 20,08 | 0.69 | com | cut | stud | 3 | 1 | - | - | sr | - | 1 | 1977 |
| Shaw, 1977 - cbt | cbt | wl | grp | 8 | can | 20,08 | 0.69 | com | cut | stud | 3 | 1 | - | - | sr | - | 1 | 1977 |
| Sheeber, 2012 | cbt | wl | gsh | 6 | us | 31 | 1.00 | oth | cut | ppd | 2 | 1 | + | + | sr | + | 4 | 2012 |
| Sikander, 2019 | cbt | cau | oth | 11 | oth | 27 | 1.00 | oth | cut | ppd | 2 | 1 | + | + | sr | + | 4 | 2019 |
| Simoni, 2013 | cbt | cau | ind | 11 | us | 46 | 0.28 | com | cut | med | 2 | 2 | + | + | sr | + | 4 | 2013 |
| Simpson, 2003 | dyn | cau | ind | 5 | uk | 42,99 | 0.8 | clin | mood | adul | 2 | 1 | + | + | sr | + | 4 | 2003 |
| Simson, 2008 | sup | cau | ind | 5 | eu | 60,5 | 0.43 | oth | cut | med | 2 | 1 | - | - | sr | + | 2 | 2008 |
| Sinniah, 2017 | cbt | cau | ind | 16 | eas | 43,13 | 0.7 | clin | mood | oth | 2 | 2 | - | - | sr | + | 2 | 2017 |
| Sirey, 2021 | other psy | cau | ind | 9 | us | 70,75 | 0.8 | oth | cut | oth | 2 | 1 | - | - | + | + | 2 | 2021 |
| Smit, 2006 | cbt | cau | ind | 14 | eu | 42,79 | 0.64 | clin | mood | adul | 2 | 1 | + | + | sr | + | 4 | 2006 |
| Smith, 2017 | cbt | wl | gsh | 6 | au | 39,91 | 0.82 | com | mood | adul | 3 | 2 | + | + | sr | + | 4 | 2017 |
| Songprakun, 2012 | cbt | cau | gsh | 8 | eas | 42,13 | 0.73 | clin | mood | adul | 2 | 1 | + | + | sr | + | 4 | 2012 |
| Spek, 2007 | cbt | wl | grp | 9 | eu | 55 | 0.63 | com | cut | old | 2 | 1 | + | + | sr | + | 4 | 2007 |
| Spruill, 2021 | other psy | cau | oth | 8 | us | 43,3 | 0.71 | oth | cut | med | 2 | 1 | + | + | + | + | 4 | 2021 |
| Sreevani, 2013 | other psy | cau | grp | 4 | oth | 28 | 0.6 | clin | mood | adul | 2 | 1 | + | - | sr | - | 2 | 2013 |
| Steel, 2020 | other psy | cau | ind | 8 | uk | 43 | 0.25 | clin | cut | adul | 2 | 1 | + | + | sr | + | 4 | 2020 |
| Stiles-Shields, 2018 - bat | bat | wl | gsh | 6 | us |  |  | com | cut | adul | 3 | 1 | + | + | sr | - | 3 | 2018 |
| Stiles-Shields, 2018 - cbt | cbt | wl | gsh | 6 | us |  |  | com | cut | adul | 3 | 1 | + | + | sr | - | 3 | 2018 |
| Strauss, 2012 | cbt | cau | grp | 9 | uk | 43 | 0.71 | clin | mood | adul | 2 | 1 | - | - | sr | + | 2 | 2012 |
| Strong, 2008 | pst | cau | ind | 10 | uk | 56,6 | 0.71 | oth | mood | med | 2 | 1 | + | + | sr | + | 4 | 2008 |
| Stuart, 2022 | cbt | cau | gsh | 6 | us | 47 | 0.81 | oth | cut | adul | 2 | 1 | + | + | sr | + | 2 | 2022 |
| Sugg, 2018 | other psy | cau | ind | 8 | uk | 49,2 | 0.61 | com | mood | adul | 2 | 1 | + | + | sr | + | 4 | 2018 |
| Sun, 2022 | bat | cau | ind | 6 | eas | 62,03 | 0.43 | oth | cut | med | 2 | 2 | + | + | - | - | 2 | 2022 |
| Swartz, 2008 | ipt | cau | ind | 9 | us | 42,7 | 1.00 | com | mood | oth | 2 | 2 | - | - | sr | - | 1 | 2008 |
| Szumska, 2020 | 3rd | wl | grp | 8 | eu | 32,4 | 0.67 | com | mood | adul | 2 | 1 | - | - | sr | - | 1 | 2020 |
| Takagaki, 2016 | bat | cau | grp | 5 | eas | 18,22 | 0.38 | oth | cut | stud | 2 | 1 | + | + | sr | + | 4 | 2016 |
| Talbot, 2011 | ipt | cau | ind | 13 | us | 36 | 1.00 | clin | mood | oth | 2 | 2 | - | - | - | + | 1 | 2011 |
| Taylor, 1977 - bat | bat | wl | ind | 6 | can | 22,4 | 0.71 | com | cut | stud | 4 | 2 | - | - | sr | - | 1 | 1977 |
| Taylor, 1977 - cbt | cbt | wl | ind | 6 | can | 22,4 | 0.71 | com | cut | stud | 4 | 2 | - | - | sr | - | 1 | 1977 |
| Taylor, 1977 - ct | cbt | wl | ind | 6 | can | 22,4 | 0.71 | com | cut | stud | 4 | 2 | - | - | sr | - | 1 | 1977 |
| Taylor, 2009 | cbt | wl | ind | 15 | us | 62,24 | 0.67 | com | cut | med | 2 | 2 | - | - | sr | + | 2 | 2009 |
| Teasdale, 1984 | cbt | cau | ind | 15 | uk | 37,5 | 0.94 | clin | mood | adul | 2 | 1 | - | - | sr | - | 1 | 1984 |
| Teichman, 1995 - ind | cbt | wl | ind | 13 | oth | 47,86 | 0.53 | clin | mood | adul | 3 | 1 | - | - | sr | - | 1 | 1995 |
| Teichman, 1995 - mar | cbt | wl | oth | 13 | oth | 47,86 | 0.53 | clin | mood | adul | 3 | 1 | - | - | sr | - | 1 | 1995 |
| Teri, 1997 - bat | bat | wl | oth | 9 | us | 76,4 | 0.47 | oth | mood | med | 3 | 2 | - | - | sr | - | 1 | 1997 |
| Teri, 1997 - pst | pst | wl | oth | 9 | us | 76,4 | 0.47 | oth | mood | med | 3 | 2 | - | - | sr | - | 1 | 1997 |
| Thomas, 2019 | bat | cau | ind | 8 | uk | 65,6 | 0.4 | com | cut | med | 2 | 2 | + | + | sr | + | 4 | 2019 |
| Titov, 2010 - clin | cbt | wl | gsh | 6 | au | 43 | 0.74 | com | mood | adul | 3 | 4 | + | - | sr | + | 3 | 2010 |
| Titov, 2010 - techn | cbt | wl | gsh | 6 | au | 43 | 0.74 | com | mood | adul | 3 | 4 | + | - | sr | + | 3 | 2010 |
| Titov, 2015a | cbt | wl | gsh | 5 | au | 65,31 | 0.73 | com | cut | old | 2 | 1 | + | + | sr | + | 4 | 2015 |
| Tomasino, 2017 - icbt_peer | cbt | wl | gsh | 11 | us | 69,6 | 0.68 | com | cut | old | 3 | 1 | - | - | sr | - | 1 | 2017 |
| Tomasino, 2017 -icbt | cbt | wl | gsh | 12 | us | 69,6 | 0.68 | com | cut | old | 3 | 1 | - | - | sr | - | 1 | 2017 |
| Tong, 2020 | cbt | wl | grp | 8 | eas | 37,7 | 0.72 | clin | mood | adul | 2 | 1 | + | - | + | - | 2 | 2020 |
| Tovote, 2014 - cbt | cbt | wl | ind | 8 | eu | 53,1 | 0.49 | oth | cut | med | 3 | 2 | + | - | - | + | 2 | 2014 |
| Tovote, 2014 - mbct | 3rd | wl | ind | 8 | eu | 53,1 | 0.49 | oth | cut | med | 3 | 2 | + | - | - | + | 2 | 2014 |
| Town, 2017 | dyn | cau | ind | 16 | can | 41,55 | 0.63 | clin | mood | adul | 2 | 2 | + | + | + | + | 4 | 2017 |
| Trevillion, 2020 | cbt | cau | oth | 6,5 | uk |  | 1.00 | com | mood | ppd | 2 | 1 | + | + | sr | + | 4 | 2020 |
| Tulbure, 2018 - c-cbt | cbt | wl | gsh | 9 | eu | 32,05 | 0.82 | com | mood | adul | 3 | 2 | - | - | sr | + | 2 | 2018 |
| Tulbure, 2018 - r-cbt | cbt | wl | gsh | 9 | eu | 32,05 | 0.82 | com | mood | adul | 3 | 2 | - | - | sr | + | 2 | 2018 |
| Unlu-Ince, 2013 | pst | wl | gsh | 5 | eu | 35,2 | 0.61 | com | cut | oth | 2 | 1 | + | - | sr | + | 3 | 2013 |
| van Bastelaar, 2011 | cbt | wl | gsh | 8 | eu | 50 | 0.61 | com | cut | med | 2 | 1 | + | + | sr | + | 4 | 2011 |
| van der Zanden, 2012 | cbt | wl | oth | 6 | eu | 20,9 | 0.84 | com | cut | adol&yadul | 2 | 1 | + | + | sr | + | 4 | 2012 |
| Van Horne, 2022 | pst | cau | ind | 5 | us | 30,2 | 1.00 | oth | cut | ppd | 2 | 1 | - | + | sr | - | 2 | 2022 |
| van Lieshout, 2021 | cbt | wl | oth | 1 | can | 31,8 | 1.00 | com | cut | ppd | 2 | 1 | + | + | + | + | 4 | 2021 |
| Van Lieshout, 2022 | cbt | cau | grp | 7 | can | 30,9 | 1.00 | com | cut | ppd | 2 | 1 | + | + | sr | + | 4 | 2022 |
| Van Luenen, 2018 | cbt | wl | gsh | 8 | eu | 46,3 | 0.10 | oth | cut | med | 2 | 2 | + | + | sr | + | 4 | 2018 |
| van Schaik, 2006 | ipt | cau | ind | 8 | eu | 67,93 | 0.69 | clin | mood | old | 2 | 2 | + | - | sr | + | 3 | 2006 |
| Vazquez-Gonzalez, 2013 | pst | cau | grp | 5 | eu | 53,9 | 1.00 | oth | cut | oth | 2 | 1 | - | + | sr | + | 3 | 2013 |
| Vazquez, 2017 - bat | bat | cau | oth | 4 | eu | 58,4 | 0.93 | oth | cut | oth | 3 | 1 | + | + | sr | + | 4 | 2017 |
| Vazquez, 2017 - cbt | cbt | cau | oth | 5 | eu | 58,4 | 0.93 | oth | cut | oth | 3 | 1 | + | + | sr | + | 4 | 2017 |
| Vazquez, 2020 - bat | bat | cau | oth | 4 | eu | 54,5 | 0.96 | oth | cut | oth | 3 | 1 | + | + | sr | + | 4 | 2020 |
| Vazquez, 2020 - cbt | cbt | cau | oth | 4,4 | eu | 54,8 | 0.9 | oth | cut | oth | 3 | 1 | + | + | sr | + | 4 | 2020 |
| Verduyn, 2003 - cbt | cbt | cau | grp | 16 | uk | 29,81 | 1.00 | oth | cut | oth | 3 | 2 | - | + | sr | - | 2 | 2003 |
| Verduyn, 2003 - sup | sup | cau | grp | 16 | uk | 29,81 | 1.00 | oth | cut | oth | 3 | 2 | - | + | sr | - | 2 | 2003 |
| Vernmark, 2010 - email | cbt | wl | gsh | 8 | eu | 36,82 | 0.68 | com | mood | adul | 3 | 2 | + | + | sr | + | 4 | 2010 |
| Vernmark, 2010 - guided | cbt | wl | gsh | 6 | eu | 36,82 | 0.68 | com | mood | adul | 3 | 2 | + | + | sr | + | 4 | 2010 |
| Vigod, 2021 | ipt | wl | grp | 10 | can | 33 | 1.00 | com | cut | ppd | 2 | 1 | + | - | + | + | 3 | 2021 |
| Vitriol, 2009 | dyn | cau | ind | 12 | oth | 38,86 | 1.00 | clin | mood | oth | 2 | 1 | - | - | + | + | 2 | 2009 |
| Warmerdam, 2008 - cbt | cbt | wl | gsh | 8 | eu | 45 | 0.71 | com | cut | adul | 3 | 1 | + | + | sr | + | 4 | 2008 |
| Warmerdam, 2008 - pst | pst | wl | gsh | 5 | eu | 45 | 0.71 | com | cut | adul | 3 | 1 | + | + | sr | + | 4 | 2008 |
| Watkins, 2012 | other psy | cau | oth | 7 | uk | 43,6 | 0.64 | clin | mood | adul | 2 | 3 | + | + | + | + | 4 | 2012 |
| Westerhof, 2019 - couns | lrt | wl | gsh | 5 | eu | 53,8 | 0.78 | com | cut | adul | 3 | 1 | + | + | sr | + | 4 | 2019 |
| Westerhof, 2019 - peer | lrt | wl | oth | 4 | eu | 53,8 | 0.78 | com | cut | adul | 3 | 1 | + | + | sr | + | 4 | 2019 |
| Wickberg, 1996 | sup | cau | ind | 6 | eu | 28,4 | 1.00 | oth | mood | ppd | 2 | 1 | - | - | + | - | 1 | 1996 |
| Wiersma, 2014 | other psy | cau | ind | 24 | eu | 41,6 | 0.6 | clin | mood | adul | 2 | 1 | + | + | sr | + | 4 | 2014 |
| Wiklund, 2010 | cbt | cau | ind | 3 | eu |  | 1.00 | oth | cut | ppd | 2 | 1 | - | - | sr | - | 1 | 2010 |
| Williams, 2013a | cbt | wl | gsh | 13 | au | 44,76 | 0.76 | com | mood | adul | 2 | 3 | + | + | sr | + | 4 | 2013 |
| Williams, 2013b | cbt | cau | gsh | 2 | uk | 41,75 | 0.68 | clin | cut | adul | 2 | 1 | - | + | sr | + | 3 | 2013 |
| Williams, 2018 | cbt | wl | grp | 8 | uk | 46,6 | 0.68 | com | cut | adul | 2 | 2 | + | + | sr | + | 4 | 2018 |
| Wilson, 1983 - bat | bat | wl | ind | 8 | au | 39,5 | 0.8 | com | cut | adul | 3 | 2 | - | - | sr | - | 1 | 1983 |
| Wilson, 1983 - cbt | cbt | wl | ind | 8 | au | 39,5 | 0.8 | com | cut | adul | 3 | 2 | - | - | sr | - | 1 | 1983 |
| Wollersheim, 1991 - cbt | cbt | wl | grp | 10 | us | 39,4 | 0.72 | com | mood | adul | 4 | 2 | - | - | sr | - | 1 | 1991 |
| Wollersheim, 1991 - gsh | cbt | wl | gsh | 10 | us | 39,4 | 0.72 | com | mood | adul | 4 | 2 | - | - | sr | - | 1 | 1991 |
| Wollersheim, 1991 - sup | sup | wl | grp | 10 | us | 39,4 | 0.72 | com | mood | adul | 4 | 2 | - | - | sr | - | 1 | 1991 |
| Wong, 2008a | cbt | wl | grp | 10 | eas | 42,72 | 0.78 | com | cut | adul | 2 | 1 | - | + | sr | + | 3 | 2008 |
| Wong, 2008b | cbt | wl | grp | 10 | eas | 37,4 | 0.78 | com | mood | adul | 2 | 1 | - | + | sr | - | 2 | 2008 |
| Wong, 2018 | 3rd | cau | grp | 8 | eas | 54 | 0.93 | clin | cut | adul | 2 | 1 | + | + | sr | + | 4 | 2018 |
| Wright, 2005 - c-cbt | cbt | wl | gsh | 9 | us | 40,23 | 0.76 | com | mood | adul | 3 | 2 | - | - | sr | - | 1 | 2005 |
| Wright, 2005 - cbt | cbt | wl | ind | 9 | us | 40,23 | 0.76 | com | mood | adul | 3 | 2 | - | - | sr | - | 1 | 2005 |
| Wright, 2022 | cbt | cau | gsh | 9 | us | 47 | 0.85 | clin | cut | adul | 2 | 1 | + | + | sr | + | 3 | 2022 |
| Wuthrich, 2013 | cbt | wl | grp | 9 | au | 67,44 | 0.65 | com | mood | old | 2 | 3 | + | - | sr | + | 3 | 2013 |
| Xie, 2019 | bat | cau | grp | 8 | eas | 71,9 | 0.59 | oth | cut | old | 2 | 1 | + | - | sr | - | 2 | 2019 |
| Yang, 2018 | other psy | cau | grp | 11 | eas | 18,5 | 0.6 | oth | mood | stud | 2 | 1 | + | - | sr | - | 2 | 2018 |
| Yeung, 2017 | cbt | wl | gsh | 5 | eas | 33 | 0.77 | clin | cut | adul | 2 | 1 | + | + | sr | - | 3 | 2017 |
| Ying, 2022 - ftf | cbt | wl | grp | 5 | eas | 41,3 | 0.66 | com | cut | adul | 3 | 3 | + | + | sr | + | 4 | 2022 |
| Ying, 2022 - icbt | cbt | wl | gsh | 5 | eas | 41,3 | 0.66 | com | cut | adul | 3 | 3 | + | + | sr | + | 4 | 2022 |
| Yuan, 2020 | cbt | cau | ind | 8 | eas | 70,5 | 0.38 | oth | cut | old | 2 | 1 | + | + | sr | + | 4 | 2020 |
| Zemestani, 2016 - bat | bat | wl | grp | 8 | oth | 24,2 | 0.61 | oth | mood | stud | 3 | 1 | + | - | 2 | + | 3 | 2016 |
| Zemestani, 2016 - mct | 3rd | wl | grp | 8 | oth | 24,2 | 0.61 | oth | mood | stud | 3 | 1 | + | - | 2 | + | 3 | 2016 |
| Zemestani, 2020b | 3rd | cau | grp | 8 | oth | 29,6 | 1.00 | oth | mood | ppd | 2 | 1 | + | + | 2 | + | 4 | 2019 |
| Zhao, 2019 | other psy | cau | grp | 4 | eas | 30,52 | 1.00 | oth | cut | med | 2 | 2 | + | - | sr | - | 2 | 2019 |
| Zhao, 2021 | other psy | cau | ind | 4 | eas |  | 1.00 | oth | cut | ppd | 2 | 1 | + | - | + | + | 3 | 2021 |
| Zu, 2014 | cbt | cau | ind | 20 | eas | 38,54 | 0.51 | clin | mood | adul | 2 | 2 | - | - | + | - | 1 | 2014 |

Abbreviations: 3rd: third wave therapy; Ac: allocation concealment; adol&yadul: adolescents and young adults; adul: adults; au: Australia; Ba: blind assessment; Bat: behavioral activation therapy; C: country; Can: Canada; Cbt: cognitive behavior therapy; Clin: clinical recruitment; Com: recruitment from the community; Ctr: control; Cut: scoring above a cut-off; Dx: diagnosis; Dyn: psychodynamic therapy; Eas: East Asia; Eu: Europe; Grp: group; Gsh: guided self-help; Ind: individual; Ipt: interpersonal psychotherapy; Itt: intention-to-treat; Lrt: life review therapy; M age: mean age; Med: general medical patients; Mood: diagnosed mood disorder; N arms: number of arms; N outc: number of outcome measures; N sess: number of sessions; Old: older adults; Oth: other; other psy: other type of psychotherapy; ppd: perinatal depression; Pr wom: proportion women; Pst: problem solving therapy; Recr: recruitment; Rob: risk of bias; Sg: sequence generation; Sr: self-report; Stud: students; Sup: non-directive supportive counseling; Targ grp: target group; Ther: therapy; Uk: United Kingdom; Us: Unites States of America.

## Appendix C. Response rates

|  | *All studies* | | | | |  | *CAU controlled studies* | | | | |  | *WL controlled studies* | | | | |  | *p* |
| --- | --- | --- | --- | --- | --- | --- | --- | --- | --- | --- | --- | --- | --- | --- | --- | --- | --- | --- | --- |
|  | *k* | *Resp* | *95% CI* | *I^2^* | *95% CI* |  | *k* | *g* | *95% CI* | *I^2^* | *95% CI* |  | *k* | *g* | *95% CI* | *I^2^* | *95% CI* |  |  |
| Response rates within control conditions | | | | | | | | | | | | | | | | | | | |
| All studies | 280 | 0.17 | 0.16; 0.19 | 73 | 69; 76 |  | 152 | 0.19 | 0.17; 0.21 | 78 | 74; 81 |  | 128 | 0.16 | 0.14; 0.18 | 55 | 45; 63 |  | <0.001 |
| Outliers excluded ^a)^ | 277 | 0.17 | 0.16; 0.18 | 17 | 1; 30 |  | 117 | 0.19 | 0.18; 0.20 | 34 | 16; 47 |  | 114 | 0.16 | 0.15; 0.17 | 2 | 0; 20 |  | ^b)^ |
| Only low RoB | 103 | 0.18 | 0.17; 0.20 | 70 | 63; 75 |  | 57 | 0.21 | 0.18; 0.24 | 73 | 65; 79 |  | 46 | 0.16 | 0.14; 0.19 | 60 | 45; 71 |  | <0.001 |
| Adjusted for pubbias | 383 | 0.24 | 0.22; 0.26 | 77 | 74; 79 |  | 206 | 0.26 | 0.23; 0.29 | 82 | 80; 84 |  | 174 | 0.19 | 0.17; 0.22 | 60 | 53; 66 |  | <0.001 |
| Response rates within therapy conditions | | | | | | | | | | | | | | | | | | | |
| All studies | 407 | 0.39 | 0.37; 0.42 | 83 | 81; 84 |  | 200 | 0.36 | 033; 0.40 | 88 | 86; 89 |  | 207 | 0.42 | 0.39; 0.45 | 71 | 67; 75 |  | 0.27 |
| Outliers excluded ^a)^ | 268 | 0.38 | 0.37; 0.40 | 28 | 16; 39 |  | 121 | 0.35 | 0.33; 0.37 | 46 | 33; 56 |  | 161 | 0.41 | 0.39; 0.43 | 23 | 6; 37 |  | ^b)^ |
| Only low RoB | 139 | 0.37 | 0.34; 0.41 | 85 | 83; 87 |  | 77 | 0.38 | 0.33; 0.43 | 88 | 85; 90 |  | 62 | 0.37 | 0.32; 0.41 | 79 | 74; 84 |  | 0.03 |
| Adjusted for pubbias | 429 | 0.37 | 0.35; 0.40 | 85 | 83; 86 |  | 210 | 0.39 | 0.35; 0.42 | 88 | 87; 89 |  | 245 | 0.36 | 0.33; 0.39 | 78 | 75; 80 |  | 0.32 |

Abbreviations: CI: confidence interval; g: standardized mean difference (Hedges’ g); k: number of studies; Pubbias: publication bias; Resp: response rate; RoB: Risk of Bias.

a) subgroup analyses were done with the comparisons of the main analyses (so outliers were not separately excluded from each subgroup)

b) the effect sizes for the two subgroups were calculated separately for each subgroup, so the p-value for the difference could not be calculated

## Appendix D. Metaregression analyses of response rates within treatment and control conditions

|  | *Response rates within control groups* | | |  | *Response rates within therapy groups* | | |
| --- | --- | --- | --- | --- | --- | --- | --- |
|  | *Coeff* | *SE* | *p* |  | *Coeff* | *SE* | *p* |
| WL vs CAU | -0.28 | 0.12 | 0.02 |  | 0.27 | 0.11 | 0.01 |
| IPT included | 0.19 | 0.20 | 0.36 |  | -0.07 | 0.19 | 0.73 |
| 3rd wave included | -0.16 | 0.18 | 0.38 |  | 0.06 | 0.16 | 0.70 |
| Individual format incl | 0.32 | 0.12 | 0.01 |  | 0.29 | 0.10 | 0.01 |
| Telephone format incl | 0.12 | 0.24 | 0.63 |  | 0.30 | 0.23 | 0.18 |
| Guided self-help incl | 0.24 | 0.13 | 0.06 |  | -0.22 | 0.12 | 0.07 |
| Two arms vs more than two | -0.04 | 0.14 | 0.79 |  | -0.11 | 0.11 | 0.30 |
| One measure vs more | 0.09 | 0.11 | 0.41 |  | -0.01 | 0.09 | 0.89 |
| Publication year | -0.01 | 0.01 | 0.15 |  | -0.02 | 0.01 | 0.00 |
| Target group: Adults in general | Ref. |  |  |  | Ref. |  |  |
| Target group: General medical | -0.13 | 0.16 | 0.42 |  | -0.31 | 0.15 | 0.04 |
| Target group: Older adults | -0.31 | 0.21 | 0.13 |  | -0.31 | 0.18 | 0.08 |
| Target group: Perinatal depression | -0.10 | 0.19 | 0.58 |  | -0.03 | 0.18 | 0.87 |
| Target group: Students | -0.14 | 0.23 | 0.54 |  | 0.18 | 0.21 | 0.41 |
| Target group: Other | -0.32 | 0.17 | 0.06 |  | 0.03 | 0.16 | 0.86 |
| Recruitment: Clinical | Ref. |  |  |  | Ref. |  |  |
| Recruitment: Community | -0.18 | 0.15 | 0.22 |  | 0.12 | 0.14 | 0.38 |
| Recruitment: Other | -0.22 | 0.16 | 0.17 |  | 0.27 | 0.15 | 0.08 |

Abbreviations: Coeff: coefficient; SE: standard error.
